# Expression Signature as a Biomarker for Prenatal Diagnosis of Trisomy 21

**DOI:** 10.1371/journal.pone.0074184

**Published:** 2013-09-16

**Authors:** Marija Volk, Aleš Maver, Luca Lovrečić, Peter Juvan, Borut Peterlin

**Affiliations:** 1 Clinical Institute of Medical Genetics, Department of Obstetrics and Gynecology, University Medical Center, Ljubljana, Ljubljana, Slovenia; 2 Center for Functional Genomics and Bio-Chips, Institute of Biochemistry, Faculty of Medicine, University of Ljubljana, Ljubljana, Slovenia; IGBMC/ICS, France

## Abstract

A universal biomarker panel with the potential to predict high-risk pregnancies or adverse pregnancy outcome does not exist. Transcriptome analysis is a powerful tool to capture differentially expressed genes (DEG), which can be used as biomarker-diagnostic-predictive tool for various conditions in prenatal setting. In search of biomarker set for predicting high-risk pregnancies, we performed global expression profiling to find DEG in Ts21. Subsequently, we performed targeted validation and diagnostic performance evaluation on a larger group of case and control samples. Initially, transcriptomic profiles of 10 cultivated amniocyte samples with Ts21 and 9 with normal euploid constitution were determined using expression microarrays. Datasets from Ts21 transcriptomic studies from GEO repository were incorporated. DEG were discovered using linear regression modelling and validated using RT-PCR quantification on an independent sample of 16 cases with Ts21 and 32 controls. The classification performance of Ts21 status based on expression profiling was performed using supervised machine learning algorithm and evaluated using a leave-one-out cross validation approach. Global gene expression profiling has revealed significant expression changes between normal and Ts21 samples, which in combination with data from previously performed Ts21 transcriptomic studies, were used to generate a multi-gene biomarker for Ts21, comprising of 9 gene expression profiles. In addition to biomarker’s high performance in discriminating samples from global expression profiling, we were also able to show its discriminatory performance on a larger sample set 2, validated using RT-PCR experiment (AUC=0.97), while its performance on data from previously published studies reached discriminatory AUC values of 1.00. Our results show that transcriptomic changes might potentially be used to discriminate trisomy of chromosome 21 in the prenatal setting. As expressional alterations reflect both, causal and reactive cellular mechanisms, transcriptomic changes may thus have future potential in the diagnosis of a wide array of heterogeneous diseases that result from genetic disturbances.

## Introduction

Progressive development and improvement of methods for investigation of global transcriptional alterations in human disease is now enabling reproducible and consistent selection of genes that best differentiate samples originating from disease-affected or healthy individuals [1]. Although transcriptome is a highly complex and dynamic system, difficult to model with classical approaches, it does nevertheless present a landscape where manifold pathogenic and reactive processes occurring in disease may be detected. As transcriptional regulation results from genetic as well as environmental influences, transcriptomics present a valuable opportunity for the development of a heterogeneous diagnostic tool for diseases ranging from those of clear genetic aetiology to those of complex unexplained aetiology.

Identification of high-risk pregnancy with its heterogeneous aetiology and complex pathogenesis remains complicated since there is no single investigation deemed to be best in all circumstances. As there already are successful implementations of expression biomarkers into clinical practice [2] we opted to investigate and demonstrate the feasibility of expression biomarkers to predict high-risk pregnancy status on Ts21 as a model of high-risk pregnancy. The objective for selecting Ts21 as a model system is its definite genome-phenome correlation. Several global gene expression studies of Ts21 demonstrated extensive changes in expression of chromosome 21 (HSA21) and non- chromosome 21 (non-HSA21) genes [3-10].

Global transcriptome profiling studies on prenatal samples of amniotic fluid and chorionic villi with trisomy of chromosome 21 [3,5] revealed significant dysregulation of HSA21 and non-HSA21 genes and concluded that resulting alterations reflect a combination of gene dosage effect and genome-wide transcriptional dysregulation hypotheses. Reports on transcriptomic analyses of other fetal tissues [4,6,10], including cerebellum, heart or fibroblasts demonstrated that sets of over-expressed and under-expressed genes differ across different cell types. In addition, data from global gene expression studies on postnatal samples with Ts21 [7-9], including adult human brain, lymphoblastoid cell line or fibroblasts showed a profile of up-regulation of HSA21 and dysregulation of non-HSA21 genes, consistent with the results of transcriptomic studies on Ts21 prenatal samples.

These studies mainly addressed the issue of transcriptomic changes in trisomy of chromosome 21 in different human tissues mainly with the aim to dissect Down syndrome phenotype and to explain the pathogenic mechanisms underlying variability of Down syndrome. However, they did not primarily aim to assess the diagnostic potential of transcriptomic changes in trisomy of chromosome 21 samples.

One problematic aspect of aforementioned studies was also that the reported results overlapped only partially and were therefore difficult to reproduce, probably due to small study power stemming from low numbers of biological replicates investigated and large heterogeneity of analysed tissues across various studies. However, a recent meta-analysis from 45 heterogenous publicly available Ts21 datasets succeeded to organize these results and identify a pattern of consistency in dysregulated genes from Ts21 studies [11].

Among the aforementioned transcriptomic studies only a few investigated transcriptomic alterations in prenatal Ts21 samples, including chorionic villi, amniotic fluid cell-free mRNA or amniocytes [3,5,12-14]. The rationale for selecting amniocyte samples is in investigating the gene expression profile of a developing fetus. The heterogeneity of cells derived from different fetal tissues increases not only the biological variability, but also the possibility of identifying an expression biomarker that would discriminate between Ts21 and a normal euploid samples.

We aimed to design a discriminatory gene expression signature by a 2-stage approach. Firstly, in the *discovery stage*, we performed detection of differentially expressed genes (DEG) by global gene expression profiling and evaluated these results in the context of existent body of literature. Based on this step, a subset of genes was incorporated in the core gene set for further analyses. In the second, *validation stage*, the performance of biomarker gene set was evaluated on an independent study sample. Additionally, the biomarker set performance was tested on processed datasets from the GEO database.

## Results

### Discovery stage

Results from global profiling of gene expression in cultured amniocyte samples suggest that chromosome trisomy causes a considerable perturbation of complete cellular transcriptional composition in the amniocyte cells. Altogether, we have detected altered levels of mRNAs transcribed from 964 unique genes, accounting for 4.9% of complete set of genes investigated using microarray profiling in the discovery phase. Among these, an apparently increased tendency towards differential expression was observed for HSA21 genes, with 32 such genes showing significant deregulation in trisomy 21 amniocytes (Table S1). This subset represented a significant enrichment of HSA21 genes among DE genes, with significant over-representation (p=1.9·10^-14^), according to hypergeometric distribution test. This subset of genes was characterized by average fold change of 1.59 (95% CI 1.39-1.82). A considerable proportion of HSA21 genes (87.1%), however, appeared to resist the dosage effect and their expression level, while showing slight tendency towards up-regulation (their FC values averaged at 1.03, with 95% CI 1.01-1.04), did not display significant changes in comparison to controls after significance values were corrected for multiple testing. Altogether, 240 genes satisfied (Table S2) our predefined criteria, attaining false-discovery rate values below 0.05 and 50% reduction or increase in expression in Ts21 amniocytes, and were thus regarded as candidate genes for inclusion in the core biomarker gene set.

**Figure 1:**
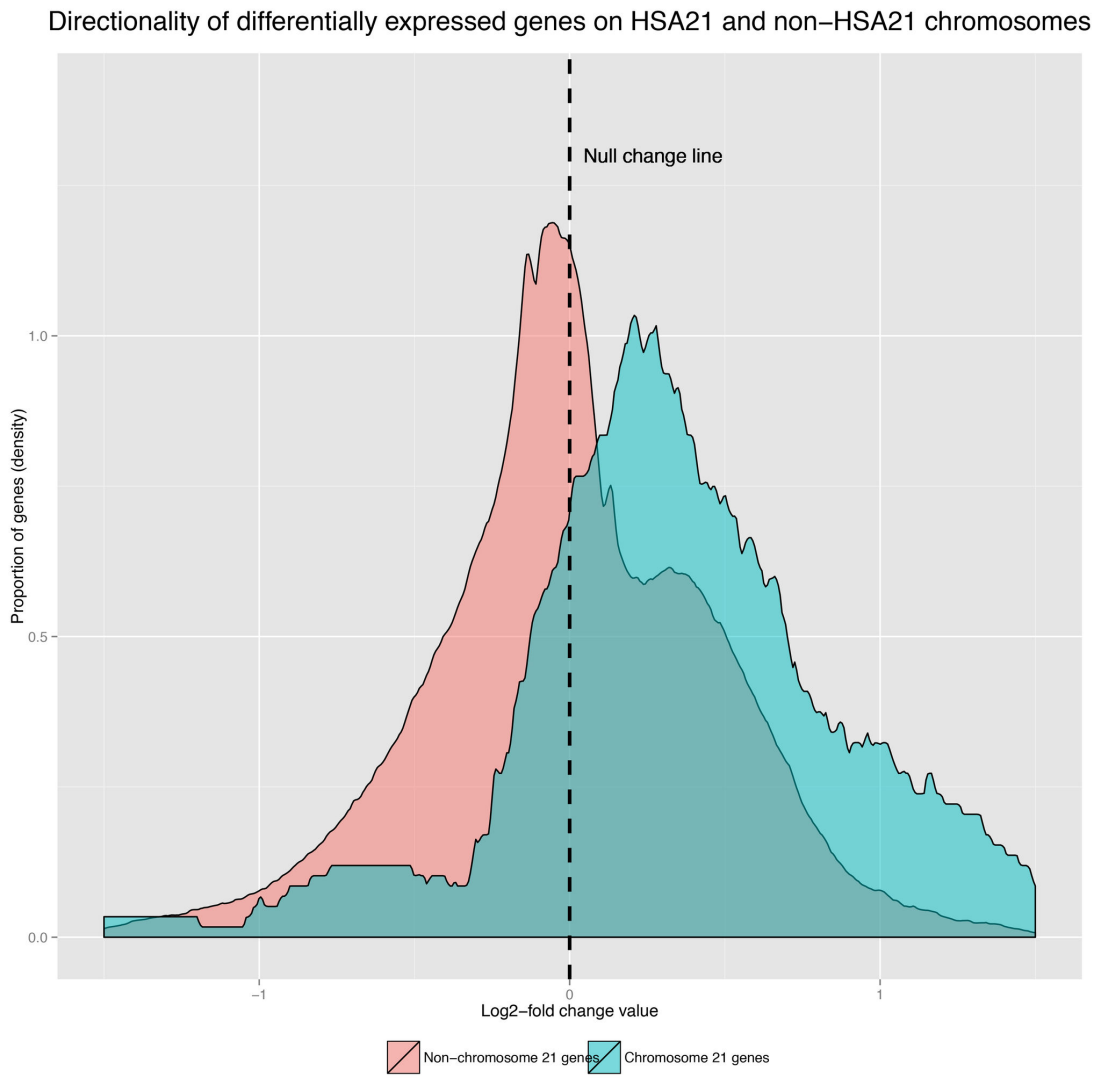
The directionality of HSA21 differentially expressed genes in comparison to non-HSA21. A pattern of upregulation is present in differentially expressed HSA21 genes, while non-HSA21 genes tended to be slightly down-regulated.

In addition to differential expression of genes on chromosome 21, we have detected a widespread transcriptional response of 932 non-HSA21 genes (Table S3), which were equally distributed in the genome without any observable per-chromosome overrepresentation (after Bonferroni correction, overrepresentation of genes for all chromosomes failed to reach the significance of 0.05, with the exception of chromosome 21). Interestingly, the overall average tendency of non-HSA21 genes was directed toward down-regulation, a phenomenon observed with greatest prominence in genes with the greatest transcriptional alterations in Ts21 (adjusted p-values below 0.01). In Figure 1 the density estimation of fold change values for genes located on HSA21 and those located on a non-HSA21 location may be observed, with apparent differences between directionality of the response for HSA21 and non-HSA21 genes. A tendency of all genes with highly active expression towards transcriptional decompensation in Ts21 amniocytes has been observed with Pearson rank correlation test confirming a relation between gene’s expression level and susceptibility to changes in dosage (p<1·10^-16^).

Subsequently, the classification properties of obtained microarray results were inspected. The clustering analyses have shown spontaneous separation of samples into two larger groups, with separation clearly corresponding to Ts21 status (Figure S1). We verified the actual classification performance by using cross-validation techniques, which have shown excellent prediction performance of the selected genes using 5-fold cross-validation of SVM classifier (AUC of 1.0 with 10 genes in the model). The performance of the classifier trained on permuted class information averaged at 0.42 (Figure S2).

### Validation on data from quantitative real-time PCR experiments

**Figure 2:**
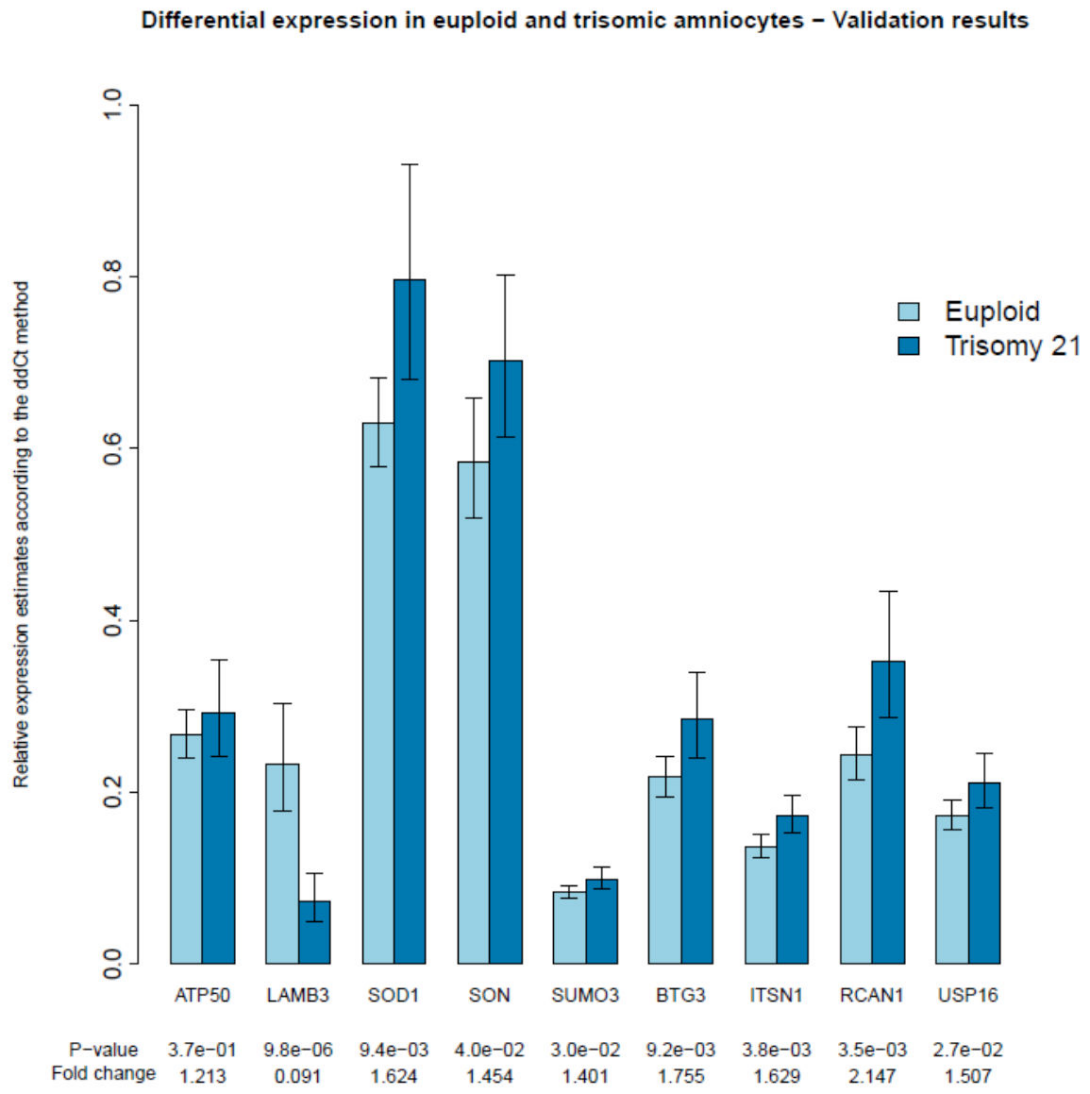
Differential expression of genes in the validation core set, where light blue bars represent average expression in euploid samples and dark blue in samples with trisomy 21. Confidence intervals of 95% for the expression mean based t-ditribution are also presented.

Based on results obtained in the discovery phase in addition to information from previously performed study a core set of classifying genes was selected and included in the core biomarker set (Table 1). Expression profiling of these genes has shown that differential levels of expression could be reproduced on a larger and independent replication sample of patients. With the exception of the ATP5O gene, all the genes taken to validation step displayed significant differential expression in the anticipated direction (7 DE genes from chromosome 21 with fold change values ranging from around 1.2-2.2), while for LAMB3 consistent down-regulation was observed in trisomy 21 samples with fold change values estimated at 0.09. Comparison between gene expression levels in the two study groups along with fold change values and t-test significance values are presented in Figure 2.

**Table 1:** Validation set characteristics, based on data from discovery stage in this study, in addition to data from other gene expression profiling studies.

Entrez GeneID	Gene Symbol	Gene name	Chromosomal location	Fold change	FDR ^♯^	Supporting evidence from other studies	Function
539	ATP5O	ATP synthase, H+ transporting, mitochondrial F1 complex, O subunit	21q22.1-q22.2	2.29	8.20E-04	[4,9,10]	energy metabolism
6651	SON	SON DNA binding protein	21q22.11	2.51	4.32E-02	[4,7,9]	regulator of cell-cycle
6647	SOD1	superoxide dismutase 1, soluble	21q22.11	2.29	2.52E-05	[4,7,9,10,20]	involved in ROS metabolism
6453	ITSN1	intersectin 1 (SH3 domain protein)	21q22.1-q22.2	2.11	1.79E-02	[3,7,10]	actin assembly and trafficking
6612	SUMO3	SMT3 suppressor of mif two 3 homolog 3	21q22.11	2.53	4.33E-03	[3,4,9]	involved in ROS metabolism
10950	BTG3	BTG family, member 3	21q21.2	2.14	4.51E-03	[3,4,7,9]	role in neurogenesis
1827	RCAN1	regulator of calcineurin 1	21q22.12	2.33	4.58E-02	[3,7,9,10]	role in neurogenesis
10600	USP16	ubiquitin specific peptidase 16	21q22.11	3.24	4.78E-03	[4,7,9]	involved in ROS metabolism
3914	LAMB3	laminin, beta 3	1q32	0.16	5.79E-03	[7]*	laminin is a basement membrane protein

♯ FDR – stands for “false discovery rate”, proportion of anticipated false discoveries, according to Benjamini-Hochberg method of adjusting for multiple testing

* Although LAMB3 was not identified in other studies, we incorporated it into our biomarker set, due to its marked and highly significant down-regulation in TS21 samples. Additionally, its down-regulation was not directly reported in [7], but it could nevertheless be detected in the same processed dataset in the Gene Expression Atlas (http://www.ebi.ac.uk/gxa/)

**Figure 3:**
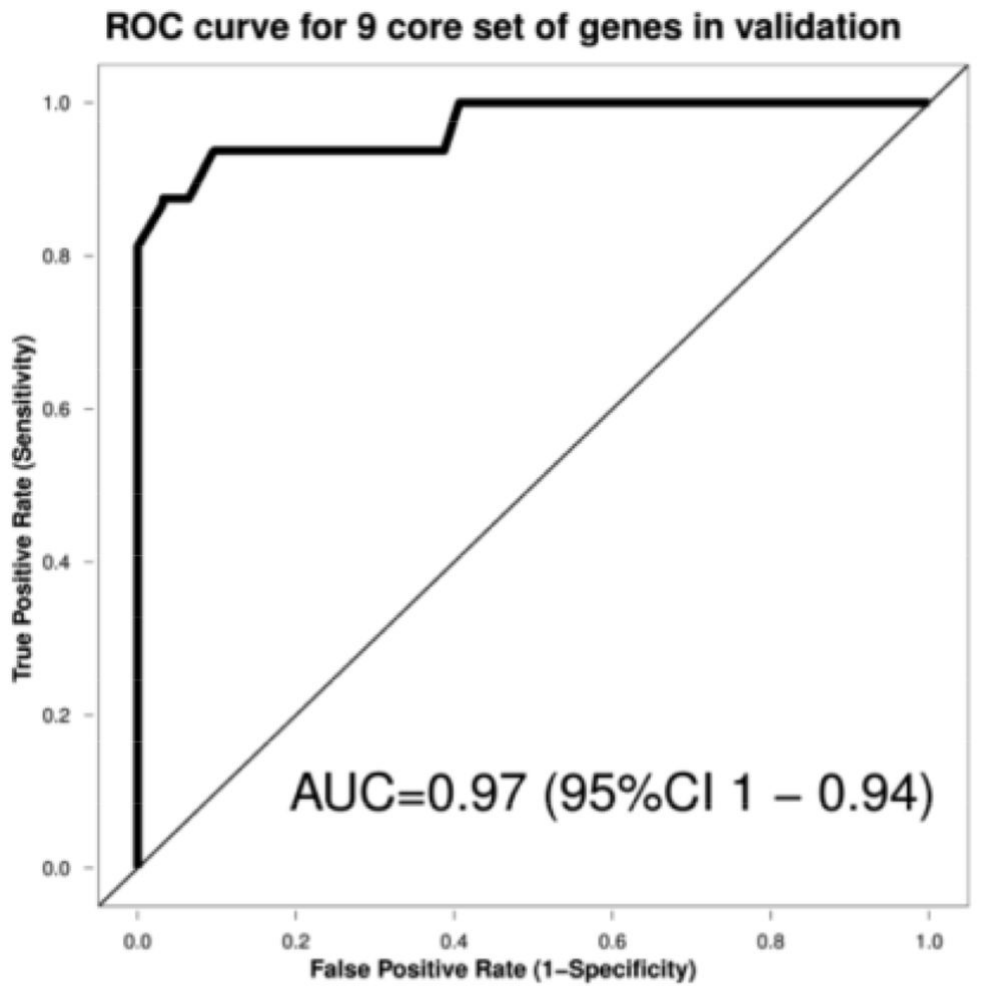
Random operator curve analyses, based on data from the RP-PCR validation stage.

Performance of the core gene expression set in the validation sample was estimated afterwards. The performance of SVM estimated using a leave-one-out cross-validation approach (LOOCV) resulted in AUC values of 0.97 for discrimination between euploid and trisomic samples (Figure 3).

### Validation on previously published microarray studies

**Figure 4:**
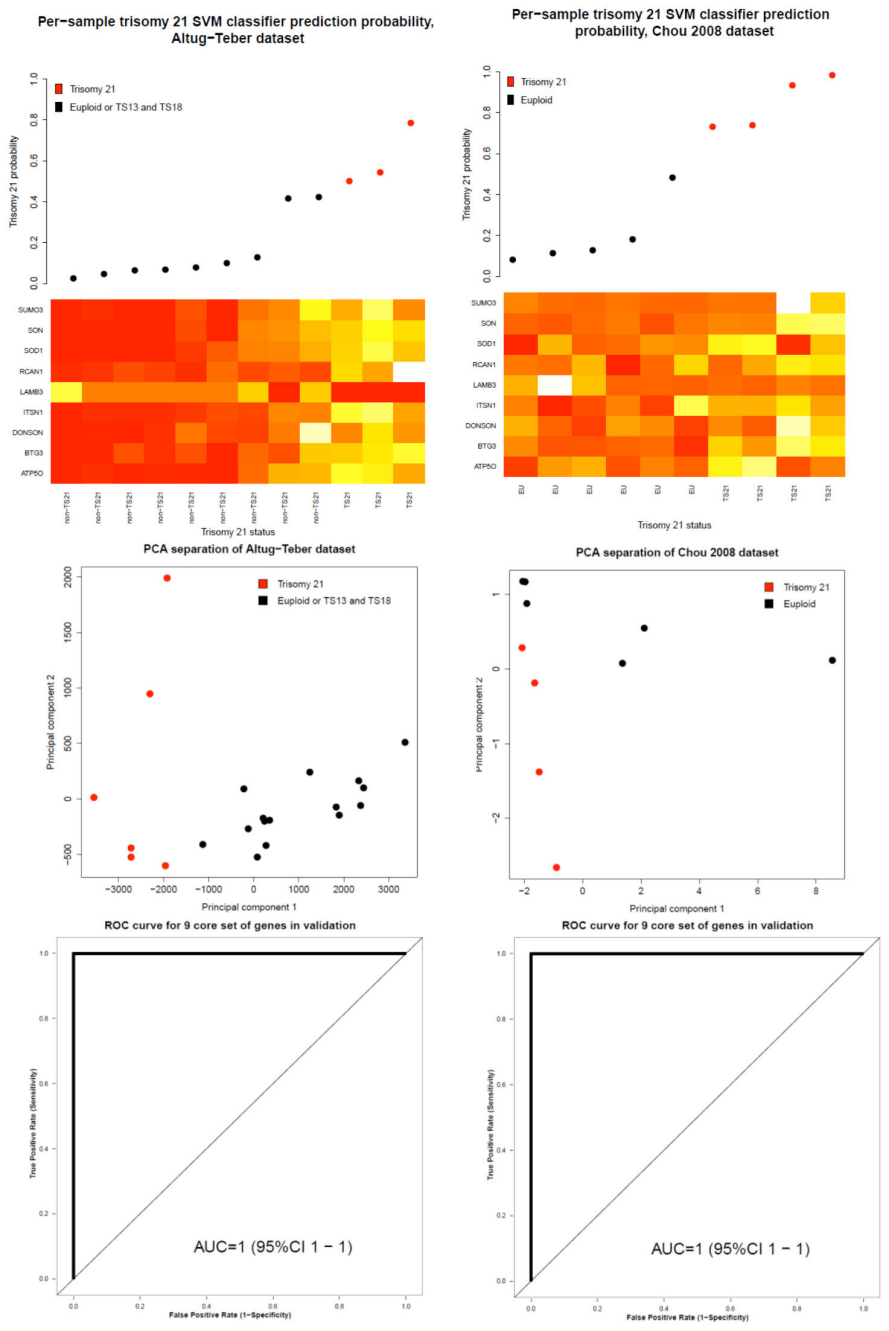
Validation of results, based on data from studies by Altug-Teber et al [3] and Chou et al [13] In the upper part of the figure, the per-sample predictions based on the expression profiles of 9 gene biomarker are displayed, with predicted probability of positive Ts21 status presented on y axis. The color of the dots represent the actual karyotyping diagnostic result. Below, heatmap representing gene expression level for each sample (heatmap rows) and for each gene (heatmap columns) is presented. The expression of 8 genes tends to be comparatively increased in samples with true trisomy 21 status, while expression of LAMB3 is directed oppositely, towards down-regulation in Ts21 samples. In the heatmap yellow color represents higher expression and red color lower expression level, where values have been scaled separately per each row. Middle part of the figure represents principal component analysis of 9-gene expression for samples expression profiles published in [3,13]. In the bottom part, plots representing ROC-based classification performance of 9-gene expression biomarker may be observed.

Further support for the true performance of gene expression biomarker for classification was obtained from previously performed global gene expression profiling studies of trisomy 21 performed on prenatal samples. The predictive performance of our 9-gene set was high in each dataset, reaching AUC values of 1.0 for both Altug-Teber and Chou dataset (Figure 4). The high performance of our gene set in Altug-Teber datasets demonstrates its high robustness, as our biomarker could differentiate trisomic samples from control ones in chorionic villus samples, even though gene selection in the discovery stage was wholly based on data from amniocyte profiling.

## Discussion

In this study we demonstrate the potential of gene expression signature, consisting of 9 genes, in prediction of Ts21 status in the prenatal setting.

In contrast to previously performed studies investigating transcriptional alterations in Ts21 which mainly focused on detecting transcriptome alterations or explaining the variability of DS phenotype [3,5,12-14], our main objective was to identify gene expression signature for discrimination of Ts21 from euploid status. We show that gene expression signature consisting of 9 genes could discriminate Ts21 cases from normal samples (AUC performance estimate reached 0.97 for discrimination of Ts21 samples from controls). We have also demonstrated the high discriminatory performance of the same 9-gene expression signature on data from two previous transcriptome profiling studies, measured either in amniocyte samples or chorionic villus biopsy samples [3,13].

While initial studies on expression alterations in Ts21 reported discordant results, Vilardell and coworkers have recently performed a meta-analysis of several Ts21 expression studies [11], including those performed in various tissue types and were able to demonstrate the existence of consistent gene expression alterations occurring in Ts21. This is in accordance with our observation, suggesting good consistency of gene expression data, even across different microarray platforms and laboratories or bioinformatic workflows.

The effect of an additional copy of HSA21 has previously been thought to result in 1.5 times upregulation of gene expression of HSA21 genes. This has been in part refuted by large-scale studies investigating whole chromosome 21 and whole genome expression perturbances in trisomy 21, when a requirement for a better pathogenetic model has surfaced to explain complex transcriptional alterations detected on global scale. To rectify these drawbacks and to facilitate the selection of genes for best discrimination of prenatal samples with trisomy 21 from those with euploid chromosomal constitution, a two-tiered gene expression profiling study was performed, consisting of a discovery and validation phase. The discovery phase was performed utilizing global microarray expression profiling on the largest collection of trisomy 21 and control euploid prenatal amniocyte samples investigated to date. To optimize gene selection, we observed data originating from studies previously performed on amniocytes [3,4,10]. Additionally, we also considered datasets from studies performed on other tissues, such as chorionic villous cells, brain and blood samples, as constitutional presence of an additional HSA21 chromosome has been shown to cause a subset of tissue-independent gene expression alterations [11]. Genes that were found differentially expressed in our study, and have been consistently detected in at least three other gene expression-profiling studies were then carried to second validation phase.

We also recognize some limitations of our study. Firstly, the classification performance of the proposed gene expression signature was high, AUC values reaching 0.97. However, we believe that with introduction of additional genes in the signature, the performance could likely be further improved. Secondly, the expression profiling experiments in our study were performed on the samples of cultivated amniocytes, which might influence actual expression alterations taking place in Ts21. Nevertheless, since we could demonstrate high accuracy of the same signature on the uncultivated chorionic villus biopsy tissue sample set [3], it could be anticipated that cultivation would not influence the predictive power of our expression signature in an important manner. Thirdly, although we have shown high discriminatory performance of the gene expression test and performed analytic validation of expression signature in Ts21, further studies should be performed prior to actual introduction of the test into the clinical practice and implementation should be accomplished in accordance with standards of quality assurance [15].

Although this research was focused on the delineation of consistent alterations present in trisomy 21 and definition of a biomarker with potential in clinical use, the utility of expression profiling may pervade other pathological entities encountered in the prenatal setting. As gene profile is not solely determined by the genetic constitution of an individual, but is the sum of genomic, epigenomic and environmental influences, its utility may surpass detection of Ts21 presented here and may present a possible multifaceted biomarker of high risk pregnancies.

## Materials and Methods

### Ethics statement

This study was approved by Slovenian National Ethics Commitee. All participants have signed an informed consent prior to participation in the study and clinical investigations have been conducted in accordance with principles expressed in Helsinki declaration.

### Cell culture samples

Amniotic fluid samples were collected between 16^th^ and 18^th^ week of gestation for routine cytogenetic analysis. Primary cell cultures of amniotic fluid were performed according to standard protocols using tissue culture flasks (TPP, Switzerland). Cell cultures were grown in Amnio Max C100 Basal Medium and Amnio Max C100 Supplement (Invitrogen, CA, USA) at 37°C in 5% CO_2_ environment. Cytogenetic analysis was performed according to standard protocol with a resolution of 450-550 bands per haploid set. Following a routine diagnostic analysis a second passage of amniotic cell culture was grown in the same condition as primary cell culture. The gene expression discovery group (sample set 1) comprised of 19 samples, namely 10 derived from fetuses with trisomy 21 and 9 coming from normal pregnancies (Table S4). The validation group (sample set 2) consisted of an independent set of 48 amniotic fluid samples, namely 16 cases with Ts21 (Table S5) and 32 controls. The control group consisted of 32 samples with normal karyotypes and normal ultrasound scans, collected at 16-18 weeks gestation. Cell suspension was centrifuged at 2500rpm for 8minutes and supernatant was removed. Pelleted cells were processed further according to the manufacturers recommendation for RNA isolation.

### RNA isolation

Isolation of RNA from cultured amniocytes was performed using the Fujifilm QuickGene-810 automated isolation system (Fujifilm Life Sciences, Tokyo, Japan), using columns in the Fujifilm RNA Cultured Cell kit to capture purified RNA samples. The purity and yield of isolated RNA samples was determined using NanoDrop 2000c spectrophotometer (Nanodrop Technologies, Wilmington, DE, USA).

The integrity of RNA samples isolated from subjects selected for genome-wide profiling of gene expression was investigated on Agilent’s Bioanalyzer using RNA 6000 Nano Kit, where only samples attaining RNA integrity number (RIN) values greater than 8.0 were used for downstream array experiments.

### Development of gene expression signature for discrimination of Ts21 samples from controls

In the present study we have constructed and evaluated potential gene expression signature for Ts21 in two separate stages.

Initially, in *discovery stage* of our study, we aimed to identify the gene expression signature for Ts21, consisting of a minimal set of genes with predictive diagnostic power for discrimination of Ts21 cases from controls.

Subsequently, in the *validation stage* of this study, the expression signature of genes selected in the discovery stage was validated on an independent group of samples. We employed two different approaches to validation of samples – firstly, we proposed validation of gene signature on an independent sample of Ts21 cases and controls by means of quantitative PCR and secondly, we have validated the discriminatory performance of the signature on data from two previously performed independent genome-wide expression profiling studies [3,13].

### Discovery stage – global gene expression profiling

Agilent’s 4x44 two-colour Whole Human Genome Expression arrays, containing 41.001 feature probes for interrogation of over 19.644 human genes, were selected to estimate the extent of global transcriptional alterations in investigated cells. Preparation of RNA samples, their labelling and hybridization were performed according to manufacturer’s instructions. For the purposes of microarray study, test samples and control samples from sample set 1 were hybridized against a common reference, obtained by pooling all samples. Here, the reference RNA pool was labelled with Cy3 and each sample was labelled separately with Cy5 [16].

Microarray slides were scanned using GenePix 4100A microarray scanner. Post-processing steps included intra-array loess and inter-array quantile normalization to correct for potential bias resulting from differential stability of cyanine dyes. Fluorescent values were offset by 100 units to reduce the anomalous dispersion of fold change (FC) values at lower signal intensities. MA and multidimensional scaling (MDS) plots were inspected for each array to detect any systemic error resulting from preceding steps.

Subsequently, results were statistically analyzed using a linear model fit in limma package for Bioconductor in an R statistical environment. To account for multiple testing, obtained significance values were corrected using the Benjamini-Hochberg method and the adjusted significance threshold set at α<0.05.

### Selection of classifying core gene set

Genes that would be included in the core biomarker gene set were selected based on multiple lines of evidence. For inclusion, the gene would have to be differentially expressed in the discovery phase, with significance values below the adjusted p-value threshold. Additionally, to technically facilitate subsequent validation, the gene’s expression levels should be altered by at least 50% (under-expressed or over-expressed).

To expand the information landscape used for gene selection, data from previous studies were incorporated into the gene selection process. Only genes, detected in at least 3 other studies and our study were considered to have enough consistent differential expression to be included in the core gene set (Table 1).

### Validation stage – testing of core gene set on independent set of samples

A core set of genes selected in the discovery phase was investigated in a validation step on an independent set of samples using real-time PCR quantification of gene expression. For this purpose, RNA samples passing quality checks were subjected to reverse transcription using the Superscript Vilo reverse transcriptase (Invitrogen, Carlsbad, CA, USA) according to manufacturer’s instructions. Afterwards, gene expression was quantified using pre-designed TaqMan assays (Applied Biosystems, Foster City, CA, USA) with assay identification numbers: Hs00533490_m1 for SOD1, Hs00426889_m1 for ATP5O, Hs00165078_m1 for LAMB3, Hs00739248_m1 for SUMO3, Hs00199064_m1 for BTG3, Hs00371372_m1 for SON, Hs00170791_m1 for USP16, Hs01120954_m1 for RCAN1, Hs00161676_m1 for ITSN1. Reactions were performed in volumes of 25µL, consisting of 12.5µLof 2x Universal Master Mix (Applied Biosystems), 1.25µL of a specific assay mix, 2µL of cDNA sample and 9.25µL of bidestilated water. Input cDNA samples were diluted so that an amount of cDNA equivalent to 50ng of RNA was used in each reaction. To minimize stochastic effects, all reactions were run in triplicates. RT-PCR reactions were performed on ABI Prism 7000 Sequence detection system (Applied Biosystems). Thermal cycling conditions were as follows: 50°C for 2 min, 95°C for 10 min and 40 cycles of 95°C for 15s and 60°C for 1 min. The threshold cycle (Ct) values were then determined for each assay and were normalized to internal control (ACTB gene, assay identification number:Hs03023880_g1) that was co-run with each sample. Differences in gene expression between samples could then be calculated using the delta-deltaCt method, as previously described [17]. The significance of expression differences in the two groups investigated in the validation phase was calculated using two-sample t-distribution test, and differences were deemed significant at α <0.05.

### Validation on previously performed global profiling studies

To further investigate the discrimination properties of our biomarker gene set, the complete (normalized and pre-processed) datasets from two studies were obtained from Gene Expression Omnibus (GEO) repository [18], namely GSE6263 [3] and GSE10758 [13]. Here, only genes in the core biomarker set were further investigated and used for biomarker performance estimation. 

### Evaluation of expression biomarker classification performance

Statistical evaluation of the expression biomarker’s classification performance was performed using the classification toolset from CMA package for R [19]. In all cases probabilistic support vector machine classification linear kernel function was used for training of the model and subsequent predictions. The classification performance was estimated using the area under curve (AUC) value, based on random operated curve (ROC) estimation.

Evaluation of global gene expression profile based classification on microarray data was performed using automated feature prioritization based on t-test statistic, and 5-fold cross validation for repeated separation of samples into training and testing subsets. To avoid over-training of classifier function on microarray samples, the feature selection step was repeated in each cross-validation sample. To further exclude the possibility of over-fitting, another test was performed, where sample status was permuted randomly and the distribution of prediction performances was investigated in this setting. In the absence of over-fitting bias, the prediction performance of the classifier should on average be non-discriminatory, with expected AUC values averaging at approximately 0.5.

The predictive performance of the gene set taken to the validation stage was also investigated using SVM function. Due to differences in platform utilized in gene expression profiling, a new classifier, based on RT-PCR results was constructed for this dataset. As the set of genes in the validation was predefined, no feature selection on the validation dataset was performed to avoid artificially inflating performances by injection the knowledge obtained from validation stage. The performance here was estimated by the leave-one-out cross validation technique and performance was again estimated using AUC.

The same procedural flow was also taken for evaluation of data originating from publicly available gene expression profiling studies, where classification performance was again evaluated using the 9 gene set included in our biomarker.

## Supporting Information

Figure S1 Unsupervised hierarchical clustering of samples, based on expression profiles of top 500 differentially expressed genes. A clear spontaneous separation between trisomy 21 and control samples may be observed, with red color representing up-regulation and green color representing down-regulation.
(TIFF)

Figure S2 Estimation of classification accuracy based on microarray data before continuation to RT-PCR validation step. The figure represents performance of classifier model based on top differentially expressed genes, estimated by 5-fold cross-validation. Red colored dots represent performance of classifier generated on actual samples status information, while blue dots represent performance of classifier learned on permuted sample classification. Classifier performance was also evaluated on progressively increasing number of genes included in the model (x-axis), where the best classification performance was attained in the range of 5-25 genes included in the model.
(TIFF)

Table S1 32 chromosome 21 genes found differentially expressed in trisomy 21 in comparison with euploid amniocyte samples.
(DOCX)

Table S2 240 candidate genes for inclusion in the core biomarker gene set, satisfying 2 criteria: attaining false-discovery rate values below 0.05 and 50% reduction or increase in expression in DS amniocytes.
(DOCX)

Table S3 932 non-chromosome 21 genes found differentially expressed in trisomy 21 in comparison with euploid amniocyte samples.
(DOCX)

Table S4 Clinical characteristics of the amniotic fluid samples (controls, T21) used for global expression profiling.
(DOCX)

Table S5 Clinical characteristics of the amniotic fluid samples with T21 samples used for validation in real-time quantification step.
(DOCX)
