# Supplementary figures and images for: Expression Signature as a Biomarker for Prenatal Diagnosis of Trisomy 21
Source: PLoS One. 2013 Sep 16;8(9):e74184. doi: 10.1371/journal.pone.0074184 (PMC3774664; doi:10.1371/journal.pone.0074184)

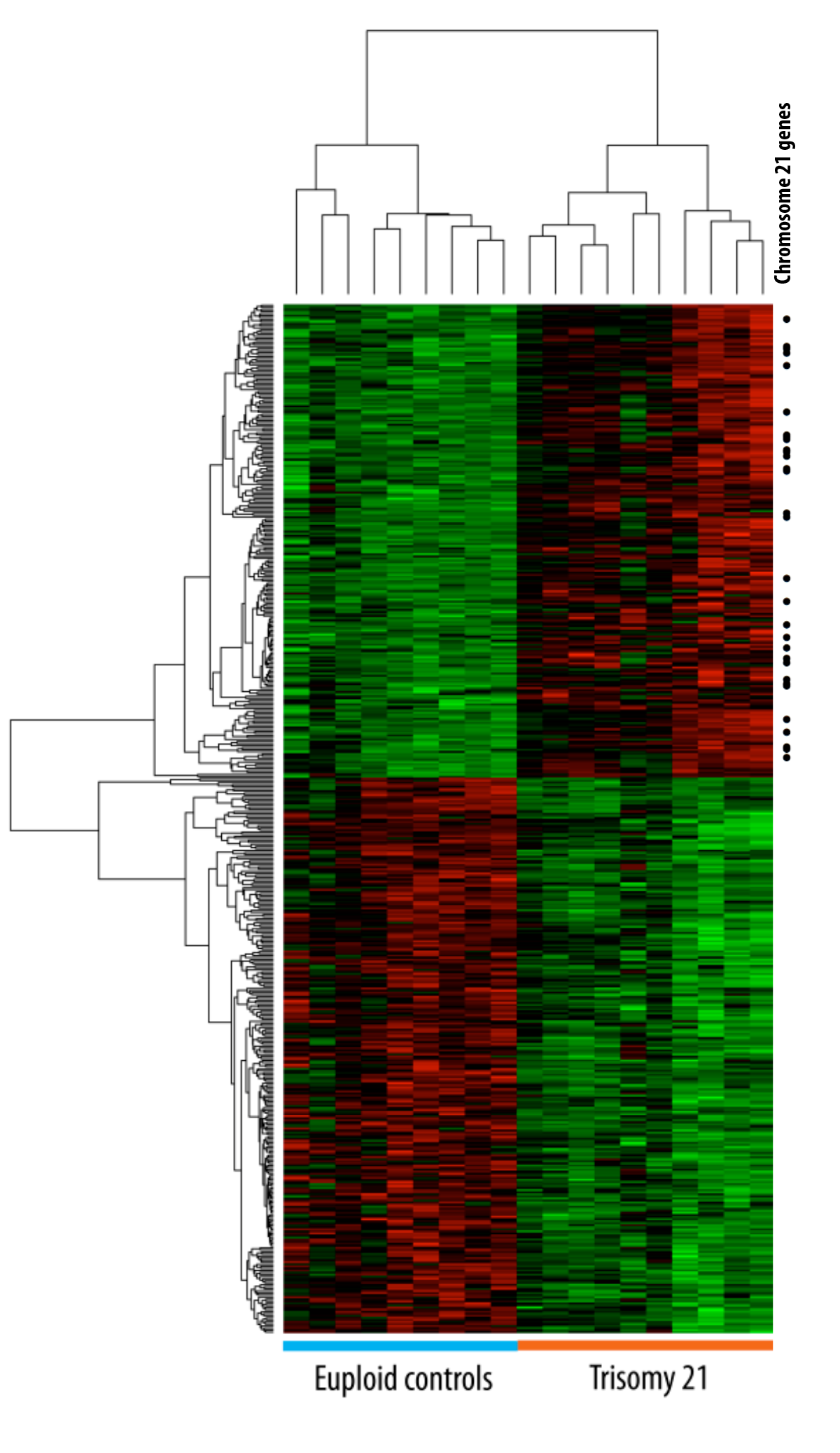

Supplement: Figure S1 — Unsupervised hierarchical clustering of samples, based on expression profiles of top 500 differentially expressed genes. A clear spontaneous separation between trisomy 21 and control samples may be observed, with red color representing up-regulation and green color representing down-regulation. (TIFF) [file pone.0074184.s001.tiff]

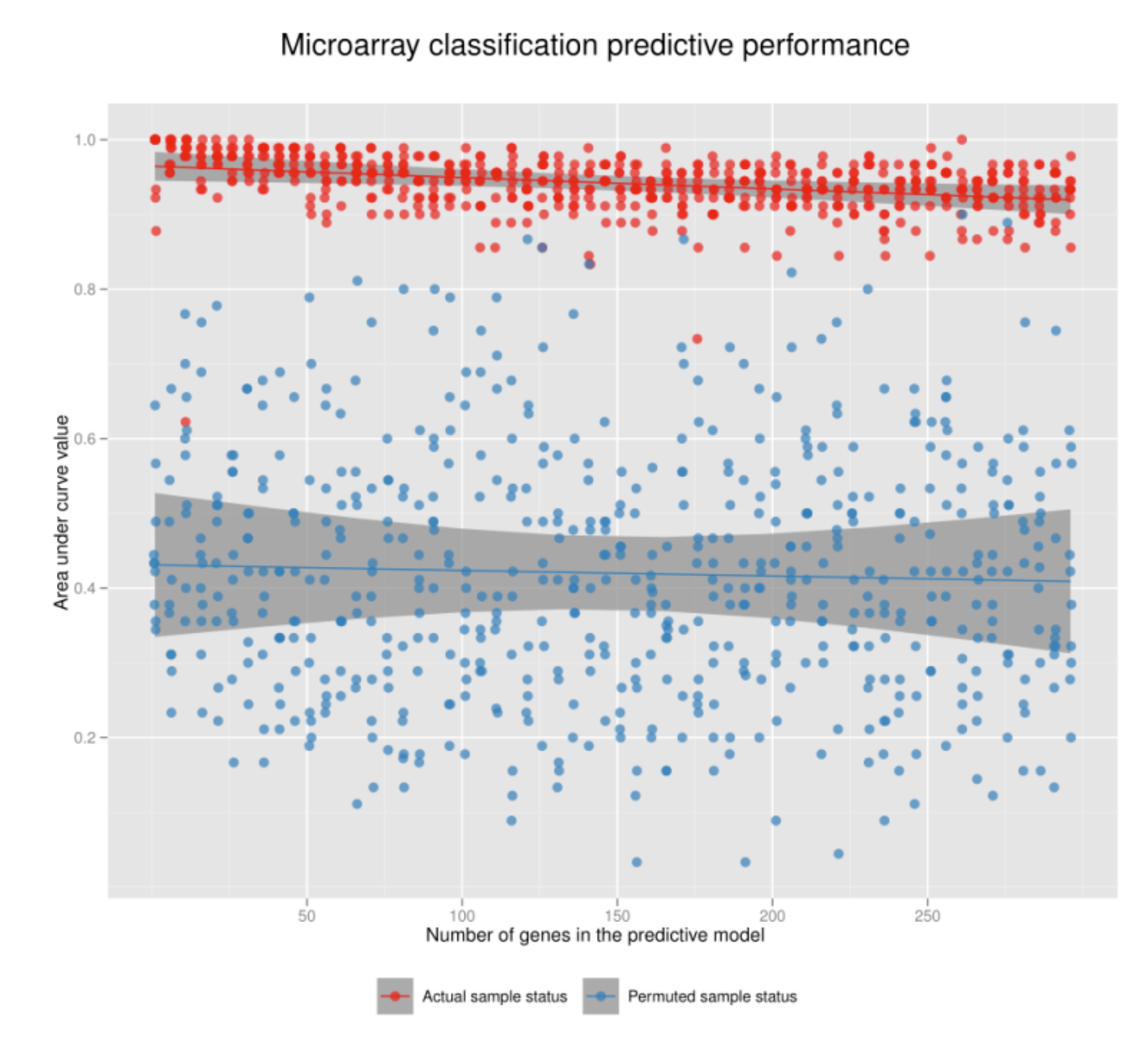

Supplement: Figure S2 — Estimation of classification accuracy based on microarray data before continuation to RT-PCR validation step. The figure represents performance of classifier model based on top differentially expressed genes, estimated by 5-fold cross-validation. Red colored dots represent performance of classifier generated on actual samples status information, while blue dots represent performance of classifier learned on permuted sample classification. Classifier performance was also evaluated on progressively increasing number of genes included in the model (x-axis), where the best classification performance was attained in the range of 5-25 genes included in the model. (TIFF) [file pone.0074184.s002.tiff]
