# Supplementary material for: Expression Signature as a Biomarker for Prenatal Diagnosis of Trisomy 21
Source: PLoS One. 2013 Sep 16;8(9):e74184. doi: 10.1371/journal.pone.0074184 (PMC3774664; doi:10.1371/journal.pone.0074184)
Supplement: Table S1 — 32 chromosome 21 genes found differentially expressed in trisomy 21 in comparison with euploid amniocyte samples. (DOCX) [file pone.0074184.s003.docx]

Supplementary Table 1

32 chromosome 21 genes found differentially expressed in trisomy 21 in comparison with euploid amniocyte samples

| Agilent Probe ID | RefSeq target ID | Entrez gene ID | Gene symbol | Gene name | Chr | log2FC | P.Value | adj.P.Val |
| --- | --- | --- | --- | --- | --- | --- | --- | --- |
| A_23_P154840 | NM_000454 | 6647 | SOD1 | superoxide dismutase 1, soluble | 21 | 1,2 | 1,1E-10 | 4,5E-06 |
| A_23_P143474 | NM_001697 | 539 | ATP5O | ATP synthase, H+ transporting, mitochondrial F1 complex, O subunit | 21 | 1,2 | 1,0E-08 | 1,4E-04 |
| A_23_P154874 | NM_006052 | 10311 | DSCR3 | Down syndrome critical region gene 3 | 21 | 0,2 | 1,8E-07 | 9,4E-04 |
| A_23_P68717 | NM_003720 | 8624 | PSMG1 | proteasome (prosome, macropain) assembly chaperone 1 | 21 | 1,3 | 3,0E-07 | 1,2E-03 |
| A_23_P211285 | NM_021075 | 4731 | NDUFV3 | NADH dehydrogenase (ubiquinone) flavoprotein 3, 10kDa | 21 | 0,7 | 1,4E-06 | 3,5E-03 |
| A_23_P143484 | NM_153681 | 51227 | PIGP | phosphatidylinositol glycan anchor biosynthesis, class P | 21 | 0,6 | 5,3E-06 | 6,6E-03 |
| A_23_P166219 | NM_002040 | 2551 | GABPA | GA binding protein transcription factor, alpha subunit 60kDa | 21 | 0,1 | 5,6E-06 | 6,6E-03 |
| A_23_P143446 | NM_017446 | 54148 | MRPL39 | mitochondrial ribosomal protein L39 | 21 | 0,9 | 8,2E-06 | 7,4E-03 |
| A_23_P80068 | NM_006806 | 10950 | BTG3 | BTG family, member 3 | 21 | 1,0 | 8,3E-06 | 7,4E-03 |
| A_24_P14595 | NM_133634 | 23275 | POFUT2 | protein O-fucosyltransferase 2 | 21 | 0,8 | 1,2E-05 | 8,9E-03 |
| A_23_P252962 | NM_001001132 | 6453 | ITSN1 | intersectin 1 (SH3 domain protein) | 21 | 0,3 | 1,2E-05 | 8,9E-03 |
| A_23_P500390 | NM_017613 | 29980 | DONSON | downstream neighbor of SON | 21 | 1,3 | 1,6E-05 | 8,9E-03 |
| A_24_P134392 | NM_006948 | 6782 | HSPA13 | heat shock protein 70kDa family, member 13 | 21 | 1,0 | 1,7E-05 | 9,2E-03 |
| A_23_P102890 | NM_032476 | 64968 | MRPS6 | mitochondrial ribosomal protein S6 | 21 | 1,7 | 3,0E-05 | 1,1E-02 |
| A_23_P102937 | NM_006936 | 6612 | SUMO3 | SMT3 suppressor of mif two 3 homolog 3 (S. cerevisiae) | 21 | 0,6 | 5,5E-05 | 1,4E-02 |
| A_24_P34155 | ENST00000358356 | 861 | RUNX1 | runt-related transcription factor 1 | 21 | 0,3 | 6,7E-05 | 1,5E-02 |
| A_23_P211302 | NM_033661 | 10785 | WDR4 | WD repeat domain 4 | 21 | 1,2 | 1,1E-04 | 1,9E-02 |
| A_23_P29046 | NM_001757 | 873 | CBR1 | carbonyl reductase 1 | 21 | 0,7 | 1,2E-04 | 2,0E-02 |
| A_23_P80098 | NM_000819 | 2618 | GART | phosphoribosylglycinamide formyltransferase, phosphoribosylglycinamide synthetase, phosphoribosylaminoimidazole synthetase | 21 | 1,0 | 1,3E-04 | 2,0E-02 |
| A_23_P257911 | NM_001032410 | 10600 | USP16 | ubiquitin specific peptidase 16 | 21 | 1,2 | 1,4E-04 | 2,1E-02 |
| A_23_P356466 | NM_020132 | 56894 | AGPAT3 | 1-acylglycerol-3-phosphate O-acyltransferase 3 | 21 | 0,5 | 2,2E-04 | 2,4E-02 |
| A_23_P57347 | NM_006031 | 5116 | PCNT | pericentrin | 21 | 0,4 | 2,4E-04 | 2,5E-02 |
| A_23_P317683 | NM_003274 | 7109 | TRAPPC10 | trafficking protein particle complex 10 | 21 | 0,5 | 2,4E-04 | 2,5E-02 |
| A_23_P211252 | NM_001001438 | 4047 | LSS | lanosterol synthase (2,3-oxidosqualene-lanosterol cyclase) | 21 | 0,1 | 2,6E-04 | 2,5E-02 |
| A_23_P91487 | NM_016940 | 10069 | RWDD2B | RWD domain containing 2B | 21 | 0,2 | 2,6E-04 | 2,5E-02 |
| A_23_P304991 | NM_000411 | 3141 | HLCS | holocarboxylase synthetase (biotin-(proprionyl-CoA-carboxylase (ATP-hydrolysing)) ligase) | 21 | 0,4 | 2,9E-04 | 2,6E-02 |
| A_23_P6303 | NM_001025204 | 7307 | U2AF1 | U2 small nuclear RNA auxiliary factor 1 | 21 | 0,6 | 3,2E-04 | 2,6E-02 |
| A_23_P29083 | NM_194255 | 6573 | SLC19A1 | solute carrier family 19 (folate transporter), member 1 | 21 | 0,6 | 3,3E-04 | 2,7E-02 |
| A_23_P40415 | NM_007038 | 11096 | ADAMTS5 | ADAM metallopeptidase with thrombospondin type 1 motif, 5 | 21 | -1,8 | 5,6E-04 | 3,4E-02 |
| A_23_P57306 | NM_005441 | 8208 | CHAF1B | chromatin assembly factor 1, subunit B (p60) | 21 | 1,0 | 7,5E-04 | 3,8E-02 |
