# Supplementary material for: Expression Signature as a Biomarker for Prenatal Diagnosis of Trisomy 21
Source: PLoS One. 2013 Sep 16;8(9):e74184. doi: 10.1371/journal.pone.0074184 (PMC3774664; doi:10.1371/journal.pone.0074184)
Supplement: Table S2 — 240 candidate genes for inclusion in the core biomarker gene set, satisfying 2 criteria: attaining false-discovery rate values below 0.05 and 50% reduction or increase in expression in DS amniocytes. (DOCX) [file pone.0074184.s004.docx]

Supplementary Table 2

240 candidate genes for inclusion in the core biomarker gene set, satisfying 2 criterions: attaining false-discovery rate values below 0.05 and 50% reduction or increase in expression in DS amniocytes.

| Agilent Probe ID | RefSeq target ID | Entrez gene ID | Gene symbol | Gene name | Chr | log2FC | P.Value | adj.P.Val |
| --- | --- | --- | --- | --- | --- | --- | --- | --- |
| A_23_P154840 | NM_000454 | 6647 | SOD1 | superoxide dismutase 1, soluble | 21 | 1,2 | 1,1E-10 | 4,5E-06 |
| A_23_P143474 | NM_001697 | 539 | ATP5O | ATP synthase, H+ transporting, mitochondrial F1 complex, O subunit | 21 | 1,2 | 1,0E-08 | 1,4E-04 |
| A_23_P3496 | NM_080861 | 90864 | SPSB3 | splA/ryanodine receptor domain and SOCS box containing 3 | 16 | -1,1 | 1,8E-07 | 9,4E-04 |
| A_23_P68717 | NM_003720 | 8624 | PSMG1 | proteasome (prosome, macropain) assembly chaperone 1 | 21 | 1,3 | 3,0E-07 | 1,2E-03 |
| A_23_P65401 | NM_021914 | 1073 | CFL2 | cofilin 2 (muscle) | 14 | 1,0 | 3,1E-07 | 1,2E-03 |
| A_23_P252642 | NM_152384 | 129880 | BBS5 | Bardet-Biedl syndrome 5 | 2 | -0,9 | 8,3E-07 | 2,1E-03 |
| A_23_P211285 | NM_021075 | 4731 | NDUFV3 | NADH dehydrogenase (ubiquinone) flavoprotein 3, 10kDa | 21 | 0,7 | 1,4E-06 | 3,5E-03 |
| A_23_P408095 | NM_001011546 | 11034 | DSTN | destrin (actin depolymerizing factor) | 20 | 1,3 | 1,6E-06 | 3,5E-03 |
| A_23_P78342 | NM_005570 | 3998 | LMAN1 | lectin, mannose-binding, 1 | 18 | 0,7 | 1,7E-06 | 3,5E-03 |
| A_23_P111745 | NM_031449 | 83637 | ZMIZ2 | zinc finger, MIZ-type containing 2 | 7 | -0,8 | 1,9E-06 | 3,6E-03 |
| A_24_P299318 | NM_182705 | 359845 | FAM101B | family with sequence similarity 101, member B | 17 | 2,1 | 1,9E-06 | 3,6E-03 |
| A_32_P45738 | NM_002629 | 5223 | PGAM1 | phosphoglycerate mutase 1 (brain) | 10 | 0,9 | 2,4E-06 | 4,1E-03 |
| A_32_P209230 | NM_133467 | 163732 | CITED4 | Cbp/p300-interacting transactivator, with Glu/Asp-rich carboxy-terminal domain, 4 | 1 | -1,0 | 3,9E-06 | 6,1E-03 |
| A_23_P128147 | NM_006082 | 10376 | TUBA1B | tubulin, alpha 1b | 12 | 1,7 | 4,9E-06 | 6,6E-03 |
| A_23_P86012 | NM_001017402 | 3914 | LAMB3 | laminin, beta 3 | 1 | -2,5 | 5,8E-06 | 6,6E-03 |
| A_23_P81392 | NM_015238 | 23286 | WWC1 | WW and C2 domain containing 1 | 5 | -1,5 | 6,0E-06 | 6,6E-03 |
| A_23_P204427 | NM_002710 | 5501 | PPP1CC | protein phosphatase 1, catalytic subunit, gamma isozyme | 12 | 1,1 | 6,6E-06 | 6,7E-03 |
| A_24_P334130 | NM_054034 | 2335 | FN1 | fibronectin 1 | 2 | -1,2 | 7,0E-06 | 6,7E-03 |
| A_23_P2661 | NM_015646 | 5908 | RAP1B | RAP1B, member of RAS oncogene family | 12 | 1,0 | 7,5E-06 | 7,0E-03 |
| A_23_P143446 | NM_017446 | 54148 | MRPL39 | mitochondrial ribosomal protein L39 | 21 | 0,9 | 8,2E-06 | 7,4E-03 |
| A_23_P80068 | NM_006806 | 10950 | BTG3 | BTG family, member 3 | 21 | 1,0 | 8,3E-06 | 7,4E-03 |
| A_23_P149529 | NM_002353 | 4070 | TACSTD2 | tumor-associated calcium signal transducer 2 | 1 | -1,6 | 1,0E-05 | 8,6E-03 |
| A_24_P14595 | NM_133634 | 23275 | POFUT2 | protein O-fucosyltransferase 2 | 21 | 0,8 | 1,2E-05 | 8,9E-03 |
| A_32_P222383 | NM_005517 | 3151 | HMGN2 | high mobility group nucleosomal binding domain 2 | 1 | 1,5 | 1,2E-05 | 8,9E-03 |
| A_23_P17593 | NM_001794 | 1002 | CDH4 | cadherin 4, type 1, R-cadherin (retinal) | 20 | -0,8 | 1,4E-05 | 8,9E-03 |
| A_23_P370434 | NM_001212 | 708 | C1QBP | complement component 1, q subcomponent binding protein | 17 | 1,1 | 1,4E-05 | 8,9E-03 |
| A_23_P126970 | NM_006335 | 10440 | TIMM17A | translocase of inner mitochondrial membrane 17 homolog A (yeast) | 1 | 0,7 | 1,5E-05 | 8,9E-03 |
| A_32_P137939 | NM_001101 | 60 | ACTB | actin, beta | 7 | 0,9 | 1,6E-05 | 8,9E-03 |
| A_32_P21993 | NM_003290 | 7171 | TPM4 | tropomyosin 4 | 19 | 0,7 | 1,6E-05 | 8,9E-03 |
| A_23_P500390 | NM_017613 | 29980 | DONSON | downstream neighbor of SON | 21 | 1,3 | 1,6E-05 | 8,9E-03 |
| A_23_P62659 | NM_000310 | 5538 | PPT1 | palmitoyl-protein thioesterase 1 | 1 | 1,0 | 1,6E-05 | 8,9E-03 |
| A_24_P134392 | NM_006948 | 6782 | HSPA13 | heat shock protein 70kDa family, member 13 | 21 | 1,0 | 1,7E-05 | 9,2E-03 |
| A_23_P37484 | NM_014918 | 22856 | CHSY1 | chondroitin sulfate synthase 1 | 15 | 1,0 | 1,9E-05 | 9,7E-03 |
| A_23_P19938 | NM_006854 | 11014 | KDELR2 | KDEL (Lys-Asp-Glu-Leu) endoplasmic reticulum protein retention receptor 2 | 7 | 1,1 | 1,9E-05 | 9,7E-03 |
| A_23_P61886 | AK055659 | 10098 | TSPAN5 | tetraspanin 5 | 4 | 1,0 | 2,0E-05 | 9,7E-03 |
| A_23_P391689 | CR602285 | 1E+08 | C19orf79 | chromosome 19 open reading frame 79 | 19 | -0,8 | 2,0E-05 | 9,7E-03 |
| A_23_P258621 | NM_003729 | 8634 | RTCD1 | RNA terminal phosphate cyclase domain 1 | 1 | 0,7 | 2,0E-05 | 9,7E-03 |
| A_23_P311912 | BC090889 | 113146 | AHNAK2 | AHNAK nucleoprotein 2 | 14 | -0,9 | 2,1E-05 | 9,7E-03 |
| A_32_P25273 | NM_002156 | 3329 | HSPD1 | heat shock 60kDa protein 1 (chaperonin) | 2 | 1,1 | 2,2E-05 | 9,7E-03 |
| A_24_P226008 | NM_007283 | 11343 | MGLL | monoglyceride lipase | 3 | -1,2 | 2,4E-05 | 1,0E-02 |
| A_23_P41280 | NM_001079525 | 10606 | PAICS | phosphoribosylaminoimidazole carboxylase, phosphoribosylaminoimidazole succinocarboxamide synthetase | 4 | 1,0 | 2,4E-05 | 1,0E-02 |
| A_32_P101031 | NM_144586 | 116372 | LYPD1 | LY6/PLAUR domain containing 1 | 2 | 2,8 | 2,5E-05 | 1,0E-02 |
| A_23_P141520 | NM_174893 | 124944 | C17orf49 | chromosome 17 open reading frame 49 | 17 | -0,7 | 2,5E-05 | 1,0E-02 |
| A_23_P431388 | NM_144569 | 90853 | SPOCD1 | SPOC domain containing 1 | 1 | 0,8 | 2,6E-05 | 1,0E-02 |
| A_23_P88589 | NM_021005 | 7026 | NR2F2 | nuclear receptor subfamily 2, group F, member 2 | 15 | 1,6 | 2,7E-05 | 1,1E-02 |
| A_23_P394064 | NM_012232 | 284119 | PTRF | polymerase I and transcript release factor | 17 | 1,0 | 2,9E-05 | 1,1E-02 |
| A_32_P164522 | NM_182620 | 348235 | SKA2 | spindle and kinetochore associated complex subunit 2 | 17 | 0,7 | 3,0E-05 | 1,1E-02 |
| A_23_P150446 | NM_006360 | 10480 | EIF3M | eukaryotic translation initiation factor 3, subunit M | 11 | 0,7 | 3,0E-05 | 1,1E-02 |
| A_23_P102890 | NM_032476 | 64968 | MRPS6 | mitochondrial ribosomal protein S6 | 21 | 1,7 | 3,0E-05 | 1,1E-02 |
| A_23_P90099 | NM_198536 | 374882 | TMEM205 | transmembrane protein 205 | 19 | -0,8 | 3,3E-05 | 1,1E-02 |
| A_23_P64617 | NM_012193 | 8322 | FZD4 | frizzled family receptor 4 | 11 | 0,8 | 3,4E-05 | 1,1E-02 |
| A_23_P102113 | NM_025216 | 80326 | WNT10A | wingless-type MMTV integration site family, member 10A | 2 | -1,6 | 3,5E-05 | 1,1E-02 |
| A_23_P21644 | NM_016245 | 51170 | HSD17B11 | hydroxysteroid (17-beta) dehydrogenase 11 | 4 | 1,1 | 4,3E-05 | 1,3E-02 |
| A_23_P134176 | NM_001024465 | 6648 | SOD2 | superoxide dismutase 2, mitochondrial | 6 | -1,4 | 4,7E-05 | 1,3E-02 |
| A_23_P259272 | NM_018639 | 55884 | WSB2 | WD repeat and SOCS box containing 2 | 12 | 1,3 | 4,8E-05 | 1,3E-02 |
| A_23_P163087 | NM_007361 | 22795 | NID2 | nidogen 2 (osteonidogen) | 14 | 1,1 | 4,8E-05 | 1,3E-02 |
| A_23_P425880 | NM_007118 | 7204 | TRIO | triple functional domain (PTPRF interacting) | 5 | -0,7 | 4,8E-05 | 1,3E-02 |
| A_23_P171296 | NM_002436 | 4354 | MPP1 | membrane protein, palmitoylated 1, 55kDa | X | 0,7 | 4,8E-05 | 1,3E-02 |
| A_23_P111141 | NM_004639 | 7917 | BAG6 | BCL2-associated athanogene 6 | 6 | -0,8 | 5,2E-05 | 1,4E-02 |
| A_32_P231391 | NM_005566 | 3939 | LDHA | lactate dehydrogenase A | 11 | 1,5 | 5,6E-05 | 1,4E-02 |
| A_23_P359111 | NM_001018067 | 26135 | SERBP1 | SERPINE1 mRNA binding protein 1 | 1 | 0,9 | 6,3E-05 | 1,5E-02 |
| A_23_P304450 | NM_005257 | 2627 | GATA6 | GATA binding protein 6 | 18 | 1,2 | 6,4E-05 | 1,5E-02 |
| A_23_P11744 | NM_199163 | 1E+08 | WASH1 | WAS protein family homolog 1 | 9 | -0,8 | 6,6E-05 | 1,5E-02 |
| A_23_P119362 | NM_001425 | 2014 | EMP3 | epithelial membrane protein 3 | 19 | -0,8 | 7,0E-05 | 1,6E-02 |
| A_24_P125690 | NM_023937 | 64981 | MRPL34 | mitochondrial ribosomal protein L34 | 19 | -0,7 | 8,1E-05 | 1,7E-02 |
| A_23_P33196 | NM_000393 | 1290 | COL5A2 | collagen, type V, alpha 2 | 2 | 1,6 | 8,3E-05 | 1,7E-02 |
| A_24_P335092 | NM_000331 | 6288 | SAA1 | serum amyloid A1 | 11 | -2,0 | 8,4E-05 | 1,7E-02 |
| A_32_P12639 | NM_002816 | 5718 | PSMD12 | proteasome (prosome, macropain) 26S subunit, non-ATPase, 12 | 17 | 0,7 | 8,5E-05 | 1,7E-02 |
| A_23_P58328 | NM_007193 | 11199 | ANXA10 | annexin A10 | 4 | 0,8 | 8,6E-05 | 1,7E-02 |
| A_23_P209636 | NM_006449 | 10602 | CDC42EP3 | CDC42 effector protein (Rho GTPase binding) 3 | 2 | 1,1 | 8,9E-05 | 1,7E-02 |
| A_23_P423419 | NM_001005266 | 283651 | HMGN2P46 | high mobility group nucleosomal binding domain 2 pseudogene 46 | 15 | 1,8 | 9,3E-05 | 1,8E-02 |
| A_23_P49708 | NM_002087 | 2896 | GRN | granulin | 17 | -1,2 | 9,3E-05 | 1,8E-02 |
| A_24_P88763 | NM_032603 | 84695 | LOXL3 | lysyl oxidase-like 3 | 2 | 1,3 | 9,5E-05 | 1,8E-02 |
| A_24_P300777 | NM_001109 | 101 | ADAM8 | ADAM metallopeptidase domain 8 | 10 | -1,3 | 9,8E-05 | 1,8E-02 |
| A_23_P400465 | NM_138408 | 112495 | GTF3C6 | general transcription factor IIIC, polypeptide 6, alpha 35kDa | 6 | 1,0 | 1,0E-04 | 1,8E-02 |
| A_23_P19036 | NM_006058 | 10318 | TNIP1 | TNFAIP3 interacting protein 1 | 5 | -0,8 | 1,1E-04 | 1,9E-02 |
| A_24_P58337 | NM_002032 | 2495 | FTH1 | ferritin, heavy polypeptide 1 | 11 | -1,1 | 1,1E-04 | 1,9E-02 |
| A_23_P211302 | NM_033661 | 10785 | WDR4 | WD repeat domain 4 | 21 | 1,2 | 1,1E-04 | 1,9E-02 |
| A_23_P113005 | NM_004428 | 1942 | EFNA1 | ephrin-A1 | 1 | -0,8 | 1,1E-04 | 1,9E-02 |
| A_23_P207911 | NM_016113 | 51393 | TRPV2 | transient receptor potential cation channel, subfamily V, member 2 | 17 | 1,2 | 1,2E-04 | 1,9E-02 |
| A_24_P344087 | NM_001048205 | 9985 | REC8 | REC8 homolog (yeast) | 14 | -0,8 | 1,2E-04 | 2,0E-02 |
| A_23_P41645 | NM_012081 | 22936 | ELL2 | elongation factor, RNA polymerase II, 2 | 5 | 1,3 | 1,2E-04 | 2,0E-02 |
| A_23_P1782 | NM_002231 | 3732 | CD82 | CD82 molecule | 11 | -1,0 | 1,2E-04 | 2,0E-02 |
| A_23_P24594 | NM_006597 | 3312 | HSPA8 | heat shock 70kDa protein 8 | 11 | 0,8 | 1,2E-04 | 2,0E-02 |
| A_23_P29046 | NM_001757 | 873 | CBR1 | carbonyl reductase 1 | 21 | 0,7 | 1,2E-04 | 2,0E-02 |
| A_23_P74034 | NM_014412 | 27101 | CACYBP | calcyclin binding protein | 1 | 1,0 | 1,3E-04 | 2,0E-02 |
| A_23_P157215 | NM_014038 | 28969 | BZW2 | basic leucine zipper and W2 domains 2 | 7 | 1,2 | 1,3E-04 | 2,0E-02 |
| A_23_P133408 | NM_000758 | 1437 | CSF2 | colony stimulating factor 2 (granulocyte-macrophage) | 5 | -1,4 | 1,3E-04 | 2,0E-02 |
| A_23_P80098 | NM_000819 | 2618 | GART | phosphoribosylglycinamide formyltransferase, phosphoribosylglycinamide synthetase, phosphoribosylaminoimidazole synthetase | 21 | 1,0 | 1,3E-04 | 2,0E-02 |
| A_23_P372234 | NM_001218 | 771 | CA12 | carbonic anhydrase XII | 15 | 1,0 | 1,3E-04 | 2,0E-02 |
| A_23_P57089 | NM_020182 | 56937 | PMEPA1 | prostate transmembrane protein, androgen induced 1 | 20 | -1,3 | 1,4E-04 | 2,1E-02 |
| A_23_P257911 | NM_001032410 | 10600 | USP16 | ubiquitin specific peptidase 16 | 21 | 1,2 | 1,4E-04 | 2,1E-02 |
| A_23_P250122 | NM_020223 | 56975 | FAM20C | family with sequence similarity 20, member C | 7 | -1,0 | 1,5E-04 | 2,1E-02 |
| A_32_P11499 | NM_003348 | 7334 | UBE2N | ubiquitin-conjugating enzyme E2N | 12 | 1,0 | 1,5E-04 | 2,1E-02 |
| A_23_P17134 | NM_002371 | 4118 | MAL | mal, T-cell differentiation protein | 2 | -2,6 | 1,5E-04 | 2,1E-02 |
| A_23_P367405 | NM_000281 | 5092 | PCBD1 | pterin-4 alpha-carbinolamine dehydratase/dimerization cofactor of hepatocyte nuclear factor 1 alpha | 10 | -0,7 | 1,5E-04 | 2,1E-02 |
| A_23_P209740 | NM_002807 | 5707 | PSMD1 | proteasome (prosome, macropain) 26S subunit, non-ATPase, 1 | 2 | 0,7 | 1,5E-04 | 2,1E-02 |
| A_23_P37727 | NM_002996 | 6376 | CX3CL1 | chemokine (C-X3-C motif) ligand 1 | 16 | -1,8 | 1,5E-04 | 2,2E-02 |
| A_23_P153586 | NM_006003 | 7386 | UQCRFS1 | ubiquinol-cytochrome c reductase, Rieske iron-sulfur polypeptide 1 | 19 | 0,8 | 1,5E-04 | 2,2E-02 |
| A_23_P119627 | NM_015965 | 51079 | NDUFA13 | NADH dehydrogenase (ubiquinone) 1 alpha subcomplex, 13 | 19 | -0,9 | 1,6E-04 | 2,2E-02 |
| A_23_P68007 | NM_001679 | 483 | ATP1B3 | ATPase, Na+/K+ transporting, beta 3 polypeptide | 3 | 0,7 | 1,6E-04 | 2,2E-02 |
| A_23_P12911 | NM_022338 | 53838 | C11orf24 | chromosome 11 open reading frame 24 | 11 | 0,8 | 1,6E-04 | 2,2E-02 |
| A_23_P300600 | NM_021076 | 4744 | NEFH | neurofilament, heavy polypeptide | 22 | 0,8 | 1,6E-04 | 2,2E-02 |
| A_24_P354689 | NM_004598 | 6695 | SPOCK1 | sparc/osteonectin, cwcv and kazal-like domains proteoglycan (testican) 1 | 5 | 0,9 | 1,6E-04 | 2,2E-02 |
| A_23_P217901 | ENST00000368025 | 1E+08 | TSTD1 | thiosulfate sulfurtransferase (rhodanese)-like domain containing 1 | 1 | -0,8 | 1,6E-04 | 2,2E-02 |
| A_23_P83939 | NM_032796 | 94056 | SYAP1 | synapse associated protein 1 | X | 0,7 | 1,7E-04 | 2,2E-02 |
| A_32_P40288 | NM_052913 | 114801 | TMEM200A | transmembrane protein 200A | 6 | 1,1 | 1,7E-04 | 2,2E-02 |
| A_24_P931443 | NM_003485 | 8111 | GPR68 | G protein-coupled receptor 68 | 14 | -0,7 | 1,7E-04 | 2,2E-02 |
| A_23_P201711 | NM_014624 | 6277 | S100A6 | S100 calcium binding protein A6 | 1 | -0,8 | 1,7E-04 | 2,2E-02 |
| A_23_P42695 | NM_024051 | 79017 | GGCT | gamma-glutamylcyclotransferase | 7 | 0,8 | 1,8E-04 | 2,2E-02 |
| A_24_P188941 | NM_002520 | 4869 | NPM1 | nucleophosmin (nucleolar phosphoprotein B23, numatrin) | 5 | 1,0 | 1,8E-04 | 2,2E-02 |
| A_24_P215765 | NM_024490 | 57194 | ATP10A | ATPase, class V, type 10A | 15 | 0,8 | 1,8E-04 | 2,2E-02 |
| A_32_P60065 | NM_004101 | 2151 | F2RL2 | coagulation factor II (thrombin) receptor-like 2 | 5 | 1,8 | 1,8E-04 | 2,3E-02 |
| A_23_P52761 | NM_002423 | 4316 | MMP7 | matrix metallopeptidase 7 (matrilysin, uterine) | 11 | -3,3 | 1,9E-04 | 2,3E-02 |
| A_23_P82412 | NM_001039575 | 55695 | NSUN5 | NOP2/Sun domain family, member 5 | 7 | -0,7 | 1,9E-04 | 2,3E-02 |
| A_32_P171313 | CR603437 | 59345 | GNB4 | guanine nucleotide binding protein (G protein), beta polypeptide 4 | 3 | 1,0 | 2,0E-04 | 2,4E-02 |
| A_23_P218086 | AB032995 | 53373 | TPCN1 | two pore segment channel 1 | 12 | -1,1 | 2,0E-04 | 2,4E-02 |
| A_23_P24345 | NM_152264 | 91252 | SLC39A13 | solute carrier family 39 (zinc transporter), member 13 | 11 | -0,7 | 2,0E-04 | 2,4E-02 |
| A_23_P27613 | U60266 | 4125 | MAN2B1 | mannosidase, alpha, class 2B, member 1 | 19 | -0,7 | 2,1E-04 | 2,4E-02 |
| A_23_P31315 | NM_016587 | 11335 | CBX3 | chromobox homolog 3 | 7 | 0,8 | 2,1E-04 | 2,4E-02 |
| A_23_P105392 | NM_006431 | 10576 | CCT2 | chaperonin containing TCP1, subunit 2 (beta) | 12 | 0,8 | 2,2E-04 | 2,4E-02 |
| A_23_P91081 | NM_002354 | 4072 | EPCAM | epithelial cell adhesion molecule | 2 | -1,4 | 2,2E-04 | 2,4E-02 |
| A_23_P58353 | NM_031370 | 3184 | HNRNPD | heterogeneous nuclear ribonucleoprotein D (AU-rich element RNA binding protein 1, 37kDa) | 4 | 0,9 | 2,2E-04 | 2,4E-02 |
| A_24_P69654 | NM_001300 | 1316 | KLF6 | Kruppel-like factor 6 | 10 | -1,2 | 2,2E-04 | 2,4E-02 |
| A_24_P296568 | NM_006807 | 10951 | CBX1 | chromobox homolog 1 | 17 | 1,0 | 2,3E-04 | 2,5E-02 |
| A_23_P321630 | NM_020810 | 57570 | TRMT5 | TRM5 tRNA methyltransferase 5 homolog (S. cerevisiae) | 14 | 1,4 | 2,3E-04 | 2,5E-02 |
| A_32_P147078 | NM_021097 | 6546 | SLC8A1 | solute carrier family 8 (sodium/calcium exchanger), member 1 | 2 | 1,2 | 2,4E-04 | 2,5E-02 |
| A_24_P193011 | NM_053056 | 595 | CCND1 | cyclin D1 | 11 | -0,9 | 2,5E-04 | 2,5E-02 |
| A_23_P102420 | NM_006430 | 10575 | CCT4 | chaperonin containing TCP1, subunit 4 (delta) | 2 | 0,8 | 2,5E-04 | 2,5E-02 |
| A_24_P257416 | NM_002089 | 2920 | CXCL2 | chemokine (C-X-C motif) ligand 2 | 4 | -1,2 | 2,7E-04 | 2,5E-02 |
| A_32_P153195 | ENST00000299694 | 146227 | BEAN1 | brain expressed, associated with NEDD4, 1 | 16 | -1,1 | 2,7E-04 | 2,5E-02 |
| A_24_P185854 | NM_004010 | 1756 | DMD | dystrophin | X | 0,9 | 2,7E-04 | 2,5E-02 |
| A_32_P117354 | NM_014988 | 22998 | LIMCH1 | LIM and calponin homology domains 1 | 4 | -0,9 | 2,7E-04 | 2,5E-02 |
| A_24_P111106 | NM_000800 | 2246 | FGF1 | fibroblast growth factor 1 (acidic) | 5 | 0,8 | 2,7E-04 | 2,5E-02 |
| A_23_P114232 | NM_006406 | 10549 | PRDX4 | peroxiredoxin 4 | X | 0,7 | 2,8E-04 | 2,6E-02 |
| A_23_P82169 | NM_003107 | 6659 | SOX4 | SRY (sex determining region Y)-box 4 | 6 | -1,2 | 2,8E-04 | 2,6E-02 |
| A_23_P501822 | NM_002230 | 3728 | JUP | junction plakoglobin | 17 | -1,1 | 2,8E-04 | 2,6E-02 |
| A_23_P164284 | NM_001307 | 1366 | CLDN7 | claudin 7 | 17 | -0,7 | 2,8E-04 | 2,6E-02 |
| A_23_P25150 | NM_006897 | 3225 | HOXC9 | homeobox C9 | 12 | 1,0 | 2,8E-04 | 2,6E-02 |
| A_23_P43566 | NM_014222 | 4702 | NDUFA8 | NADH dehydrogenase (ubiquinone) 1 alpha subcomplex, 8, 19kDa | 9 | 0,7 | 2,9E-04 | 2,6E-02 |
| A_24_P56252 | AF086032 | 4659 | PPP1R12A | protein phosphatase 1, regulatory (inhibitor) subunit 12A | 12 | 0,7 | 2,9E-04 | 2,6E-02 |
| A_32_P47643 | CR601458 | 642273 | FAM110C | family with sequence similarity 110, member C | 2 | -0,8 | 2,9E-04 | 2,6E-02 |
| A_23_P200737 | NM_005613 | 5999 | RGS4 | regulator of G-protein signaling 4 | 1 | 1,7 | 2,9E-04 | 2,6E-02 |
| A_24_P350124 | NM_020954 | 57674 | RNF213 | ring finger protein 213 | 17 | -0,7 | 3,0E-04 | 2,6E-02 |
| A_23_P170733 | NM_058172 | 118429 | ANTXR2 | anthrax toxin receptor 2 | 4 | 1,3 | 3,0E-04 | 2,6E-02 |
| A_23_P140035 | NM_007187 | 11193 | WBP4 | WW domain binding protein 4 (formin binding protein 21) | 13 | 0,7 | 3,0E-04 | 2,6E-02 |
| A_23_P153320 | NM_000201 | 3383 | ICAM1 | intercellular adhesion molecule 1 | 19 | -0,8 | 3,1E-04 | 2,6E-02 |
| A_23_P254271 | NM_032525 | 84617 | TUBB6 | tubulin, beta 6 | 18 | 1,1 | 3,1E-04 | 2,6E-02 |
| A_23_P32444 | NM_032348 | 54587 | MXRA8 | matrix-remodelling associated 8 | 1 | 1,2 | 3,1E-04 | 2,6E-02 |
| A_23_P376488 | NM_000594 | 7124 | TNF | tumor necrosis factor | 6 | -1,5 | 3,1E-04 | 2,6E-02 |
| A_23_P88201 | NM_017917 | 55012 | PPP2R3C | protein phosphatase 2, regulatory subunit B'', gamma | 14 | 0,7 | 3,2E-04 | 2,6E-02 |
| A_23_P35349 | NM_021738 | 6840 | SVIL | supervillin | 10 | -0,8 | 3,3E-04 | 2,6E-02 |
| A_23_P157580 | NM_005625 | 6386 | SDCBP | syndecan binding protein (syntenin) | 8 | -0,7 | 3,3E-04 | 2,7E-02 |
| A_23_P75028 | NM_001001330 | 221035 | REEP3 | receptor accessory protein 3 | 10 | 0,7 | 3,3E-04 | 2,7E-02 |
| A_23_P154605 | NM_018837 | 55959 | SULF2 | sulfatase 2 | 20 | -1,1 | 3,4E-04 | 2,7E-02 |
| A_23_P41777 | NM_024715 | 79770 | TXNDC15 | thioredoxin domain containing 15 | 5 | 0,8 | 3,4E-04 | 2,7E-02 |
| A_23_P144916 | NM_005110 | 9945 | GFPT2 | glutamine-fructose-6-phosphate transaminase 2 | 5 | 0,7 | 3,4E-04 | 2,7E-02 |
| A_23_P152305 | NM_001797 | 1009 | CDH11 | cadherin 11, type 2, OB-cadherin (osteoblast) | 16 | 1,8 | 3,4E-04 | 2,7E-02 |
| A_23_P73747 | NM_014782 | 9823 | ARMCX2 | armadillo repeat containing, X-linked 2 | X | 0,8 | 3,5E-04 | 2,7E-02 |
| A_23_P28075 | NM_001039848 | 2879 | GPX4 | glutathione peroxidase 4 (phospholipid hydroperoxidase) | 19 | -1,0 | 3,8E-04 | 2,9E-02 |
| A_23_P394304 | NM_005764 | 10158 | PDZK1IP1 | PDZK1 interacting protein 1 | 1 | -1,1 | 3,9E-04 | 2,9E-02 |
| A_32_P234935 | NM_007375 | 23435 | TARDBP | TAR DNA binding protein | 1 | 0,9 | 4,0E-04 | 2,9E-02 |
| A_24_P347488 | NM_002802 | 5700 | PSMC1 | proteasome (prosome, macropain) 26S subunit, ATPase, 1 | 14 | 0,7 | 4,2E-04 | 3,0E-02 |
| A_23_P93348 | NM_002341 | 4050 | LTB | lymphotoxin beta (TNF superfamily, member 3) | 6 | -2,1 | 4,2E-04 | 3,0E-02 |
| A_23_P57784 | NM_021101 | 9076 | CLDN1 | claudin 1 | 3 | -1,4 | 4,3E-04 | 3,0E-02 |
| A_23_P126474 | NM_003145 | 6746 | SSR2 | signal sequence receptor, beta (translocon-associated protein beta) | 1 | 0,8 | 4,4E-04 | 3,1E-02 |
| A_23_P41021 | NM_007184 | 11188 | NISCH | nischarin | 3 | -0,8 | 4,4E-04 | 3,1E-02 |
| A_24_P148043 | NM_014864 | 9917 | FAM20B | family with sequence similarity 20, member B | 1 | 0,7 | 4,5E-04 | 3,1E-02 |
| A_23_P201386 | NM_012137 | 23576 | DDAH1 | dimethylarginine dimethylaminohydrolase 1 | 1 | 0,9 | 4,6E-04 | 3,2E-02 |
| A_23_P256542 | NM_014367 | 26355 | FAM162A | family with sequence similarity 162, member A | 3 | 0,9 | 4,6E-04 | 3,2E-02 |
| A_24_P376707 | NM_004494 | 3068 | HDGF | hepatoma-derived growth factor | 1 | -0,8 | 4,6E-04 | 3,2E-02 |
| A_24_P278747 | NM_001759 | 894 | CCND2 | cyclin D2 | 12 | -0,7 | 4,6E-04 | 3,2E-02 |
| A_23_P390044 | NM_002359 | 4097 | MAFG | v-maf musculoaponeurotic fibrosarcoma oncogene homolog G (avian) | 17 | -0,7 | 4,8E-04 | 3,2E-02 |
| A_23_P60248 | NM_003329 | 7295 | TXN | thioredoxin | 9 | 0,9 | 4,8E-04 | 3,2E-02 |
| A_23_P91590 | NM_002882 | 5902 | RANBP1 | RAN binding protein 1 | 22 | 1,4 | 4,9E-04 | 3,2E-02 |
| A_23_P67708 | NM_003200 | 6929 | TCF3 | transcription factor 3 (E2A immunoglobulin enhancer binding factors E12/E47) | 19 | 0,9 | 4,9E-04 | 3,2E-02 |
| A_23_P14482 | NM_016039 | 51637 | C14orf166 | chromosome 14 open reading frame 166 | 14 | 0,7 | 5,0E-04 | 3,2E-02 |
| A_23_P151426 | NM_002015 | 2308 | FOXO1 | forkhead box O1 | 13 | -0,7 | 5,0E-04 | 3,2E-02 |
| A_23_P252721 | NM_182643 | 10395 | DLC1 | deleted in liver cancer 1 | 8 | 1,0 | 5,1E-04 | 3,3E-02 |
| A_23_P415510 | NM_005558 | 3898 | LAD1 | ladinin 1 | 1 | -0,9 | 5,2E-04 | 3,3E-02 |
| A_23_P329353 | NM_015463 | 25927 | CNRIP1 | cannabinoid receptor interacting protein 1 | 2 | 0,7 | 5,4E-04 | 3,3E-02 |
| A_23_P382775 | NM_014417 | 27113 | BBC3 | BCL2 binding component 3 | 19 | -1,1 | 5,5E-04 | 3,4E-02 |
| A_23_P138635 | NM_004052 | 664 | BNIP3 | BCL2/adenovirus E1B 19kDa interacting protein 3 | 10 | 1,3 | 5,5E-04 | 3,4E-02 |
| A_23_P144378 | NM_032217 | 26057 | ANKRD17 | ankyrin repeat domain 17 | 4 | -0,7 | 5,6E-04 | 3,4E-02 |
| A_23_P258190 | NM_001628 | 231 | AKR1B1 | aldo-keto reductase family 1, member B1 (aldose reductase) | 7 | -1,9 | 5,6E-04 | 3,4E-02 |
| A_23_P40415 | NM_007038 | 11096 | ADAMTS5 | ADAM metallopeptidase with thrombospondin type 1 motif, 5 | 21 | -1,8 | 5,6E-04 | 3,4E-02 |
| A_23_P357811 | NM_021038 | 4154 | MBNL1 | muscleblind-like (Drosophila) | 3 | 0,8 | 5,7E-04 | 3,4E-02 |
| A_23_P1962 | NM_004585 | 5920 | RARRES3 | retinoic acid receptor responder (tazarotene induced) 3 | 11 | -0,8 | 5,7E-04 | 3,4E-02 |
| A_23_P1043 | NM_018265 | 55765 | C1orf106 | chromosome 1 open reading frame 106 | 1 | -0,8 | 5,7E-04 | 3,4E-02 |
| A_23_P163506 | NM_000101 | 1535 | CYBA | cytochrome b-245, alpha polypeptide | 16 | -1,1 | 5,8E-04 | 3,4E-02 |
| A_23_P7727 | NM_001884 | 1404 | HAPLN1 | hyaluronan and proteoglycan link protein 1 | 5 | 1,6 | 5,8E-04 | 3,4E-02 |
| A_23_P112251 | NM_001017998 | 2790 | GNG10 | guanine nucleotide binding protein (G protein), gamma 10 | 9 | 0,8 | 5,8E-04 | 3,4E-02 |
| A_23_P26024 | NM_032413 | 84419 | C15orf48 | chromosome 15 open reading frame 48 | 15 | -1,7 | 6,0E-04 | 3,5E-02 |
| A_23_P109269 | NM_005560 | 3911 | LAMA5 | laminin, alpha 5 | 20 | -0,7 | 6,1E-04 | 3,5E-02 |
| A_23_P61688 | NM_006598 | 10723 | SLC12A7 | solute carrier family 12 (potassium/chloride transporters), member 7 | 5 | -1,1 | 6,1E-04 | 3,5E-02 |
| A_23_P142724 | NM_000998 | 6168 | RPL37A | ribosomal protein L37a | 2 | -1,8 | 6,2E-04 | 3,5E-02 |
| A_23_P132936 | NM_021928 | 60559 | SPCS3 | signal peptidase complex subunit 3 homolog (S. cerevisiae) | 4 | 1,0 | 6,2E-04 | 3,5E-02 |
| A_23_P213620 | NM_004576 | 5521 | PPP2R2B | protein phosphatase 2, regulatory subunit B, beta | 5 | 1,1 | 6,3E-04 | 3,6E-02 |
| A_23_P129614 | NM_007006 | 11051 | NUDT21 | nudix (nucleoside diphosphate linked moiety X)-type motif 21 | 16 | 0,8 | 6,5E-04 | 3,6E-02 |
| A_23_P128554 | NM_032565 | 84650 | EBPL | emopamil binding protein-like | 13 | 0,9 | 6,6E-04 | 3,6E-02 |
| A_23_P60130 | NM_052886 | 114569 | MAL2 | mal, T-cell differentiation protein 2 (gene/pseudogene) | 8 | -1,2 | 6,6E-04 | 3,6E-02 |
| A_23_P52986 | NM_152718 | 220001 | VWCE | von Willebrand factor C and EGF domains | 11 | -0,8 | 6,6E-04 | 3,6E-02 |
| A_24_P339201 | NM_024042 | 79006 | METRN | meteorin, glial cell differentiation regulator | 16 | -0,9 | 6,8E-04 | 3,7E-02 |
| A_23_P127533 | NM_032299 | 84259 | DCUN1D5 | DCN1, defective in cullin neddylation 1, domain containing 5 (S. cerevisiae) | 11 | 0,9 | 7,0E-04 | 3,7E-02 |
| A_32_P196263 | NM_182920 | 56999 | ADAMTS9 | ADAM metallopeptidase with thrombospondin type 1 motif, 9 | 3 | -0,7 | 7,0E-04 | 3,7E-02 |
| A_23_P90612 | NM_005915 | 4175 | MCM6 | minichromosome maintenance complex component 6 | 2 | 1,0 | 7,2E-04 | 3,8E-02 |
| A_32_P75284 | NM_032116 | 84056 | KATNAL1 | katanin p60 subunit A-like 1 | 13 | 0,9 | 7,3E-04 | 3,8E-02 |
| A_23_P306203 | NM_030754 | 6289 | SAA2 | serum amyloid A2 | 11 | -1,2 | 7,3E-04 | 3,8E-02 |
| A_23_P114670 | NM_014448 | 27237 | ARHGEF16 | Rho guanine nucleotide exchange factor (GEF) 16 | 1 | -0,9 | 7,5E-04 | 3,8E-02 |
| A_23_P101407 | NM_000064 | 718 | C3 | complement component 3 | 19 | -1,5 | 7,5E-04 | 3,8E-02 |
| A_23_P57306 | NM_005441 | 8208 | CHAF1B | chromatin assembly factor 1, subunit B (p60) | 21 | 1,0 | 7,5E-04 | 3,8E-02 |
| A_23_P122852 | NM_003078 | 6604 | SMARCD3 | SWI/SNF related, matrix associated, actin dependent regulator of chromatin, subfamily d, member 3 | 7 | 0,7 | 7,5E-04 | 3,8E-02 |
| A_24_P301146 | NM_005358 | 4008 | LMO7 | LIM domain 7 | 13 | 1,4 | 7,6E-04 | 3,8E-02 |
| A_23_P29630 | NM_014041 | 28972 | SPCS1 | signal peptidase complex subunit 1 homolog (S. cerevisiae) | 3 | -0,7 | 7,8E-04 | 3,9E-02 |
| A_23_P413761 | NM_003017 | 6428 | SRSF3 | serine/arginine-rich splicing factor 3 | 6 | 0,8 | 7,8E-04 | 3,9E-02 |
| A_23_P144796 | NM_003687 | 8572 | PDLIM4 | PDZ and LIM domain 4 | 5 | -1,2 | 7,9E-04 | 3,9E-02 |
| A_24_P116535 | NM_002428 | 4324 | MMP15 | matrix metallopeptidase 15 (membrane-inserted) | 16 | -0,8 | 8,1E-04 | 3,9E-02 |
| A_23_P254254 | NM_000199 | 6448 | SGSH | N-sulfoglucosamine sulfohydrolase | 17 | -0,8 | 8,1E-04 | 4,0E-02 |
| A_23_P206359 | NM_004360 | 999 | CDH1 | cadherin 1, type 1, E-cadherin (epithelial) | 16 | -0,8 | 8,1E-04 | 4,0E-02 |
| A_23_P107421 | NM_003258 | 7083 | TK1 | thymidine kinase 1, soluble | 17 | 2,3 | 8,2E-04 | 4,0E-02 |
| A_23_P127175 | NM_020150 | 56681 | SAR1A | SAR1 homolog A (S. cerevisiae) | 10 | 0,9 | 8,3E-04 | 4,0E-02 |
| A_23_P143127 | NM_019063 | 27436 | EML4 | echinoderm microtubule associated protein like 4 | 2 | 0,7 | 8,4E-04 | 4,0E-02 |
| A_24_P410678 | NM_002227 | 3716 | JAK1 | Janus kinase 1 | 1 | -0,9 | 8,5E-04 | 4,1E-02 |
| A_23_P983 | NM_004905 | 9588 | PRDX6 | peroxiredoxin 6 | 1 | 0,7 | 8,5E-04 | 4,1E-02 |
| A_23_P71037 | NM_000600 | 3569 | IL6 | interleukin 6 (interferon, beta 2) | 7 | -2,1 | 8,7E-04 | 4,1E-02 |
| A_23_P122197 | NM_031966 | 891 | CCNB1 | cyclin B1 | 5 | 1,4 | 8,7E-04 | 4,1E-02 |
| A_23_P150092 | NM_012247 | 22929 | SEPHS1 | selenophosphate synthetase 1 | 10 | 0,8 | 8,8E-04 | 4,1E-02 |
| A_23_P118815 | NM_001012271 | 332 | BIRC5 | baculoviral IAP repeat containing 5 | 17 | 2,0 | 9,0E-04 | 4,1E-02 |
| A_23_P44724 | NM_001321 | 1466 | CSRP2 | cysteine and glycine-rich protein 2 | 12 | 1,1 | 9,1E-04 | 4,2E-02 |
| A_23_P203900 | NM_005505 | 949 | SCARB1 | scavenger receptor class B, member 1 | 12 | 0,9 | 9,1E-04 | 4,2E-02 |
| A_23_P150286 | NM_148976 | 5682 | PSMA1 | proteasome (prosome, macropain) subunit, alpha type, 1 | 11 | 0,8 | 9,2E-04 | 4,2E-02 |
| A_24_P366082 | ENST00000252134 | 57553 | MICAL3 | microtubule associated monoxygenase, calponin and LIM domain containing 3 | 22 | -0,7 | 9,2E-04 | 4,2E-02 |
| A_23_P50096 | NM_001071 | 7298 | TYMS | thymidylate synthetase | 18 | 1,6 | 9,3E-04 | 4,2E-02 |
| A_23_P69188 | NM_206831 | 285381 | DPH3 | DPH3, KTI11 homolog (S. cerevisiae) | 3 | 0,7 | 9,4E-04 | 4,2E-02 |
| A_23_P43164 | NM_015170 | 23213 | SULF1 | sulfatase 1 | 8 | 2,1 | 9,6E-04 | 4,3E-02 |
| A_23_P388433 | CR597270 | 401152 | C4orf3 | chromosome 4 open reading frame 3 | 4 | 0,8 | 9,8E-04 | 4,3E-02 |
| A_23_P164210 | NM_032258 | 84218 | TBC1D3F | TBC1 domain family, member 3F | 17 | -0,7 | 1,0E-03 | 4,3E-02 |
| A_23_P74349 | NM_145697 | 83540 | NUF2 | NUF2, NDC80 kinetochore complex component, homolog (S. cerevisiae) | 1 | 0,7 | 1,0E-03 | 4,4E-02 |
| A_23_P132973 | NM_001042616 | 84992 | PIGY | phosphatidylinositol glycan anchor biosynthesis, class Y | 4 | 0,7 | 1,0E-03 | 4,4E-02 |
| A_23_P253412 | NM_019051 | 54534 | MRPL50 | mitochondrial ribosomal protein L50 | 9 | 0,8 | 1,0E-03 | 4,4E-02 |
