# Supplementary material for: Expression Signature as a Biomarker for Prenatal Diagnosis of Trisomy 21
Source: PLoS One. 2013 Sep 16;8(9):e74184. doi: 10.1371/journal.pone.0074184 (PMC3774664; doi:10.1371/journal.pone.0074184)
Supplement: Table S3 — 932 non-chromosome 21 genes found differentially expressed in trisomy 21 in comparison with euploid amniocyte samples. (DOCX) [file pone.0074184.s005.docx]

Supplementary Table 3

932 non-chromosome 21 genes found differentially expressed in trisomy 21 in comparison with euploid amniocyte samples

| Agilent Probe ID | RefSeq target ID | Entrez gene ID | Gene symbol | Gene name | Chr | log2FC | P.Value | adj.P.Val |
| --- | --- | --- | --- | --- | --- | --- | --- | --- |
| A_23_P3496 | NM_080861 | 90864 | SPSB3 | splA/ryanodine receptor domain and SOCS box containing 3 | 16 | -1,1 | 1,8E-07 | 9,4E-04 |
| A_24_P181510 | NM_014699 | 9726 | ZNF646 | zinc finger protein 646 | 16 | -0,1 | 1,8E-07 | 9,4E-04 |
| A_24_P232049 | NM_014504 | 27342 | RABGEF1 | RAB guanine nucleotide exchange factor (GEF) 1 | 7 | 0,3 | 2,7E-07 | 1,2E-03 |
| A_23_P65401 | NM_021914 | 1073 | CFL2 | cofilin 2 (muscle) | 14 | 1,0 | 3,1E-07 | 1,2E-03 |
| A_23_P48070 | NM_016162 | 51147 | ING4 | inhibitor of growth family, member 4 | 12 | -0,3 | 3,6E-07 | 1,2E-03 |
| A_24_P35891 | NM_016423 | 51222 | ZNF219 | zinc finger protein 219 | 14 | -0,6 | 4,5E-07 | 1,4E-03 |
| A_23_P41267 | BC010526 | 401127 | LOC401127 | WD repeat domain 5 pseudogene | 4 | 0,1 | 7,2E-07 | 2,0E-03 |
| A_23_P252642 | NM_152384 | 129880 | BBS5 | Bardet-Biedl syndrome 5 | 2 | -0,9 | 8,3E-07 | 2,1E-03 |
| A_23_P408095 | NM_001011546 | 11034 | DSTN | destrin (actin depolymerizing factor) | 20 | 1,3 | 1,6E-06 | 3,5E-03 |
| A_23_P78342 | NM_005570 | 3998 | LMAN1 | lectin, mannose-binding, 1 | 18 | 0,7 | 1,7E-06 | 3,5E-03 |
| A_32_P132206 | NM_017414 | 11274 | USP18 | ubiquitin specific peptidase 18 | 22 | 0,4 | 1,7E-06 | 3,5E-03 |
| A_23_P111745 | NM_031449 | 83637 | ZMIZ2 | zinc finger, MIZ-type containing 2 | 7 | -0,8 | 1,9E-06 | 3,6E-03 |
| A_24_P299318 | NM_182705 | 359845 | FAM101B | family with sequence similarity 101, member B | 17 | 2,1 | 1,9E-06 | 3,6E-03 |
| A_32_P45738 | NM_002629 | 5223 | PGAM1 | phosphoglycerate mutase 1 (brain) | 10 | 0,9 | 2,4E-06 | 4,1E-03 |
| A_24_P476386 | THC2662279 | 3831 | KLC1 | kinesin light chain 1 | 14 | -0,6 | 3,8E-06 | 6,1E-03 |
| A_32_P209230 | NM_133467 | 163732 | CITED4 | Cbp/p300-interacting transactivator, with Glu/Asp-rich carboxy-terminal domain, 4 | 1 | -1,0 | 3,9E-06 | 6,1E-03 |
| A_23_P325625 | NM_173690 | 286205 | SCAI | suppressor of cancer cell invasion | 9 | 0,1 | 4,2E-06 | 6,2E-03 |
| A_23_P392470 | NM_000901 | 4306 | NR3C2 | nuclear receptor subfamily 3, group C, member 2 | 4 | -0,1 | 4,9E-06 | 6,6E-03 |
| A_23_P128147 | NM_006082 | 10376 | TUBA1B | tubulin, alpha 1b | 12 | 1,7 | 4,9E-06 | 6,6E-03 |
| A_23_P425104 | NM_058243 | 23476 | BRD4 | bromodomain containing 4 | 19 | -0,2 | 5,0E-06 | 6,6E-03 |
| A_23_P45424 | NM_012278 | 26548 | ITGB1BP2 | integrin beta 1 binding protein (melusin) 2 | X | 0,2 | 5,7E-06 | 6,6E-03 |
| A_23_P86012 | NM_001017402 | 3914 | LAMB3 | laminin, beta 3 | 1 | -2,5 | 5,8E-06 | 6,6E-03 |
| A_23_P21072 | AF258583 | 53917 | RAB24 | RAB24, member RAS oncogene family | 5 | -0,2 | 5,9E-06 | 6,6E-03 |
| A_23_P81392 | NM_015238 | 23286 | WWC1 | WW and C2 domain containing 1 | 5 | -1,5 | 6,0E-06 | 6,6E-03 |
| A_23_P147166 | AF516696 | 55799 | CACNA2D3 | calcium channel, voltage-dependent, alpha 2/delta subunit 3 | 3 | -0,1 | 6,1E-06 | 6,6E-03 |
| A_23_P206661 | NM_000903 | 1728 | NQO1 | NAD(P)H dehydrogenase, quinone 1 | 16 | 0,2 | 6,4E-06 | 6,7E-03 |
| A_23_P204427 | NM_002710 | 5501 | PPP1CC | protein phosphatase 1, catalytic subunit, gamma isozyme | 12 | 1,1 | 6,6E-06 | 6,7E-03 |
| A_24_P132518 | NM_001556 | 3551 | IKBKB | inhibitor of kappa light polypeptide gene enhancer in B-cells, kinase beta | 8 | -0,4 | 6,7E-06 | 6,7E-03 |
| A_24_P334130 | NM_054034 | 2335 | FN1 | fibronectin 1 | 2 | -1,2 | 7,0E-06 | 6,7E-03 |
| A_23_P110571 | ENST00000261569 | 375449 | MAST4 | microtubule associated serine/threonine kinase family member 4 | 5 | -0,4 | 7,0E-06 | 6,7E-03 |
| A_23_P2661 | NM_015646 | 5908 | RAP1B | RAP1B, member of RAS oncogene family | 12 | 1,0 | 7,5E-06 | 7,0E-03 |
| A_24_P665504 | BC092421 | 729737 | LOC729737 | hypothetical LOC729737 | 1 | 0,0 | 9,7E-06 | 8,4E-03 |
| A_24_P392333 | NM_001040453 | 54629 | FAM63B | family with sequence similarity 63, member B | 15 | -0,6 | 1,0E-05 | 8,6E-03 |
| A_23_P149529 | NM_002353 | 4070 | TACSTD2 | tumor-associated calcium signal transducer 2 | 1 | -1,6 | 1,0E-05 | 8,6E-03 |
| A_23_P208070 | NM_018439 | 55364 | IMPACT | Impact homolog (mouse) | 18 | 0,5 | 1,0E-05 | 8,6E-03 |
| A_23_P217546 | NM_018196 | 55217 | TMLHE | trimethyllysine hydroxylase, epsilon | X | 0,3 | 1,1E-05 | 8,6E-03 |
| A_23_P123578 | ENST00000320547 | 84726 | PRRC2B | proline-rich coiled-coil 2B | 9 | -0,1 | 1,1E-05 | 8,6E-03 |
| A_32_P222383 | NM_005517 | 3151 | HMGN2 | high mobility group nucleosomal binding domain 2 | 1 | 1,5 | 1,2E-05 | 8,9E-03 |
| A_23_P123276 | NM_003506 | 8323 | FZD6 | frizzled family receptor 6 | 8 | -0,2 | 1,3E-05 | 8,9E-03 |
| A_23_P56505 | NM_000885 | 3676 | ITGA4 | integrin, alpha 4 (antigen CD49D, alpha 4 subunit of VLA-4 receptor) | 2 | 0,4 | 1,4E-05 | 8,9E-03 |
| A_32_P185149 | NM_006913 | 6048 | RNF5 | ring finger protein 5 | 6 | -0,3 | 1,4E-05 | 8,9E-03 |
| A_23_P336554 | NM_134470 | 3556 | IL1RAP | interleukin 1 receptor accessory protein | 3 | 0,1 | 1,4E-05 | 8,9E-03 |
| A_23_P17593 | NM_001794 | 1002 | CDH4 | cadherin 4, type 1, R-cadherin (retinal) | 20 | -0,8 | 1,4E-05 | 8,9E-03 |
| A_23_P63101 | NM_006589 | 10712 | FAM189B | family with sequence similarity 189, member B | 1 | 0,6 | 1,4E-05 | 8,9E-03 |
| A_23_P370434 | NM_001212 | 708 | C1QBP | complement component 1, q subcomponent binding protein | 17 | 1,1 | 1,4E-05 | 8,9E-03 |
| A_23_P107684 | NM_014347 | 25799 | ZNF324 | zinc finger protein 324 | 19 | -0,1 | 1,5E-05 | 8,9E-03 |
| A_23_P16915 | NM_012413 | 25797 | QPCT | glutaminyl-peptide cyclotransferase | 2 | -0,5 | 1,5E-05 | 8,9E-03 |
| A_23_P204751 | NM_020039 | 41 | ACCN2 | amiloride-sensitive cation channel 2, neuronal | 12 | 0,4 | 1,5E-05 | 8,9E-03 |
| A_23_P253375 | NM_001913 | 1523 | CUX1 | cut-like homeobox 1 | 7 | -0,3 | 1,5E-05 | 8,9E-03 |
| A_23_P148410 | NM_031894 | 53940 | FTHL17 | ferritin, heavy polypeptide-like 17 | X | -0,5 | 1,5E-05 | 8,9E-03 |
| A_23_P126970 | NM_006335 | 10440 | TIMM17A | translocase of inner mitochondrial membrane 17 homolog A (yeast) | 1 | 0,7 | 1,5E-05 | 8,9E-03 |
| A_23_P22398 | NM_080676 | 140733 | MACROD2 | MACRO domain containing 2 | 20 | -0,1 | 1,5E-05 | 8,9E-03 |
| A_32_P137939 | NM_001101 | 60 | ACTB | actin, beta | 7 | 0,9 | 1,6E-05 | 8,9E-03 |
| A_32_P21993 | NM_003290 | 7171 | TPM4 | tropomyosin 4 | 19 | 0,7 | 1,6E-05 | 8,9E-03 |
| A_23_P62659 | NM_000310 | 5538 | PPT1 | palmitoyl-protein thioesterase 1 | 1 | 1,0 | 1,6E-05 | 8,9E-03 |
| A_23_P15564 | NM_016627 | 51321 | AMZ2 | archaelysin family metallopeptidase 2 | 17 | 0,5 | 1,8E-05 | 9,7E-03 |
| A_24_P148094 | NM_017526 | 54741 | LEPROT | leptin receptor overlapping transcript | 1 | -0,6 | 1,9E-05 | 9,7E-03 |
| A_23_P37484 | NM_014918 | 22856 | CHSY1 | chondroitin sulfate synthase 1 | 15 | 1,0 | 1,9E-05 | 9,7E-03 |
| A_23_P19938 | NM_006854 | 11014 | KDELR2 | KDEL (Lys-Asp-Glu-Leu) endoplasmic reticulum protein retention receptor 2 | 7 | 1,1 | 1,9E-05 | 9,7E-03 |
| A_23_P57534 | NM_006386 | 10521 | DDX17 | DEAD (Asp-Glu-Ala-Asp) box polypeptide 17 | 22 | -0,1 | 2,0E-05 | 9,7E-03 |
| A_23_P61886 | AK055659 | 10098 | TSPAN5 | tetraspanin 5 | 4 | 1,0 | 2,0E-05 | 9,7E-03 |
| A_23_P391689 | CR602285 | 100131801 | C19orf79 | chromosome 19 open reading frame 79 | 19 | -0,8 | 2,0E-05 | 9,7E-03 |
| A_23_P258621 | NM_003729 | 8634 | RTCD1 | RNA terminal phosphate cyclase domain 1 | 1 | 0,7 | 2,0E-05 | 9,7E-03 |
| A_23_P93722 | NM_152744 | 221935 | SDK1 | sidekick homolog 1, cell adhesion molecule (chicken) | 7 | -0,1 | 2,1E-05 | 9,7E-03 |
| A_23_P52266 | NM_001548 | 3434 | IFIT1 | interferon-induced protein with tetratricopeptide repeats 1 | 10 | 0,5 | 2,1E-05 | 9,7E-03 |
| A_23_P311912 | BC090889 | 113146 | AHNAK2 | AHNAK nucleoprotein 2 | 14 | -0,9 | 2,1E-05 | 9,7E-03 |
| A_32_P25273 | NM_002156 | 3329 | HSPD1 | heat shock 60kDa protein 1 (chaperonin) | 2 | 1,1 | 2,2E-05 | 9,7E-03 |
| A_24_P188325 | NM_015984 | 51377 | UCHL5 | ubiquitin carboxyl-terminal hydrolase L5 | 1 | 0,1 | 2,2E-05 | 9,7E-03 |
| A_23_P398172 | NM_020819 | 57579 | FAM135A | family with sequence similarity 135, member A | 6 | -0,1 | 2,2E-05 | 9,7E-03 |
| A_23_P156117 | NM_001037332 | 26999 | CYFIP2 | cytoplasmic FMR1 interacting protein 2 | 5 | -0,6 | 2,2E-05 | 9,7E-03 |
| A_23_P411953 | NM_138300 | 90780 | PYGO2 | pygopus homolog 2 (Drosophila) | 1 | -0,2 | 2,2E-05 | 9,7E-03 |
| A_23_P250930 | NM_016302 | 51185 | CRBN | cereblon | 3 | -0,2 | 2,3E-05 | 9,8E-03 |
| A_24_P313397 | ENST00000219169 | 10204 | NUTF2 | nuclear transport factor 2 | 16 | -0,3 | 2,4E-05 | 1,0E-02 |
| A_24_P226008 | NM_007283 | 11343 | MGLL | monoglyceride lipase | 3 | -1,2 | 2,4E-05 | 1,0E-02 |
| A_23_P41280 | NM_001079525 | 10606 | PAICS | phosphoribosylaminoimidazole carboxylase, phosphoribosylaminoimidazole succinocarboxamide synthetase | 4 | 1,0 | 2,4E-05 | 1,0E-02 |
| A_32_P101031 | NM_144586 | 116372 | LYPD1 | LY6/PLAUR domain containing 1 | 2 | 2,8 | 2,5E-05 | 1,0E-02 |
| A_23_P141520 | NM_174893 | 124944 | C17orf49 | chromosome 17 open reading frame 49 | 17 | -0,7 | 2,5E-05 | 1,0E-02 |
| A_24_P196038 | NM_018463 | 55846 | ITFG2 | integrin alpha FG-GAP repeat containing 2 | 12 | 0,0 | 2,6E-05 | 1,0E-02 |
| A_23_P431388 | NM_144569 | 90853 | SPOCD1 | SPOC domain containing 1 | 1 | 0,8 | 2,6E-05 | 1,0E-02 |
| A_23_P61447 | NM_004453 | 2110 | ETFDH | electron-transferring-flavoprotein dehydrogenase | 4 | 0,6 | 2,7E-05 | 1,0E-02 |
| A_23_P15714 | NM_006178 | 4905 | NSF | N-ethylmaleimide-sensitive factor | 17 | 0,6 | 2,7E-05 | 1,1E-02 |
| A_23_P88589 | NM_021005 | 7026 | NR2F2 | nuclear receptor subfamily 2, group F, member 2 | 15 | 1,6 | 2,7E-05 | 1,1E-02 |
| A_23_P90296 | NM_004714 | 9149 | DYRK1B | dual-specificity tyrosine-(Y)-phosphorylation regulated kinase 1B | 19 | -0,1 | 2,8E-05 | 1,1E-02 |
| A_23_P165494 | NM_015530 | 26003 | GORASP2 | golgi reassembly stacking protein 2, 55kDa | 2 | 0,2 | 2,8E-05 | 1,1E-02 |
| A_23_P104624 | ENST00000278505 | 23052 | ENDOD1 | endonuclease domain containing 1 | 11 | 0,6 | 2,9E-05 | 1,1E-02 |
| A_23_P252808 | NM_012477 | 23559 | WBP1 | WW domain binding protein 1 | 2 | -0,4 | 2,9E-05 | 1,1E-02 |
| A_23_P394064 | NM_012232 | 284119 | PTRF | polymerase I and transcript release factor | 17 | 1,0 | 2,9E-05 | 1,1E-02 |
| A_32_P164522 | NM_182620 | 348235 | SKA2 | spindle and kinetochore associated complex subunit 2 | 17 | 0,7 | 3,0E-05 | 1,1E-02 |
| A_23_P150446 | NM_006360 | 10480 | EIF3M | eukaryotic translation initiation factor 3, subunit M | 11 | 0,7 | 3,0E-05 | 1,1E-02 |
| A_23_P411379 | NM_178508 | 221491 | C6orf1 | chromosome 6 open reading frame 1 | 6 | -0,2 | 3,0E-05 | 1,1E-02 |
| A_24_P561341 | NM_001002919 | 285016 | FAM150B | family with sequence similarity 150, member B | 2 | -0,2 | 3,0E-05 | 1,1E-02 |
| A_32_P60459 | AB188491 | 220213 | OTUD1 | OTU domain containing 1 | 10 | -0,6 | 3,1E-05 | 1,1E-02 |
| A_23_P43580 | NM_007018 | 11064 | CNTRL | centriolin | 9 | 0,0 | 3,1E-05 | 1,1E-02 |
| A_23_P90099 | NM_198536 | 374882 | TMEM205 | transmembrane protein 205 | 19 | -0,8 | 3,3E-05 | 1,1E-02 |
| A_23_P64617 | NM_012193 | 8322 | FZD4 | frizzled family receptor 4 | 11 | 0,8 | 3,4E-05 | 1,1E-02 |
| A_24_P12573 | NM_006072 | 10344 | CCL26 | chemokine (C-C motif) ligand 26 | 7 | 0,1 | 3,5E-05 | 1,1E-02 |
| A_23_P102113 | NM_025216 | 80326 | WNT10A | wingless-type MMTV integration site family, member 10A | 2 | -1,6 | 3,5E-05 | 1,1E-02 |
| A_23_P71741 | NM_017576 | 55582 | KIF27 | kinesin family member 27 | 9 | -0,1 | 3,6E-05 | 1,2E-02 |
| A_23_P390528 | NM_004420 | 1850 | DUSP8 | dual specificity phosphatase 8 | 11 | -0,6 | 3,7E-05 | 1,2E-02 |
| A_23_P129157 | NM_024608 | 79661 | NEIL1 | nei endonuclease VIII-like 1 (E. coli) | 15 | -0,4 | 3,8E-05 | 1,2E-02 |
| A_23_P75268 | NM_003375 | 7417 | VDAC2 | voltage-dependent anion channel 2 | 10 | 0,6 | 4,1E-05 | 1,3E-02 |
| A_23_P406350 | NM_138431 | 113655 | MFSD3 | major facilitator superfamily domain containing 3 | 8 | -0,4 | 4,1E-05 | 1,3E-02 |
| A_24_P71244 | NM_005026 | 5293 | PIK3CD | phosphoinositide-3-kinase, catalytic, delta polypeptide | 1 | -0,5 | 4,2E-05 | 1,3E-02 |
| A_23_P215897 | NM_183419 | 25897 | RNF19A | ring finger protein 19A | 8 | -0,2 | 4,2E-05 | 1,3E-02 |
| A_23_P156807 | NR_003263 | 8837 | CFLAR | CASP8 and FADD-like apoptosis regulator | 2 | 0,0 | 4,2E-05 | 1,3E-02 |
| A_24_P795371 | AK000872 | 644192 | LOC644192 | hypothetical LOC644192 | 15 | 0,1 | 4,2E-05 | 1,3E-02 |
| A_23_P21644 | NM_016245 | 51170 | HSD17B11 | hydroxysteroid (17-beta) dehydrogenase 11 | 4 | 1,1 | 4,3E-05 | 1,3E-02 |
| A_24_P64182 | NM_014580 | 29988 | SLC2A8 | solute carrier family 2 (facilitated glucose transporter), member 8 | 9 | -0,3 | 4,4E-05 | 1,3E-02 |
| A_23_P423695 | NM_006454 | 10608 | MXD4 | MAX dimerization protein 4 | 4 | -0,2 | 4,5E-05 | 1,3E-02 |
| A_24_P126305 | NM_021646 | 26048 | ZNF500 | zinc finger protein 500 | 16 | -0,2 | 4,5E-05 | 1,3E-02 |
| A_23_P96556 | NM_203391 | 2710 | GK | glycerol kinase | X | 0,1 | 4,5E-05 | 1,3E-02 |
| A_23_P91140 | NM_018441 | 55825 | PECR | peroxisomal trans-2-enoyl-CoA reductase | 2 | 0,1 | 4,7E-05 | 1,3E-02 |
| A_24_P306136 | AL136875 | 57589 | KIAA1432 | KIAA1432 | 9 | 0,1 | 4,7E-05 | 1,3E-02 |
| A_23_P134176 | NM_001024465 | 6648 | SOD2 | superoxide dismutase 2, mitochondrial | 6 | -1,4 | 4,7E-05 | 1,3E-02 |
| A_23_P259272 | NM_018639 | 55884 | WSB2 | WD repeat and SOCS box containing 2 | 12 | 1,3 | 4,8E-05 | 1,3E-02 |
| A_23_P163087 | NM_007361 | 22795 | NID2 | nidogen 2 (osteonidogen) | 14 | 1,1 | 4,8E-05 | 1,3E-02 |
| A_23_P425880 | NM_007118 | 7204 | TRIO | triple functional domain (PTPRF interacting) | 5 | -0,7 | 4,8E-05 | 1,3E-02 |
| A_23_P171296 | NM_002436 | 4354 | MPP1 | membrane protein, palmitoylated 1, 55kDa | X | 0,7 | 4,8E-05 | 1,3E-02 |
| A_24_P312119 | AI084055 | 55033 | FKBP14 | FK506 binding protein 14, 22 kDa | 7 | 0,3 | 5,0E-05 | 1,3E-02 |
| A_23_P57547 | NM_006358 | 10478 | SLC25A17 | solute carrier family 25 (mitochondrial carrier; peroxisomal membrane protein, 34kDa), member 17 | 22 | 0,4 | 5,1E-05 | 1,4E-02 |
| A_23_P111141 | NM_004639 | 7917 | BAG6 | BCL2-associated athanogene 6 | 6 | -0,8 | 5,2E-05 | 1,4E-02 |
| A_23_P129031 | NM_004993 | 4287 | ATXN3 | ataxin 3 | 14 | 0,3 | 5,2E-05 | 1,4E-02 |
| A_23_P141992 | NM_198706 | 374875 | HSD11B1L | hydroxysteroid (11-beta) dehydrogenase 1-like | 19 | -0,2 | 5,5E-05 | 1,4E-02 |
| A_23_P142389 | NM_205834 | 51599 | LSR | lipolysis stimulated lipoprotein receptor | 19 | -0,6 | 5,5E-05 | 1,4E-02 |
| A_32_P231391 | NM_005566 | 3939 | LDHA | lactate dehydrogenase A | 11 | 1,5 | 5,6E-05 | 1,4E-02 |
| A_32_P141374 | NM_178831 | 352954 | GATS | GATS, stromal antigen 3 opposite strand | 7 | -0,1 | 5,7E-05 | 1,5E-02 |
| A_23_P23584 | NM_020248 | 56998 | CTNNBIP1 | catenin, beta interacting protein 1 | 1 | -0,6 | 5,8E-05 | 1,5E-02 |
| A_23_P120056 | NM_033046 | 6242 | RTKN | rhotekin | 2 | -0,3 | 6,0E-05 | 1,5E-02 |
| A_23_P10701 | NM_173075 | 323 | APBB2 | amyloid beta (A4) precursor protein-binding, family B, member 2 | 4 | 0,6 | 6,1E-05 | 1,5E-02 |
| A_23_P163161 | NM_020195 | 56948 | SDR39U1 | short chain dehydrogenase/reductase family 39U, member 1 | 14 | -0,4 | 6,1E-05 | 1,5E-02 |
| A_23_P88209 | NM_016106 | 23256 | SCFD1 | sec1 family domain containing 1 | 14 | 0,6 | 6,1E-05 | 1,5E-02 |
| A_32_P9753 | BC024020 | 81671 | VMP1 | vacuole membrane protein 1 | 17 | -0,4 | 6,1E-05 | 1,5E-02 |
| A_24_P410587 | BC073870 | 201283 | AMZ2P1 | archaelysin family metallopeptidase 2 pseudogene 1 | 17 | 0,4 | 6,3E-05 | 1,5E-02 |
| A_23_P77049 | NM_001039355 | 123096 | SLC25A29 | solute carrier family 25, member 29 | 14 | -0,5 | 6,3E-05 | 1,5E-02 |
| A_23_P359111 | NM_001018067 | 26135 | SERBP1 | SERPINE1 mRNA binding protein 1 | 1 | 0,9 | 6,3E-05 | 1,5E-02 |
| A_23_P304450 | NM_005257 | 2627 | GATA6 | GATA binding protein 6 | 18 | 1,2 | 6,4E-05 | 1,5E-02 |
| A_23_P11744 | NM_199163 | 100287171 | WASH1 | WAS protein family homolog 1 | 9 | -0,8 | 6,6E-05 | 1,5E-02 |
| A_23_P391607 | NM_152285 | 92714 | ARRDC1 | arrestin domain containing 1 | 9 | -0,4 | 6,6E-05 | 1,5E-02 |
| A_24_P136470 | NM_024018 | 54718 | BTN2A3 | butyrophilin, subfamily 2, member A3 | 6 | -0,1 | 6,7E-05 | 1,5E-02 |
| A_23_P123336 | NM_018444 | 54704 | PDP1 | pyruvate dehyrogenase phosphatase catalytic subunit 1 | 8 | -0,5 | 6,8E-05 | 1,5E-02 |
| A_23_P40975 | NM_014674 | 9695 | EDEM1 | ER degradation enhancer, mannosidase alpha-like 1 | 3 | 0,3 | 7,0E-05 | 1,6E-02 |
| A_23_P119362 | NM_001425 | 2014 | EMP3 | epithelial membrane protein 3 | 19 | -0,8 | 7,0E-05 | 1,6E-02 |
| A_23_P134637 | NM_014855 | 9907 | KIAA0415 | KIAA0415 | 7 | -0,4 | 7,3E-05 | 1,6E-02 |
| A_23_P312415 | NM_052866 | 92949 | ADAMTSL1 | ADAMTS-like 1 | 9 | 0,3 | 7,6E-05 | 1,7E-02 |
| A_23_P212511 | NM_001042601 | 151613 | TTC14 | tetratricopeptide repeat domain 14 | 3 | -0,1 | 7,6E-05 | 1,7E-02 |
| A_23_P117928 | NM_014952 | 22893 | BAHD1 | bromo adjacent homology domain containing 1 | 15 | -0,3 | 7,7E-05 | 1,7E-02 |
| A_23_P72627 | NM_004712 | 9146 | HGS | hepatocyte growth factor-regulated tyrosine kinase substrate | 17 | -0,5 | 7,7E-05 | 1,7E-02 |
| A_23_P200252 | NM_032324 | 84284 | NTPCR | nucleoside-triphosphatase, cancer-related | 1 | -0,6 | 7,8E-05 | 1,7E-02 |
| A_24_P51588 | NM_001039577 | 81669 | CCNL2 | cyclin L2 | 1 | -0,6 | 7,9E-05 | 1,7E-02 |
| A_24_P316270 | AK094969 | 55236 | UBA6 | ubiquitin-like modifier activating enzyme 6 | 4 | -0,1 | 7,9E-05 | 1,7E-02 |
| A_24_P237624 | NM_182563 | 283870 | C16orf79 | chromosome 16 open reading frame 79 | 16 | -0,1 | 8,0E-05 | 1,7E-02 |
| A_23_P153070 | NM_017679 | 54828 | BCAS3 | breast carcinoma amplified sequence 3 | 17 | -0,1 | 8,1E-05 | 1,7E-02 |
| A_24_P125690 | NM_023937 | 64981 | MRPL34 | mitochondrial ribosomal protein L34 | 19 | -0,7 | 8,1E-05 | 1,7E-02 |
| A_23_P33196 | NM_000393 | 1290 | COL5A2 | collagen, type V, alpha 2 | 2 | 1,6 | 8,3E-05 | 1,7E-02 |
| A_24_P686247 | DQ786238 | 100506658 | OCLN | occludin | 5 | -0,3 | 8,3E-05 | 1,7E-02 |
| A_24_P335092 | NM_000331 | 6288 | SAA1 | serum amyloid A1 | 11 | -2,0 | 8,4E-05 | 1,7E-02 |
| A_23_P24796 | NM_032127 | 84067 | FAM160A2 | family with sequence similarity 160, member A2 | 11 | -0,4 | 8,4E-05 | 1,7E-02 |
| A_32_P12639 | NM_002816 | 5718 | PSMD12 | proteasome (prosome, macropain) 26S subunit, non-ATPase, 12 | 17 | 0,7 | 8,5E-05 | 1,7E-02 |
| A_23_P26783 | NM_002809 | 5709 | PSMD3 | proteasome (prosome, macropain) 26S subunit, non-ATPase, 3 | 17 | 0,6 | 8,6E-05 | 1,7E-02 |
| A_24_P99152 | NM_004926 | 677 | ZFP36L1 | zinc finger protein 36, C3H type-like 1 | 14 | -0,1 | 8,6E-05 | 1,7E-02 |
| A_23_P130455 | NM_198055 | 7593 | MZF1 | myeloid zinc finger 1 | 19 | -0,3 | 8,6E-05 | 1,7E-02 |
| A_23_P58328 | NM_007193 | 11199 | ANXA10 | annexin A10 | 4 | 0,8 | 8,6E-05 | 1,7E-02 |
| A_24_P388940 | NM_001042493 | 57150 | C6orf162 | chromosome 6 open reading frame 162 | 6 | 0,1 | 8,7E-05 | 1,7E-02 |
| A_23_P101461 | NM_030818 | 81576 | CCDC130 | coiled-coil domain containing 130 | 19 | -0,4 | 8,9E-05 | 1,7E-02 |
| A_24_P271542 | NM_033318 | 91689 | C22orf32 | chromosome 22 open reading frame 32 | 22 | -0,6 | 8,9E-05 | 1,7E-02 |
| A_23_P209636 | NM_006449 | 10602 | CDC42EP3 | CDC42 effector protein (Rho GTPase binding) 3 | 2 | 1,1 | 8,9E-05 | 1,7E-02 |
| A_23_P127805 | BE968596 | 11145 | PLA2G16 | phospholipase A2, group XVI | 11 | -0,3 | 9,1E-05 | 1,8E-02 |
| A_23_P105028 | NM_015459 | 25923 | ATL3 | atlastin GTPase 3 | 11 | 0,4 | 9,1E-05 | 1,8E-02 |
| A_23_P99632 | NM_017999 | 55072 | RNF31 | ring finger protein 31 | 14 | -0,4 | 9,2E-05 | 1,8E-02 |
| A_23_P130316 | NM_004671 | 9063 | PIAS2 | protein inhibitor of activated STAT, 2 | 18 | 0,3 | 9,3E-05 | 1,8E-02 |
| A_23_P423419 | NM_001005266 | 283651 | HMGN2P46 | high mobility group nucleosomal binding domain 2 pseudogene 46 | 15 | 1,8 | 9,3E-05 | 1,8E-02 |
| A_23_P49708 | NM_002087 | 2896 | GRN | granulin | 17 | -1,2 | 9,3E-05 | 1,8E-02 |
| A_23_P432610 | NM_153029 | 9683 | N4BP1 | NEDD4 binding protein 1 | 16 | -0,2 | 9,4E-05 | 1,8E-02 |
| A_24_P88763 | NM_032603 | 84695 | LOXL3 | lysyl oxidase-like 3 | 2 | 1,3 | 9,5E-05 | 1,8E-02 |
| A_23_P126593 | NM_005620 | 6282 | S100A11 | S100 calcium binding protein A11 | 1 | -0,5 | 9,5E-05 | 1,8E-02 |
| A_32_P117145 | NM_018494 | 55367 | PIDD | p53-induced death domain protein | 11 | -0,5 | 9,6E-05 | 1,8E-02 |
| A_24_P300777 | NM_001109 | 101 | ADAM8 | ADAM metallopeptidase domain 8 | 10 | -1,3 | 9,8E-05 | 1,8E-02 |
| A_23_P335661 | AB028976 | 23034 | SAMD4A | sterile alpha motif domain containing 4A | 14 | -0,4 | 1,0E-04 | 1,8E-02 |
| A_23_P400465 | NM_138408 | 112495 | GTF3C6 | general transcription factor IIIC, polypeptide 6, alpha 35kDa | 6 | 1,0 | 1,0E-04 | 1,8E-02 |
| A_24_P161018 | NM_017554 | 54625 | PARP14 | poly (ADP-ribose) polymerase family, member 14 | 3 | -0,3 | 1,0E-04 | 1,9E-02 |
| A_23_P26697 | NM_033452 | 91107 | TRIM47 | tripartite motif containing 47 | 17 | -0,6 | 1,0E-04 | 1,9E-02 |
| A_23_P88602 | NM_170677 | 4212 | MEIS2 | Meis homeobox 2 | 15 | 0,5 | 1,0E-04 | 1,9E-02 |
| A_23_P259580 | NM_172209 | 6892 | TAPBP | TAP binding protein (tapasin) | 6 | -0,1 | 1,0E-04 | 1,9E-02 |
| A_23_P19036 | NM_006058 | 10318 | TNIP1 | TNFAIP3 interacting protein 1 | 5 | -0,8 | 1,1E-04 | 1,9E-02 |
| A_23_P77201 | NM_032499 | 84529 | C15orf41 | chromosome 15 open reading frame 41 | 15 | 0,3 | 1,1E-04 | 1,9E-02 |
| A_23_P325119 | NR_003239 | 128439 | SNHG11 | small nucleolar RNA host gene 11 (non-protein coding) | 20 | -0,2 | 1,1E-04 | 1,9E-02 |
| A_24_P58337 | NM_002032 | 2495 | FTH1 | ferritin, heavy polypeptide 1 | 11 | -1,1 | 1,1E-04 | 1,9E-02 |
| A_23_P15045 | NM_004424 | 1877 | E4F1 | E4F transcription factor 1 | 16 | -0,4 | 1,1E-04 | 1,9E-02 |
| A_23_P355075 | AK023669 | 55839 | CENPN | centromere protein N | 16 | 0,1 | 1,1E-04 | 1,9E-02 |
| A_24_P135276 | NM_032172 | 84132 | USP42 | ubiquitin specific peptidase 42 | 7 | -0,5 | 1,1E-04 | 1,9E-02 |
| A_23_P113005 | NM_004428 | 1942 | EFNA1 | ephrin-A1 | 1 | -0,8 | 1,1E-04 | 1,9E-02 |
| A_23_P8561 | NM_001040457 | 57414 | RHBDD2 | rhomboid domain containing 2 | 7 | -0,4 | 1,1E-04 | 1,9E-02 |
| A_23_P37111 | NM_177438 | 23405 | DICER1 | dicer 1, ribonuclease type III | 14 | -0,4 | 1,2E-04 | 1,9E-02 |
| A_23_P207911 | NM_016113 | 51393 | TRPV2 | transient receptor potential cation channel, subfamily V, member 2 | 17 | 1,2 | 1,2E-04 | 1,9E-02 |
| A_24_P107291 | NM_181699 | 5519 | PPP2R1B | protein phosphatase 2, regulatory subunit A, beta | 11 | 0,2 | 1,2E-04 | 1,9E-02 |
| A_24_P38081 | NM_004117 | 2289 | FKBP5 | FK506 binding protein 5 | 6 | 0,1 | 1,2E-04 | 1,9E-02 |
| A_24_P344087 | NM_001048205 | 9985 | REC8 | REC8 homolog (yeast) | 14 | -0,8 | 1,2E-04 | 2,0E-02 |
| A_23_P41645 | NM_012081 | 22936 | ELL2 | elongation factor, RNA polymerase II, 2 | 5 | 1,3 | 1,2E-04 | 2,0E-02 |
| A_23_P212749 | NM_002111 | 3064 | HTT | huntingtin | 4 | -0,3 | 1,2E-04 | 2,0E-02 |
| A_23_P1782 | NM_002231 | 3732 | CD82 | CD82 molecule | 11 | -1,0 | 1,2E-04 | 2,0E-02 |
| A_24_P44891 | NM_013433 | 30000 | TNPO2 | transportin 2 | 19 | -0,1 | 1,2E-04 | 2,0E-02 |
| A_23_P24594 | NM_006597 | 3312 | HSPA8 | heat shock 70kDa protein 8 | 11 | 0,8 | 1,2E-04 | 2,0E-02 |
| A_23_P63711 | NM_004311 | 403 | ARL3 | ADP-ribosylation factor-like 3 | 10 | 0,7 | 1,2E-04 | 2,0E-02 |
| A_24_P252364 | NM_001037132 | 4897 | NRCAM | neuronal cell adhesion molecule | 7 | -0,1 | 1,3E-04 | 2,0E-02 |
| A_23_P74034 | NM_014412 | 27101 | CACYBP | calcyclin binding protein | 1 | 1,0 | 1,3E-04 | 2,0E-02 |
| A_23_P157215 | NM_014038 | 28969 | BZW2 | basic leucine zipper and W2 domains 2 | 7 | 1,2 | 1,3E-04 | 2,0E-02 |
| A_23_P133408 | NM_000758 | 1437 | CSF2 | colony stimulating factor 2 (granulocyte-macrophage) | 5 | -1,4 | 1,3E-04 | 2,0E-02 |
| A_23_P55076 | NM_130766 | 51763 | INPP5K | inositol polyphosphate-5-phosphatase K | 17 | -0,4 | 1,3E-04 | 2,0E-02 |
| A_24_P346368 | NM_017994 | 55069 | C7orf42 | chromosome 7 open reading frame 42 | 7 | -0,4 | 1,3E-04 | 2,0E-02 |
| A_23_P408473 | AL834145 | 727957 | HEATR7A | HEAT repeat containing 7A | 8 | -0,4 | 1,3E-04 | 2,0E-02 |
| A_23_P372234 | NM_001218 | 771 | CA12 | carbonic anhydrase XII | 15 | 1,0 | 1,3E-04 | 2,0E-02 |
| A_23_P57089 | NM_020182 | 56937 | PMEPA1 | prostate transmembrane protein, androgen induced 1 | 20 | -1,3 | 1,4E-04 | 2,1E-02 |
| A_23_P200186 | NM_032284 | 84950 | PRPF38A | PRP38 pre-mRNA processing factor 38 (yeast) domain containing A | 1 | 0,2 | 1,4E-04 | 2,1E-02 |
| A_23_P118061 | NM_181641 | 51192 | CKLF | chemokine-like factor | 16 | -0,6 | 1,4E-04 | 2,1E-02 |
| A_24_P299794 | NM_005057 | 5929 | RBBP5 | retinoblastoma binding protein 5 | 1 | 0,0 | 1,4E-04 | 2,1E-02 |
| A_32_P179258 | NM_020673 | 57403 | RAB22A | RAB22A, member RAS oncogene family | 20 | -0,1 | 1,4E-04 | 2,1E-02 |
| A_24_P898915 | NM_001004321 | 399844 | FLJ45445 | hypothetical LOC399844 | 19 | -0,4 | 1,4E-04 | 2,1E-02 |
| A_24_P16631 | AK058041 | 100133039 | LOC100133039 | hypothetical LOC100133039 | 3 | -0,1 | 1,4E-04 | 2,1E-02 |
| A_24_P113686 | NM_017953 | 54680 | ZNHIT6 | zinc finger, HIT-type containing 6 | 1 | -0,4 | 1,5E-04 | 2,1E-02 |
| A_23_P250122 | NM_020223 | 56975 | FAM20C | family with sequence similarity 20, member C | 7 | -1,0 | 1,5E-04 | 2,1E-02 |
| A_23_P30024 | NM_003998 | 4790 | NFKB1 | nuclear factor of kappa light polypeptide gene enhancer in B-cells 1 | 4 | -0,5 | 1,5E-04 | 2,1E-02 |
| A_32_P11499 | NM_003348 | 7334 | UBE2N | ubiquitin-conjugating enzyme E2N | 12 | 1,0 | 1,5E-04 | 2,1E-02 |
| A_23_P90634 | NM_152523 | 151195 | CCNYL1 | cyclin Y-like 1 | 2 | 0,4 | 1,5E-04 | 2,1E-02 |
| A_23_P17134 | NM_002371 | 4118 | MAL | mal, T-cell differentiation protein | 2 | -2,6 | 1,5E-04 | 2,1E-02 |
| A_23_P367405 | NM_000281 | 5092 | PCBD1 | pterin-4 alpha-carbinolamine dehydratase/dimerization cofactor of hepatocyte nuclear factor 1 alpha | 10 | -0,7 | 1,5E-04 | 2,1E-02 |
| A_23_P209740 | NM_002807 | 5707 | PSMD1 | proteasome (prosome, macropain) 26S subunit, non-ATPase, 1 | 2 | 0,7 | 1,5E-04 | 2,1E-02 |
| A_23_P422933 | NM_020809 | 57569 | ARHGAP20 | Rho GTPase activating protein 20 | 11 | 0,1 | 1,5E-04 | 2,1E-02 |
| A_23_P37727 | NM_002996 | 6376 | CX3CL1 | chemokine (C-X3-C motif) ligand 1 | 16 | -1,8 | 1,5E-04 | 2,2E-02 |
| A_23_P153586 | NM_006003 | 7386 | UQCRFS1 | ubiquinol-cytochrome c reductase, Rieske iron-sulfur polypeptide 1 | 19 | 0,8 | 1,5E-04 | 2,2E-02 |
| A_23_P5464 | ENST00000273064 | 9125 | RQCD1 | RCD1 required for cell differentiation1 homolog (S. pombe) | 2 | 0,5 | 1,5E-04 | 2,2E-02 |
| A_24_P170826 | NM_006336 | 10444 | ZER1 | zer-1 homolog (C. elegans) | 9 | -0,4 | 1,6E-04 | 2,2E-02 |
| A_23_P399921 | BC026179 | 55176 | SEC61A2 | Sec61 alpha 2 subunit (S. cerevisiae) | 10 | 0,0 | 1,6E-04 | 2,2E-02 |
| A_23_P137196 | NM_001560 | 3597 | IL13RA1 | interleukin 13 receptor, alpha 1 | X | 0,5 | 1,6E-04 | 2,2E-02 |
| A_23_P119627 | NM_015965 | 51079 | NDUFA13 | NADH dehydrogenase (ubiquinone) 1 alpha subcomplex, 13 | 19 | -0,9 | 1,6E-04 | 2,2E-02 |
| A_23_P68007 | NM_001679 | 483 | ATP1B3 | ATPase, Na+/K+ transporting, beta 3 polypeptide | 3 | 0,7 | 1,6E-04 | 2,2E-02 |
| A_23_P12911 | NM_022338 | 53838 | C11orf24 | chromosome 11 open reading frame 24 | 11 | 0,8 | 1,6E-04 | 2,2E-02 |
| A_23_P300600 | NM_021076 | 4744 | NEFH | neurofilament, heavy polypeptide | 22 | 0,8 | 1,6E-04 | 2,2E-02 |
| A_24_P354689 | NM_004598 | 6695 | SPOCK1 | sparc/osteonectin, cwcv and kazal-like domains proteoglycan (testican) 1 | 5 | 0,9 | 1,6E-04 | 2,2E-02 |
| A_23_P217901 | ENST00000368025 | 100131187 | TSTD1 | thiosulfate sulfurtransferase (rhodanese)-like domain containing 1 | 1 | -0,8 | 1,6E-04 | 2,2E-02 |
| A_23_P83939 | NM_032796 | 94056 | SYAP1 | synapse associated protein 1 | X | 0,7 | 1,7E-04 | 2,2E-02 |
| A_24_P89843 | NM_032687 | 50626 | CYHR1 | cysteine/histidine-rich 1 | 8 | -0,3 | 1,7E-04 | 2,2E-02 |
| A_23_P37535 | NM_016530 | 51762 | RAB8B | RAB8B, member RAS oncogene family | 15 | 0,6 | 1,7E-04 | 2,2E-02 |
| A_23_P157970 | NM_014425 | 27130 | INVS | inversin | 9 | 0,1 | 1,7E-04 | 2,2E-02 |
| A_32_P8806 | THC2540396 | 55206 | SBNO1 | strawberry notch homolog 1 (Drosophila) | 12 | -0,2 | 1,7E-04 | 2,2E-02 |
| A_24_P239689 | NM_019072 | 54557 | SGTB | small glutamine-rich tetratricopeptide repeat (TPR)-containing, beta | 5 | 0,1 | 1,7E-04 | 2,2E-02 |
| A_32_P40288 | NM_052913 | 114801 | TMEM200A | transmembrane protein 200A | 6 | 1,1 | 1,7E-04 | 2,2E-02 |
| A_24_P931443 | NM_003485 | 8111 | GPR68 | G protein-coupled receptor 68 | 14 | -0,7 | 1,7E-04 | 2,2E-02 |
| A_23_P356554 | NM_004282 | 9532 | BAG2 | BCL2-associated athanogene 2 | 6 | 0,5 | 1,7E-04 | 2,2E-02 |
| A_23_P201711 | NM_014624 | 6277 | S100A6 | S100 calcium binding protein A6 | 1 | -0,8 | 1,7E-04 | 2,2E-02 |
| A_23_P65000 | NM_013300 | 29902 | C12orf24 | chromosome 12 open reading frame 24 | 12 | 0,3 | 1,7E-04 | 2,2E-02 |
| A_23_P151970 | NM_015322 | 10116 | FEM1B | fem-1 homolog b (C. elegans) | 15 | 0,3 | 1,7E-04 | 2,2E-02 |
| A_24_P100130 | NM_001191 | 598 | BCL2L1 | BCL2-like 1 | 20 | -0,1 | 1,8E-04 | 2,2E-02 |
| A_23_P42695 | NM_024051 | 79017 | GGCT | gamma-glutamylcyclotransferase | 7 | 0,8 | 1,8E-04 | 2,2E-02 |
| A_23_P103720 | NM_024758 | 79814 | AGMAT | agmatine ureohydrolase (agmatinase) | 1 | 0,1 | 1,8E-04 | 2,2E-02 |
| A_24_P207995 | NM_000425 | 3897 | L1CAM | L1 cell adhesion molecule | X | -0,3 | 1,8E-04 | 2,2E-02 |
| A_24_P231132 | NM_001106 | 93 | ACVR2B | activin A receptor, type IIB | 3 | -0,5 | 1,8E-04 | 2,2E-02 |
| A_23_P141479 | NM_003170 | 6830 | SUPT6H | suppressor of Ty 6 homolog (S. cerevisiae) | 17 | -0,3 | 1,8E-04 | 2,2E-02 |
| A_24_P188941 | NM_002520 | 4869 | NPM1 | nucleophosmin (nucleolar phosphoprotein B23, numatrin) | 5 | 1,0 | 1,8E-04 | 2,2E-02 |
| A_24_P215765 | NM_024490 | 57194 | ATP10A | ATPase, class V, type 10A | 15 | 0,8 | 1,8E-04 | 2,2E-02 |
| A_32_P60065 | NM_004101 | 2151 | F2RL2 | coagulation factor II (thrombin) receptor-like 2 | 5 | 1,8 | 1,8E-04 | 2,3E-02 |
| A_23_P61569 | NM_003762 | 8674 | VAMP4 | vesicle-associated membrane protein 4 | 1 | 0,1 | 1,8E-04 | 2,3E-02 |
| A_24_P693946 | THC2585464 | 338657 | CCDC84 | coiled-coil domain containing 84 | 11 | -0,1 | 1,8E-04 | 2,3E-02 |
| A_23_P36345 | NM_006645 | 10809 | STARD10 | StAR-related lipid transfer (START) domain containing 10 | 11 | -0,4 | 1,8E-04 | 2,3E-02 |
| A_23_P52761 | NM_002423 | 4316 | MMP7 | matrix metallopeptidase 7 (matrilysin, uterine) | 11 | -3,3 | 1,9E-04 | 2,3E-02 |
| A_23_P92642 | NM_024668 | 54882 | ANKHD1 | ankyrin repeat and KH domain containing 1 | 5 | 0,2 | 1,9E-04 | 2,3E-02 |
| A_24_P309105 | NM_015450 | 25913 | POT1 | protection of telomeres 1 homolog (S. pombe) | 7 | 0,2 | 1,9E-04 | 2,3E-02 |
| A_23_P168541 | NM_024067 | 79034 | C7orf26 | chromosome 7 open reading frame 26 | 7 | -0,2 | 1,9E-04 | 2,3E-02 |
| A_23_P82412 | NM_001039575 | 55695 | NSUN5 | NOP2/Sun domain family, member 5 | 7 | -0,7 | 1,9E-04 | 2,3E-02 |
| A_24_P206776 | NM_001885 | 1410 | CRYAB | crystallin, alpha B | 11 | -0,6 | 2,0E-04 | 2,3E-02 |
| A_24_P173754 | NM_030806 | 81563 | C1orf21 | chromosome 1 open reading frame 21 | 1 | 0,2 | 2,0E-04 | 2,4E-02 |
| A_23_P428382 | NM_203437 | 54812 | AFTPH | aftiphilin | 2 | -0,2 | 2,0E-04 | 2,4E-02 |
| A_23_P151614 | NM_006263 | 5720 | PSME1 | proteasome (prosome, macropain) activator subunit 1 (PA28 alpha) | 14 | -0,3 | 2,0E-04 | 2,4E-02 |
| A_24_P21447 | NM_006753 | 6838 | SURF6 | surfeit 6 | 9 | 0,1 | 2,0E-04 | 2,4E-02 |
| A_32_P171313 | CR603437 | 59345 | GNB4 | guanine nucleotide binding protein (G protein), beta polypeptide 4 | 3 | 1,0 | 2,0E-04 | 2,4E-02 |
| A_23_P218086 | AB032995 | 53373 | TPCN1 | two pore segment channel 1 | 12 | -1,1 | 2,0E-04 | 2,4E-02 |
| A_24_P410516 | NM_198181 | 440295 | GOLGA6L9 | golgin A6 family-like 9 | 15 | -0,6 | 2,0E-04 | 2,4E-02 |
| A_23_P24345 | NM_152264 | 91252 | SLC39A13 | solute carrier family 39 (zinc transporter), member 13 | 11 | -0,7 | 2,0E-04 | 2,4E-02 |
| A_23_P170581 | AK023048 | 100507962 | LOC100507962 | hypothetical protein LOC100507962 | 16 | 0,0 | 2,1E-04 | 2,4E-02 |
| A_24_P57170 | NM_001039887 | 148137 | C19orf55 | chromosome 19 open reading frame 55 | 19 | 0,0 | 2,1E-04 | 2,4E-02 |
| A_23_P63190 | NM_002524 | 4893 | NRAS | neuroblastoma RAS viral (v-ras) oncogene homolog | 1 | 0,3 | 2,1E-04 | 2,4E-02 |
| A_23_P217028 | NM_001008563 | 10868 | USP20 | ubiquitin specific peptidase 20 | 9 | -0,4 | 2,1E-04 | 2,4E-02 |
| A_23_P48771 | NM_024952 | 80017 | C14orf159 | chromosome 14 open reading frame 159 | 14 | -0,3 | 2,1E-04 | 2,4E-02 |
| A_23_P81973 | NM_014234 | 7923 | HSD17B8 | hydroxysteroid (17-beta) dehydrogenase 8 | 6 | -0,2 | 2,1E-04 | 2,4E-02 |
| A_23_P27613 | U60266 | 4125 | MAN2B1 | mannosidase, alpha, class 2B, member 1 | 19 | -0,7 | 2,1E-04 | 2,4E-02 |
| A_24_P419109 | NM_004381 | 1388 | ATF6B | activating transcription factor 6 beta | 6 | -0,1 | 2,1E-04 | 2,4E-02 |
| A_23_P31315 | NM_016587 | 11335 | CBX3 | chromobox homolog 3 | 7 | 0,8 | 2,1E-04 | 2,4E-02 |
| A_23_P89780 | NM_198129 | 3909 | LAMA3 | laminin, alpha 3 | 18 | -0,6 | 2,1E-04 | 2,4E-02 |
| A_24_P189112 | NM_173830 | 285753 | CEP57L1 | centrosomal protein 57kDa-like 1 | 6 | 0,2 | 2,1E-04 | 2,4E-02 |
| A_23_P105392 | NM_006431 | 10576 | CCT2 | chaperonin containing TCP1, subunit 2 (beta) | 12 | 0,8 | 2,2E-04 | 2,4E-02 |
| A_23_P91081 | NM_002354 | 4072 | EPCAM | epithelial cell adhesion molecule | 2 | -1,4 | 2,2E-04 | 2,4E-02 |
| A_23_P58353 | NM_031370 | 3184 | HNRNPD | heterogeneous nuclear ribonucleoprotein D (AU-rich element RNA binding protein 1, 37kDa) | 4 | 0,9 | 2,2E-04 | 2,4E-02 |
| A_24_P69654 | NM_001300 | 1316 | KLF6 | Kruppel-like factor 6 | 10 | -1,2 | 2,2E-04 | 2,4E-02 |
| A_24_P415208 | BC060806 | 8434 | RECK | reversion-inducing-cysteine-rich protein with kazal motifs | 9 | 0,5 | 2,2E-04 | 2,4E-02 |
| A_23_P90762 | NM_013233 | 27347 | STK39 | serine threonine kinase 39 | 2 | 0,5 | 2,2E-04 | 2,4E-02 |
| A_23_P500844 | NM_001083 | 8654 | PDE5A | phosphodiesterase 5A, cGMP-specific | 4 | 0,0 | 2,2E-04 | 2,4E-02 |
| A_23_P128375 | NM_032829 | 84915 | C12orf34 | chromosome 12 open reading frame 34 | 12 | 0,0 | 2,2E-04 | 2,4E-02 |
| A_23_P337550 | NM_017602 | 55593 | OTUD5 | OTU domain containing 5 | X | -0,5 | 2,2E-04 | 2,4E-02 |
| A_23_P19592 | NM_015599 | 5238 | PGM3 | phosphoglucomutase 3 | 6 | 0,6 | 2,2E-04 | 2,4E-02 |
| A_23_P115313 | NM_022371 | 64222 | TOR3A | torsin family 3, member A | 1 | 0,4 | 2,3E-04 | 2,5E-02 |
| A_23_P122662 | NM_018988 | 54438 | GFOD1 | glucose-fructose oxidoreductase domain containing 1 | 6 | 0,4 | 2,3E-04 | 2,5E-02 |
| A_23_P89327 | NM_020652 | 57335 | ZNF286A | zinc finger protein 286A | 17 | 0,1 | 2,3E-04 | 2,5E-02 |
| A_24_P244162 | NM_018425 | 55361 | PI4K2A | phosphatidylinositol 4-kinase type 2 alpha | 10 | -0,3 | 2,3E-04 | 2,5E-02 |
| A_24_P296568 | NM_006807 | 10951 | CBX1 | chromobox homolog 1 | 17 | 1,0 | 2,3E-04 | 2,5E-02 |
| A_23_P106727 | NM_014700 | 9727 | RAB11FIP3 | RAB11 family interacting protein 3 (class II) | 16 | -0,5 | 2,3E-04 | 2,5E-02 |
| A_24_P45160 | NM_001039842 | 339229 | C17orf90 | chromosome 17 open reading frame 90 | 17 | -0,4 | 2,3E-04 | 2,5E-02 |
| A_24_P247408 | AY129010 | 399851 | LOC399851 | hypothetical LOC399851 | 11 | 0,0 | 2,3E-04 | 2,5E-02 |
| A_23_P321630 | NM_020810 | 57570 | TRMT5 | TRM5 tRNA methyltransferase 5 homolog (S. cerevisiae) | 14 | 1,4 | 2,3E-04 | 2,5E-02 |
| A_23_P114879 | NM_015888 | 51361 | HOOK1 | hook homolog 1 (Drosophila) | 1 | -0,1 | 2,3E-04 | 2,5E-02 |
| A_24_P73370 | NM_003565 | 8408 | ULK1 | unc-51-like kinase 1 (C. elegans) | 12 | -0,4 | 2,4E-04 | 2,5E-02 |
| A_32_P32250 | NM_022063 | 63877 | FAM204A | family with sequence similarity 204, member A | 10 | 0,4 | 2,4E-04 | 2,5E-02 |
| A_23_P103795 | NM_138959 | 81839 | VANGL1 | vang-like 1 (van gogh, Drosophila) | 1 | 0,2 | 2,4E-04 | 2,5E-02 |
| A_23_P255436 | NM_000487 | 410 | ARSA | arylsulfatase A | 22 | -0,3 | 2,4E-04 | 2,5E-02 |
| A_32_P147078 | NM_021097 | 6546 | SLC8A1 | solute carrier family 8 (sodium/calcium exchanger), member 1 | 2 | 1,2 | 2,4E-04 | 2,5E-02 |
| A_23_P117540 | NM_145251 | 6815 | STYX | serine/threonine/tyrosine interacting protein | 14 | 0,2 | 2,4E-04 | 2,5E-02 |
| A_23_P369210 | X91906 | 1184 | CLCN5 | chloride channel 5 | X | 0,0 | 2,5E-04 | 2,5E-02 |
| A_23_P84063 | NM_016522 | 50863 | NTM | neurotrimin | 11 | -0,5 | 2,5E-04 | 2,5E-02 |
| A_23_P64879 | NM_004982 | 3764 | KCNJ8 | potassium inwardly-rectifying channel, subfamily J, member 8 | 12 | -0,4 | 2,5E-04 | 2,5E-02 |
| A_23_P418597 | NM_033396 | 85456 | TNKS1BP1 | tankyrase 1 binding protein 1, 182kDa | 11 | -0,1 | 2,5E-04 | 2,5E-02 |
| A_24_P193011 | NM_053056 | 595 | CCND1 | cyclin D1 | 11 | -0,9 | 2,5E-04 | 2,5E-02 |
| A_32_P161661 | ENST00000377711 | 203062 | TSNARE1 | t-SNARE domain containing 1 | 8 | -0,3 | 2,5E-04 | 2,5E-02 |
| A_23_P56938 | NM_002908 | 5966 | REL | v-rel reticuloendotheliosis viral oncogene homolog (avian) | 2 | -0,1 | 2,5E-04 | 2,5E-02 |
| A_32_P213831 | NM_020704 | 57464 | FAM40B | family with sequence similarity 40, member B | 7 | 0,0 | 2,5E-04 | 2,5E-02 |
| A_24_P163537 | NM_182547 | 222068 | TMED4 | transmembrane emp24 protein transport domain containing 4 | 7 | -0,4 | 2,5E-04 | 2,5E-02 |
| A_23_P156732 | NM_024165 | 5252 | PHF1 | PHD finger protein 1 | 6 | -0,3 | 2,5E-04 | 2,5E-02 |
| A_23_P102420 | NM_006430 | 10575 | CCT4 | chaperonin containing TCP1, subunit 4 (delta) | 2 | 0,8 | 2,5E-04 | 2,5E-02 |
| A_32_P175301 | NM_014957 | 22898 | DENND3 | DENN/MADD domain containing 3 | 8 | -0,7 | 2,5E-04 | 2,5E-02 |
| A_23_P251717 | NM_007075 | 11152 | WDR45 | WD repeat domain 45 | X | -0,6 | 2,6E-04 | 2,5E-02 |
| A_23_P431638 | BC029662 | 128486 | FITM2 | fat storage-inducing transmembrane protein 2 | 20 | 0,3 | 2,6E-04 | 2,5E-02 |
| A_24_P225468 | NM_030920 | 81611 | ANP32E | acidic (leucine-rich) nuclear phosphoprotein 32 family, member E | 1 | 0,4 | 2,6E-04 | 2,5E-02 |
| A_32_P24741 | AK093729 | 402483 | FLJ45340 | hypothetical LOC402483 | 7 | -0,3 | 2,6E-04 | 2,5E-02 |
| A_23_P206400 | AB020679 | 22879 | MON1B | MON1 homolog B (yeast) | 16 | -0,1 | 2,6E-04 | 2,5E-02 |
| A_23_P371410 | NM_207578 | 5567 | PRKACB | protein kinase, cAMP-dependent, catalytic, beta | 1 | 0,1 | 2,6E-04 | 2,5E-02 |
| A_23_P368645 | NM_003659 | 8540 | AGPS | alkylglycerone phosphate synthase | 2 | 0,3 | 2,6E-04 | 2,5E-02 |
| A_23_P137276 | NM_006738 | 11214 | AKAP13 | A kinase (PRKA) anchor protein 13 | 15 | -0,5 | 2,6E-04 | 2,5E-02 |
| A_24_P257416 | NM_002089 | 2920 | CXCL2 | chemokine (C-X-C motif) ligand 2 | 4 | -1,2 | 2,7E-04 | 2,5E-02 |
| A_32_P213091 | AK094972 | 729993 | SHISA9 | shisa homolog 9 (Xenopus laevis) | 16 | -0,4 | 2,7E-04 | 2,5E-02 |
| A_23_P171366 | NM_004651 | 8237 | USP11 | ubiquitin specific peptidase 11 | X | -0,5 | 2,7E-04 | 2,5E-02 |
| A_23_P159956 | NM_012216 | 11043 | MID2 | midline 2 | X | 0,1 | 2,7E-04 | 2,5E-02 |
| A_32_P153195 | ENST00000299694 | 146227 | BEAN1 | brain expressed, associated with NEDD4, 1 | 16 | -1,1 | 2,7E-04 | 2,5E-02 |
| A_24_P185854 | NM_004010 | 1756 | DMD | dystrophin | X | 0,9 | 2,7E-04 | 2,5E-02 |
| A_23_P201035 | NM_001005749 | 2629 | GBA | glucosidase, beta, acid | 1 | -0,5 | 2,7E-04 | 2,5E-02 |
| A_23_P66355 | NM_000213 | 3691 | ITGB4 | integrin, beta 4 | 17 | -0,5 | 2,7E-04 | 2,5E-02 |
| A_24_P281913 | AF272382 | 4297 | MLL | myeloid/lymphoid or mixed-lineage leukemia (trithorax homolog, Drosophila) | 11 | -0,6 | 2,7E-04 | 2,5E-02 |
| A_32_P117354 | NM_014988 | 22998 | LIMCH1 | LIM and calponin homology domains 1 | 4 | -0,9 | 2,7E-04 | 2,5E-02 |
| A_23_P47777 | NM_138396 | 92979 | MARCH9 | membrane-associated ring finger (C3HC4) 9 | 12 | -0,3 | 2,7E-04 | 2,5E-02 |
| A_24_P111106 | NM_000800 | 2246 | FGF1 | fibroblast growth factor 1 (acidic) | 5 | 0,8 | 2,7E-04 | 2,5E-02 |
| A_23_P68486 | NM_080821 | 116151 | C20orf108 | chromosome 20 open reading frame 108 | 20 | -0,6 | 2,7E-04 | 2,5E-02 |
| A_24_P111134 | NM_013382 | 29954 | POMT2 | protein-O-mannosyltransferase 2 | 14 | -0,3 | 2,7E-04 | 2,5E-02 |
| A_23_P208812 | NM_014910 | 22847 | ZNF507 | zinc finger protein 507 | 19 | 0,1 | 2,8E-04 | 2,6E-02 |
| A_23_P154367 | NM_004226 | 9262 | STK17B | serine/threonine kinase 17b | 2 | 0,1 | 2,8E-04 | 2,6E-02 |
| A_23_P114232 | NM_006406 | 10549 | PRDX4 | peroxiredoxin 4 | X | 0,7 | 2,8E-04 | 2,6E-02 |
| A_23_P82169 | NM_003107 | 6659 | SOX4 | SRY (sex determining region Y)-box 4 | 6 | -1,2 | 2,8E-04 | 2,6E-02 |
| A_24_P943040 | NM_198066 | 64841 | GNPNAT1 | glucosamine-phosphate N-acetyltransferase 1 | 14 | 0,4 | 2,8E-04 | 2,6E-02 |
| A_23_P501822 | NM_002230 | 3728 | JUP | junction plakoglobin | 17 | -1,1 | 2,8E-04 | 2,6E-02 |
| A_23_P164284 | NM_001307 | 1366 | CLDN7 | claudin 7 | 17 | -0,7 | 2,8E-04 | 2,6E-02 |
| A_23_P25150 | NM_006897 | 3225 | HOXC9 | homeobox C9 | 12 | 1,0 | 2,8E-04 | 2,6E-02 |
| A_32_P98683 | AL832481 | 4302 | MLLT6 | myeloid/lymphoid or mixed-lineage leukemia (trithorax homolog, Drosophila); translocated to, 6 | 17 | -0,5 | 2,8E-04 | 2,6E-02 |
| A_24_P242021 | NM_001007467 | 9814 | SFI1 | Sfi1 homolog, spindle assembly associated (yeast) | 22 | -0,1 | 2,8E-04 | 2,6E-02 |
| A_23_P151337 | NR_002605 | 10301 | DLEU1 | deleted in lymphocytic leukemia 1 (non-protein coding) | 13 | 0,4 | 2,8E-04 | 2,6E-02 |
| A_23_P55731 | NM_015125 | 23152 | CIC | capicua homolog (Drosophila) | 19 | -0,3 | 2,9E-04 | 2,6E-02 |
| A_24_P237278 | NM_003634 | 8508 | NIPSNAP1 | nipsnap homolog 1 (C. elegans) | 22 | -0,3 | 2,9E-04 | 2,6E-02 |
| A_23_P43566 | NM_014222 | 4702 | NDUFA8 | NADH dehydrogenase (ubiquinone) 1 alpha subcomplex, 8, 19kDa | 9 | 0,7 | 2,9E-04 | 2,6E-02 |
| A_24_P56252 | AF086032 | 4659 | PPP1R12A | protein phosphatase 1, regulatory (inhibitor) subunit 12A | 12 | 0,7 | 2,9E-04 | 2,6E-02 |
| A_23_P164883 | NM_007254 | 11284 | PNKP | polynucleotide kinase 3'-phosphatase | 19 | -0,2 | 2,9E-04 | 2,6E-02 |
| A_23_P76090 | NM_005419 | 6773 | STAT2 | signal transducer and activator of transcription 2, 113kDa | 12 | -0,1 | 2,9E-04 | 2,6E-02 |
| A_32_P47643 | CR601458 | 642273 | FAM110C | family with sequence similarity 110, member C | 2 | -0,8 | 2,9E-04 | 2,6E-02 |
| A_32_P152767 | NM_207371 | 387640 | C10orf140 | chromosome 10 open reading frame 140 | 10 | 0,1 | 2,9E-04 | 2,6E-02 |
| A_24_P919304 | BC012204 | 64778 | FNDC3B | fibronectin type III domain containing 3B | 3 | 0,1 | 2,9E-04 | 2,6E-02 |
| A_23_P200737 | NM_005613 | 5999 | RGS4 | regulator of G-protein signaling 4 | 1 | 1,7 | 2,9E-04 | 2,6E-02 |
| A_32_P66364 | BC017943 | 151242 | PPP1R1C | protein phosphatase 1, regulatory (inhibitor) subunit 1C | 2 | 0,0 | 3,0E-04 | 2,6E-02 |
| A_24_P176044 | NM_012063 | 10059 | DNM1L | dynamin 1-like | 12 | 0,4 | 3,0E-04 | 2,6E-02 |
| A_23_P101303 | ENST00000221462 | 284352 | LRRC68 | leucine rich repeat containing 68 | 19 | -0,3 | 3,0E-04 | 2,6E-02 |
| A_24_P133475 | NM_138452 | 115817 | DHRS1 | dehydrogenase/reductase (SDR family) member 1 | 14 | -0,5 | 3,0E-04 | 2,6E-02 |
| A_23_P169154 | NM_024896 | 79956 | ERMP1 | endoplasmic reticulum metallopeptidase 1 | 9 | -0,1 | 3,0E-04 | 2,6E-02 |
| A_23_P38046 | NM_024535 | 79585 | CORO7 | coronin 7 | 16 | -0,3 | 3,0E-04 | 2,6E-02 |
| A_23_P200260 | NM_014801 | 80003 | PCNXL2 | pecanex-like 2 (Drosophila) | 1 | -0,4 | 3,0E-04 | 2,6E-02 |
| A_24_P350124 | NM_020954 | 57674 | RNF213 | ring finger protein 213 | 17 | -0,7 | 3,0E-04 | 2,6E-02 |
| A_23_P435407 | NM_001448 | 2239 | GPC4 | glypican 4 | X | -0,4 | 3,0E-04 | 2,6E-02 |
| A_23_P170733 | NM_058172 | 118429 | ANTXR2 | anthrax toxin receptor 2 | 4 | 1,3 | 3,0E-04 | 2,6E-02 |
| A_23_P140035 | NM_007187 | 11193 | WBP4 | WW domain binding protein 4 (formin binding protein 21) | 13 | 0,7 | 3,0E-04 | 2,6E-02 |
| A_23_P16562 | NM_001017392 | 10147 | SUGP2 | SURP and G patch domain containing 2 | 19 | -0,1 | 3,0E-04 | 2,6E-02 |
| A_23_P129334 | NM_001287 | 1186 | CLCN7 | chloride channel 7 | 16 | -0,2 | 3,1E-04 | 2,6E-02 |
| A_23_P153320 | NM_000201 | 3383 | ICAM1 | intercellular adhesion molecule 1 | 19 | -0,8 | 3,1E-04 | 2,6E-02 |
| A_23_P91964 | NM_006506 | 5922 | RASA2 | RAS p21 protein activator 2 | 3 | -0,1 | 3,1E-04 | 2,6E-02 |
| A_24_P29401 | NM_181523 | 5295 | PIK3R1 | phosphoinositide-3-kinase, regulatory subunit 1 (alpha) | 5 | -0,2 | 3,1E-04 | 2,6E-02 |
| A_23_P254271 | NM_032525 | 84617 | TUBB6 | tubulin, beta 6 | 18 | 1,1 | 3,1E-04 | 2,6E-02 |
| A_23_P32444 | NM_032348 | 54587 | MXRA8 | matrix-remodelling associated 8 | 1 | 1,2 | 3,1E-04 | 2,6E-02 |
| A_23_P82503 | NM_001040152 | 23089 | PEG10 | paternally expressed 10 | 7 | 0,3 | 3,1E-04 | 2,6E-02 |
| A_23_P376488 | NM_000594 | 7124 | TNF | tumor necrosis factor | 6 | -1,5 | 3,1E-04 | 2,6E-02 |
| A_23_P252201 | NM_018456 | 55840 | EAF2 | ELL associated factor 2 | 3 | 0,1 | 3,1E-04 | 2,6E-02 |
| A_23_P257988 | AF151697 | 59343 | SENP2 | SUMO1/sentrin/SMT3 specific peptidase 2 | 3 | 0,1 | 3,1E-04 | 2,6E-02 |
| A_23_P27827 | NM_015898 | 51341 | ZBTB7A | zinc finger and BTB domain containing 7A | 19 | -0,4 | 3,1E-04 | 2,6E-02 |
| A_23_P104579 | NM_005851 | 10263 | CDK2AP2 | cyclin-dependent kinase 2 associated protein 2 | 11 | -0,5 | 3,2E-04 | 2,6E-02 |
| A_23_P88201 | NM_017917 | 55012 | PPP2R3C | protein phosphatase 2, regulatory subunit B'', gamma | 14 | 0,7 | 3,2E-04 | 2,6E-02 |
| A_23_P103756 | NM_002557 | 5016 | OVGP1 | oviductal glycoprotein 1, 120kDa | 1 | -0,1 | 3,2E-04 | 2,6E-02 |
| A_23_P104201 | NM_139313 | 10730 | YME1L1 | YME1-like 1 (S. cerevisiae) | 10 | 0,6 | 3,2E-04 | 2,6E-02 |
| A_24_P113815 | NM_182838 | 9906 | SLC35E2 | solute carrier family 35, member E2 | 1 | -0,1 | 3,2E-04 | 2,6E-02 |
| A_23_P121795 | NM_021069 | 8470 | SORBS2 | sorbin and SH3 domain containing 2 | 4 | -0,2 | 3,2E-04 | 2,6E-02 |
| A_24_P110967 | NM_052937 | 115294 | PCMTD1 | protein-L-isoaspartate (D-aspartate) O-methyltransferase domain containing 1 | 8 | -0,1 | 3,2E-04 | 2,6E-02 |
| A_23_P35349 | NM_021738 | 6840 | SVIL | supervillin | 10 | -0,8 | 3,3E-04 | 2,6E-02 |
| A_23_P158053 | NM_024112 | 79095 | C9orf16 | chromosome 9 open reading frame 16 | 9 | -0,2 | 3,3E-04 | 2,7E-02 |
| A_23_P157580 | NM_005625 | 6386 | SDCBP | syndecan binding protein (syntenin) | 8 | -0,7 | 3,3E-04 | 2,7E-02 |
| A_23_P91619 | NM_002415 | 4282 | MIF | macrophage migration inhibitory factor (glycosylation-inhibiting factor) | 22 | 0,6 | 3,3E-04 | 2,7E-02 |
| A_24_P22976 | NM_001025604 | 27106 | ARRDC2 | arrestin domain containing 2 | 19 | -0,2 | 3,3E-04 | 2,7E-02 |
| A_32_P402521 | NM_006445 | 10594 | PRPF8 | PRP8 pre-mRNA processing factor 8 homolog (S. cerevisiae) | 17 | -0,6 | 3,3E-04 | 2,7E-02 |
| A_23_P75028 | NM_001001330 | 221035 | REEP3 | receptor accessory protein 3 | 10 | 0,7 | 3,3E-04 | 2,7E-02 |
| A_32_P100109 | ENST00000380064 | 9185 | REPS2 | RALBP1 associated Eps domain containing 2 | X | 0,0 | 3,3E-04 | 2,7E-02 |
| A_23_P417415 | NM_147161 | 26027 | ACOT11 | acyl-CoA thioesterase 11 | 1 | 0,2 | 3,4E-04 | 2,7E-02 |
| A_24_P104115 | NM_019034 | 54509 | RHOF | ras homolog gene family, member F (in filopodia) | 12 | -0,3 | 3,4E-04 | 2,7E-02 |
| A_23_P1722 | NM_080659 | 91894 | C11orf52 | chromosome 11 open reading frame 52 | 11 | -0,2 | 3,4E-04 | 2,7E-02 |
| A_23_P28638 | NM_017958 | 55041 | PLEKHB2 | pleckstrin homology domain containing, family B (evectins) member 2 | 2 | -0,3 | 3,4E-04 | 2,7E-02 |
| A_23_P250283 | NM_004161 | 5861 | RAB1A | RAB1A, member RAS oncogene family | 2 | 0,6 | 3,4E-04 | 2,7E-02 |
| A_23_P301138 | NM_001005910 | 51447 | IP6K2 | inositol hexakisphosphate kinase 2 | 3 | -0,6 | 3,4E-04 | 2,7E-02 |
| A_24_P205181 | NM_020205 | 56957 | OTUD7B | OTU domain containing 7B | 1 | 0,0 | 3,4E-04 | 2,7E-02 |
| A_23_P154605 | NM_018837 | 55959 | SULF2 | sulfatase 2 | 20 | -1,1 | 3,4E-04 | 2,7E-02 |
| A_23_P41777 | NM_024715 | 79770 | TXNDC15 | thioredoxin domain containing 15 | 5 | 0,8 | 3,4E-04 | 2,7E-02 |
| A_23_P144916 | NM_005110 | 9945 | GFPT2 | glutamine-fructose-6-phosphate transaminase 2 | 5 | 0,7 | 3,4E-04 | 2,7E-02 |
| A_23_P152305 | NM_001797 | 1009 | CDH11 | cadherin 11, type 2, OB-cadherin (osteoblast) | 16 | 1,8 | 3,4E-04 | 2,7E-02 |
| A_23_P18422 | NM_007208 | 11222 | MRPL3 | mitochondrial ribosomal protein L3 | 3 | 0,6 | 3,5E-04 | 2,7E-02 |
| A_23_P38427 | NM_032932 | 84440 | RAB11FIP4 | RAB11 family interacting protein 4 (class II) | 17 | -0,1 | 3,5E-04 | 2,7E-02 |
| A_23_P431404 | AJ278120 | 26115 | TANC2 | tetratricopeptide repeat, ankyrin repeat and coiled-coil containing 2 | 17 | 0,0 | 3,5E-04 | 2,7E-02 |
| A_23_P87560 | NM_001731 | 694 | BTG1 | B-cell translocation gene 1, anti-proliferative | 12 | -0,6 | 3,5E-04 | 2,7E-02 |
| A_23_P73747 | NM_014782 | 9823 | ARMCX2 | armadillo repeat containing, X-linked 2 | X | 0,8 | 3,5E-04 | 2,7E-02 |
| A_23_P94365 | NM_174922 | 203054 | ADCK5 | aarF domain containing kinase 5 | 8 | -0,3 | 3,6E-04 | 2,7E-02 |
| A_24_P166661 | NM_032936 | 85025 | TMEM60 | transmembrane protein 60 | 7 | -0,2 | 3,6E-04 | 2,7E-02 |
| A_32_P175198 | NM_001614 | 71 | ACTG1 | actin, gamma 1 | 17 | 0,6 | 3,6E-04 | 2,8E-02 |
| A_24_P260134 | NM_178177 | 349565 | NMNAT3 | nicotinamide nucleotide adenylyltransferase 3 | 3 | -0,2 | 3,7E-04 | 2,8E-02 |
| A_23_P91891 | NM_004766 | 9276 | COPB2 | coatomer protein complex, subunit beta 2 (beta prime) | 3 | 0,6 | 3,7E-04 | 2,8E-02 |
| A_23_P379649 | NM_001003940 | 90427 | BMF | Bcl2 modifying factor | 15 | -0,5 | 3,7E-04 | 2,8E-02 |
| A_23_P253046 | NM_006759 | 7360 | UGP2 | UDP-glucose pyrophosphorylase 2 | 2 | 0,5 | 3,7E-04 | 2,8E-02 |
| A_23_P302634 | NM_173591 | 283310 | OTOGL | otogelin-like | 12 | 0,0 | 3,8E-04 | 2,8E-02 |
| A_23_P28075 | NM_001039848 | 2879 | GPX4 | glutathione peroxidase 4 (phospholipid hydroperoxidase) | 19 | -1,0 | 3,8E-04 | 2,9E-02 |
| A_32_P37089 | AK097700 | 146880 | LOC146880 | hypothetical LOC146880 | 17 | -0,1 | 3,8E-04 | 2,9E-02 |
| A_32_P194004 | AK131472 | 57615 | ZNF492 | zinc finger protein 492 | 19 | -0,2 | 3,8E-04 | 2,9E-02 |
| A_24_P197964 | NM_014788 | 9830 | TRIM14 | tripartite motif containing 14 | 9 | 0,1 | 3,8E-04 | 2,9E-02 |
| A_24_P374759 | NM_015576 | 26059 | ERC2 | ELKS/RAB6-interacting/CAST family member 2 | 3 | -0,1 | 3,9E-04 | 2,9E-02 |
| A_23_P49060 | NM_181642 | 6692 | SPINT1 | serine peptidase inhibitor, Kunitz type 1 | 15 | -0,4 | 3,9E-04 | 2,9E-02 |
| A_23_P135878 | NM_020148 | 56907 | SPIRE1 | spire homolog 1 (Drosophila) | 18 | 0,3 | 3,9E-04 | 2,9E-02 |
| A_23_P22072 | CR625878 | 8847 | DLEU2 | deleted in lymphocytic leukemia 2 (non-protein coding) | 13 | 0,1 | 3,9E-04 | 2,9E-02 |
| A_23_P163258 | NM_020214 | 56965 | PARP6 | poly (ADP-ribose) polymerase family, member 6 | 15 | -0,2 | 3,9E-04 | 2,9E-02 |
| A_24_P124558 | NM_022658 | 3224 | HOXC8 | homeobox C8 | 12 | 0,3 | 3,9E-04 | 2,9E-02 |
| A_23_P394304 | NM_005764 | 10158 | PDZK1IP1 | PDZK1 interacting protein 1 | 1 | -1,1 | 3,9E-04 | 2,9E-02 |
| A_32_P234935 | NM_007375 | 23435 | TARDBP | TAR DNA binding protein | 1 | 0,9 | 4,0E-04 | 2,9E-02 |
| A_23_P57227 | NM_003098 | 6640 | SNTA1 | syntrophin, alpha 1 (dystrophin-associated protein A1, 59kDa, acidic component) | 20 | 0,3 | 4,0E-04 | 2,9E-02 |
| A_24_P398790 | AK024113 | 55051 | C14orf102 | chromosome 14 open reading frame 102 | 14 | 0,0 | 4,0E-04 | 2,9E-02 |
| A_23_P152420 | NM_014615 | 23199 | KIAA0182 | KIAA0182 | 16 | -0,2 | 4,0E-04 | 2,9E-02 |
| A_23_P256432 | NM_006243 | 5525 | PPP2R5A | protein phosphatase 2, regulatory subunit B', alpha | 1 | 0,5 | 4,0E-04 | 2,9E-02 |
| A_23_P9280 | ENST00000376347 | 389765 | LOC389765 | kinesin family member 27 pseudogene | 9 | -0,1 | 4,1E-04 | 3,0E-02 |
| A_23_P112078 | NM_004225 | 9258 | MFHAS1 | malignant fibrous histiocytoma amplified sequence 1 | 8 | -0,2 | 4,1E-04 | 3,0E-02 |
| A_24_P416059 | NM_006715 | 4123 | MAN2C1 | mannosidase, alpha, class 2C, member 1 | 15 | -0,3 | 4,1E-04 | 3,0E-02 |
| A_23_P53126 | NM_005574 | 4005 | LMO2 | LIM domain only 2 (rhombotin-like 1) | 11 | 0,1 | 4,1E-04 | 3,0E-02 |
| A_23_P69617 | NM_003728 | 8633 | UNC5C | unc-5 homolog C (C. elegans) | 4 | 0,1 | 4,1E-04 | 3,0E-02 |
| A_23_P328069 | NM_000195 | 3257 | HPS1 | Hermansky-Pudlak syndrome 1 | 10 | -0,2 | 4,1E-04 | 3,0E-02 |
| A_24_P347488 | NM_002802 | 5700 | PSMC1 | proteasome (prosome, macropain) 26S subunit, ATPase, 1 | 14 | 0,7 | 4,2E-04 | 3,0E-02 |
| A_23_P79818 | NM_016470 | 51526 | C20orf111 | chromosome 20 open reading frame 111 | 20 | 0,1 | 4,2E-04 | 3,0E-02 |
| A_23_P93348 | NM_002341 | 4050 | LTB | lymphotoxin beta (TNF superfamily, member 3) | 6 | -2,1 | 4,2E-04 | 3,0E-02 |
| A_24_P48014 | NM_003745 | 8651 | SOCS1 | suppressor of cytokine signaling 1 | 16 | 0,0 | 4,2E-04 | 3,0E-02 |
| A_23_P170453 | NM_001900 | 1473 | CST5 | cystatin D | 20 | -0,1 | 4,3E-04 | 3,0E-02 |
| A_23_P5778 | NM_022449 | 64284 | RAB17 | RAB17, member RAS oncogene family | 2 | -0,1 | 4,3E-04 | 3,0E-02 |
| A_23_P57784 | NM_021101 | 9076 | CLDN1 | claudin 1 | 3 | -1,4 | 4,3E-04 | 3,0E-02 |
| A_23_P110052 | NM_023067 | 668 | FOXL2 | forkhead box L2 | 3 | -0,4 | 4,3E-04 | 3,0E-02 |
| A_23_P14673 | NM_020962 | 57722 | IGDCC4 | immunoglobulin superfamily, DCC subclass, member 4 | 15 | -0,5 | 4,4E-04 | 3,1E-02 |
| A_23_P126474 | NM_003145 | 6746 | SSR2 | signal sequence receptor, beta (translocon-associated protein beta) | 1 | 0,8 | 4,4E-04 | 3,1E-02 |
| A_24_P324814 | NM_138363 | 90799 | CEP95 | centrosomal protein 95kDa | 17 | -0,1 | 4,4E-04 | 3,1E-02 |
| A_23_P431330 | NM_175918 | 285464 | CRIPAK | cysteine-rich PAK1 inhibitor | 4 | -0,2 | 4,4E-04 | 3,1E-02 |
| A_23_P167093 | NM_000203 | 3425 | IDUA | iduronidase, alpha-L- | 4 | -0,4 | 4,4E-04 | 3,1E-02 |
| A_23_P43779 | NM_016129 | 51138 | COPS4 | COP9 constitutive photomorphogenic homolog subunit 4 (Arabidopsis) | 4 | 0,5 | 4,4E-04 | 3,1E-02 |
| A_23_P41021 | NM_007184 | 11188 | NISCH | nischarin | 3 | -0,8 | 4,4E-04 | 3,1E-02 |
| A_24_P350708 | AK125313 | 100505915 | LOC100505915 | hypothetical LOC100505915 | 16 | -0,1 | 4,4E-04 | 3,1E-02 |
| A_24_P148043 | NM_014864 | 9917 | FAM20B | family with sequence similarity 20, member B | 1 | 0,7 | 4,5E-04 | 3,1E-02 |
| A_23_P36647 | NM_177441 | 79089 | TMUB2 | transmembrane and ubiquitin-like domain containing 2 | 17 | -0,3 | 4,5E-04 | 3,1E-02 |
| A_23_P501372 | NM_139162 | 125170 | SMCR7 | Smith-Magenis syndrome chromosome region, candidate 7 | 17 | -0,5 | 4,6E-04 | 3,2E-02 |
| A_23_P215517 | BC009555 | 55975 | KLHL7 | kelch-like 7 (Drosophila) | 7 | 0,4 | 4,6E-04 | 3,2E-02 |
| A_23_P91001 | NM_019048 | 54529 | ASNSD1 | asparagine synthetase domain containing 1 | 2 | 0,5 | 4,6E-04 | 3,2E-02 |
| A_23_P157600 | NM_015214 | 23259 | DDHD2 | DDHD domain containing 2 | 8 | 0,2 | 4,6E-04 | 3,2E-02 |
| A_23_P201386 | NM_012137 | 23576 | DDAH1 | dimethylarginine dimethylaminohydrolase 1 | 1 | 0,9 | 4,6E-04 | 3,2E-02 |
| A_23_P127964 | NM_199418 | 5547 | PRCP | prolylcarboxypeptidase (angiotensinase C) | 11 | 0,6 | 4,6E-04 | 3,2E-02 |
| A_23_P256542 | NM_014367 | 26355 | FAM162A | family with sequence similarity 162, member A | 3 | 0,9 | 4,6E-04 | 3,2E-02 |
| A_23_P97860 | NM_000235 | 3988 | LIPA | lipase A, lysosomal acid, cholesterol esterase | 10 | 0,6 | 4,6E-04 | 3,2E-02 |
| A_24_P376707 | NM_004494 | 3068 | HDGF | hepatoma-derived growth factor | 1 | -0,8 | 4,6E-04 | 3,2E-02 |
| A_24_P278747 | NM_001759 | 894 | CCND2 | cyclin D2 | 12 | -0,7 | 4,6E-04 | 3,2E-02 |
| A_23_P169978 | NM_020747 | 57507 | ZNF608 | zinc finger protein 608 | 5 | -0,2 | 4,7E-04 | 3,2E-02 |
| A_23_P110122 | NM_004354 | 901 | CCNG2 | cyclin G2 | 4 | -0,5 | 4,7E-04 | 3,2E-02 |
| A_24_P59569 | BC000922 | 400684 | LOC400684 | hypothetical LOC400684 | 19 | 0,1 | 4,7E-04 | 3,2E-02 |
| A_23_P215787 | NM_012257 | 26959 | HBP1 | HMG-box transcription factor 1 | 7 | -0,2 | 4,7E-04 | 3,2E-02 |
| A_23_P386 | NM_018125 | 55160 | ARHGEF10L | Rho guanine nucleotide exchange factor (GEF) 10-like | 1 | -0,3 | 4,7E-04 | 3,2E-02 |
| A_23_P218988 | NM_022902 | 64924 | SLC30A5 | solute carrier family 30 (zinc transporter), member 5 | 5 | 0,4 | 4,7E-04 | 3,2E-02 |
| A_24_P107257 | NM_018362 | 55327 | LIN7C | lin-7 homolog C (C. elegans) | 11 | 0,2 | 4,7E-04 | 3,2E-02 |
| A_23_P216132 | NM_017778 | 54904 | WHSC1L1 | Wolf-Hirschhorn syndrome candidate 1-like 1 | 8 | -0,3 | 4,7E-04 | 3,2E-02 |
| A_23_P390044 | NM_002359 | 4097 | MAFG | v-maf musculoaponeurotic fibrosarcoma oncogene homolog G (avian) | 17 | -0,7 | 4,8E-04 | 3,2E-02 |
| A_23_P433838 | NM_020774 | 57534 | MIB1 | mindbomb homolog 1 (Drosophila) | 18 | -0,1 | 4,8E-04 | 3,2E-02 |
| A_23_P308519 | NM_004252 | 9368 | SLC9A3R1 | solute carrier family 9 (sodium/hydrogen exchanger), member 3 regulator 1 | 17 | 0,6 | 4,8E-04 | 3,2E-02 |
| A_24_P265135 | NM_014671 | 9690 | UBE3C | ubiquitin protein ligase E3C | 7 | 0,1 | 4,8E-04 | 3,2E-02 |
| A_23_P60248 | NM_003329 | 7295 | TXN | thioredoxin | 9 | 0,9 | 4,8E-04 | 3,2E-02 |
| A_23_P29504 | THC2500665 | 205564 | SENP5 | SUMO1/sentrin specific peptidase 5 | 3 | 0,1 | 4,9E-04 | 3,2E-02 |
| A_23_P346265 | NM_024312 | 79158 | GNPTAB | N-acetylglucosamine-1-phosphate transferase, alpha and beta subunits | 12 | -0,1 | 4,9E-04 | 3,2E-02 |
| A_24_P350576 | AB011123 | 23043 | TNIK | TRAF2 and NCK interacting kinase | 3 | 0,1 | 4,9E-04 | 3,2E-02 |
| A_24_P270460 | NM_005532 | 3429 | IFI27 | interferon, alpha-inducible protein 27 | 14 | 0,1 | 4,9E-04 | 3,2E-02 |
| A_23_P91590 | NM_002882 | 5902 | RANBP1 | RAN binding protein 1 | 22 | 1,4 | 4,9E-04 | 3,2E-02 |
| A_24_P51279 | AL833832 | 57585 | CRAMP1L | Crm, cramped-like (Drosophila) | 16 | 0,0 | 4,9E-04 | 3,2E-02 |
| A_23_P67708 | NM_003200 | 6929 | TCF3 | transcription factor 3 (E2A immunoglobulin enhancer binding factors E12/E47) | 19 | 0,9 | 4,9E-04 | 3,2E-02 |
| A_23_P2366 | NM_199040 | 11163 | NUDT4 | nudix (nucleoside diphosphate linked moiety X)-type motif 4 | 12 | 0,3 | 5,0E-04 | 3,2E-02 |
| A_23_P81121 | NM_005033 | 5393 | EXOSC9 | exosome component 9 | 4 | 0,6 | 5,0E-04 | 3,2E-02 |
| A_23_P14482 | NM_016039 | 51637 | C14orf166 | chromosome 14 open reading frame 166 | 14 | 0,7 | 5,0E-04 | 3,2E-02 |
| A_24_P929083 | THC2528386 | 100505881 | LOC100505881 | hypothetical LOC100505881 | 7 | 0,4 | 5,0E-04 | 3,2E-02 |
| A_23_P151426 | NM_002015 | 2308 | FOXO1 | forkhead box O1 | 13 | -0,7 | 5,0E-04 | 3,2E-02 |
| A_24_P816384 | ENST00000339094 | 388165 | UBE2Q2P1 | ubiquitin-conjugating enzyme E2Q family member 2 pseudogene 1 | 15 | -0,2 | 5,0E-04 | 3,2E-02 |
| A_32_P31771 | NM_030650 | 80856 | KIAA1715 | KIAA1715 | 2 | 0,1 | 5,1E-04 | 3,3E-02 |
| A_23_P20728 | NM_003086 | 6621 | SNAPC4 | small nuclear RNA activating complex, polypeptide 4, 190kDa | 9 | -0,3 | 5,1E-04 | 3,3E-02 |
| A_23_P252721 | NM_182643 | 10395 | DLC1 | deleted in liver cancer 1 | 8 | 1,0 | 5,1E-04 | 3,3E-02 |
| A_23_P254379 | NM_006620 | 10767 | HBS1L | HBS1-like (S. cerevisiae) | 6 | 0,3 | 5,1E-04 | 3,3E-02 |
| A_24_P840688 | NM_170606 | 58508 | MLL3 | myeloid/lymphoid or mixed-lineage leukemia 3 | 7 | -0,1 | 5,2E-04 | 3,3E-02 |
| A_23_P3042 | NM_006246 | 5529 | PPP2R5E | protein phosphatase 2, regulatory subunit B', epsilon isoform | 14 | 0,4 | 5,2E-04 | 3,3E-02 |
| A_23_P202484 | NM_032772 | 84858 | ZNF503 | zinc finger protein 503 | 10 | -0,4 | 5,2E-04 | 3,3E-02 |
| A_23_P415510 | NM_005558 | 3898 | LAD1 | ladinin 1 | 1 | -0,9 | 5,2E-04 | 3,3E-02 |
| A_23_P52147 | NM_001079515 | 6905 | TBCE | tubulin folding cofactor E | 1 | 0,6 | 5,2E-04 | 3,3E-02 |
| A_23_P36408 | NM_022916 | 65082 | VPS33A | vacuolar protein sorting 33 homolog A (S. cerevisiae) | 12 | 0,2 | 5,2E-04 | 3,3E-02 |
| A_23_P258272 | NM_001039707 | 10807 | SDCCAG3 | serologically defined colon cancer antigen 3 | 9 | 0,4 | 5,2E-04 | 3,3E-02 |
| A_24_P7211 | NM_033551 | 23367 | LARP1 | La ribonucleoprotein domain family, member 1 | 5 | -0,1 | 5,2E-04 | 3,3E-02 |
| A_23_P383986 | NM_015892 | 51363 | CHST15 | carbohydrate (N-acetylgalactosamine 4-sulfate 6-O) sulfotransferase 15 | 10 | -0,6 | 5,2E-04 | 3,3E-02 |
| A_24_P318544 | AB209463 | 1453 | CSNK1D | casein kinase 1, delta | 17 | -0,5 | 5,3E-04 | 3,3E-02 |
| A_24_P125067 | NM_013302 | 29904 | EEF2K | eukaryotic elongation factor-2 kinase | 16 | 0,1 | 5,3E-04 | 3,3E-02 |
| A_32_P141664 | NM_002586 | 5089 | PBX2 | pre-B-cell leukemia homeobox 2 | 6 | -0,1 | 5,3E-04 | 3,3E-02 |
| A_23_P429491 | NM_145018 | 220042 | C11orf82 | chromosome 11 open reading frame 82 | 11 | 0,3 | 5,3E-04 | 3,3E-02 |
| A_23_P99837 | NM_017437 | 53981 | CPSF2 | cleavage and polyadenylation specific factor 2, 100kDa | 14 | 0,4 | 5,4E-04 | 3,3E-02 |
| A_24_P76740 | NM_032449 | 200014 | CC2D1B | coiled-coil and C2 domain containing 1B | 1 | -0,2 | 5,4E-04 | 3,3E-02 |
| A_24_P320645 | NM_025154 | 23353 | SUN1 | Sad1 and UNC84 domain containing 1 | 7 | -0,1 | 5,4E-04 | 3,3E-02 |
| A_23_P430120 | NM_001430 | 2034 | EPAS1 | endothelial PAS domain protein 1 | 2 | -0,1 | 5,4E-04 | 3,3E-02 |
| A_23_P329353 | NM_015463 | 25927 | CNRIP1 | cannabinoid receptor interacting protein 1 | 2 | 0,7 | 5,4E-04 | 3,3E-02 |
| A_23_P382775 | NM_014417 | 27113 | BBC3 | BCL2 binding component 3 | 19 | -1,1 | 5,5E-04 | 3,4E-02 |
| A_23_P138635 | NM_004052 | 664 | BNIP3 | BCL2/adenovirus E1B 19kDa interacting protein 3 | 10 | 1,3 | 5,5E-04 | 3,4E-02 |
| A_23_P205828 | NM_003257 | 7082 | TJP1 | tight junction protein 1 (zona occludens 1) | 15 | 0,4 | 5,5E-04 | 3,4E-02 |
| A_32_P24223 | NM_015355 | 23512 | SUZ12 | suppressor of zeste 12 homolog (Drosophila) | 17 | -0,3 | 5,5E-04 | 3,4E-02 |
| A_23_P11564 | NM_014774 | 9813 | KIAA0494 | KIAA0494 | 1 | -0,4 | 5,6E-04 | 3,4E-02 |
| A_23_P144378 | NM_032217 | 26057 | ANKRD17 | ankyrin repeat domain 17 | 4 | -0,7 | 5,6E-04 | 3,4E-02 |
| A_23_P258190 | NM_001628 | 231 | AKR1B1 | aldo-keto reductase family 1, member B1 (aldose reductase) | 7 | -1,9 | 5,6E-04 | 3,4E-02 |
| A_23_P374389 | NM_138499 | 170394 | PWWP2B | PWWP domain containing 2B | 10 | -0,4 | 5,6E-04 | 3,4E-02 |
| A_24_P74371 | NM_000308 | 5476 | CTSA | cathepsin A | 20 | -0,5 | 5,6E-04 | 3,4E-02 |
| A_32_P171923 | THC2512199 | 346389 | MACC1 | metastasis associated in colon cancer 1 | 7 | -0,3 | 5,7E-04 | 3,4E-02 |
| A_23_P357811 | NM_021038 | 4154 | MBNL1 | muscleblind-like (Drosophila) | 3 | 0,8 | 5,7E-04 | 3,4E-02 |
| A_23_P1962 | NM_004585 | 5920 | RARRES3 | retinoic acid receptor responder (tazarotene induced) 3 | 11 | -0,8 | 5,7E-04 | 3,4E-02 |
| A_23_P50674 | NM_001031735 | 113177 | IZUMO4 | IZUMO family member 4 | 19 | -0,1 | 5,7E-04 | 3,4E-02 |
| A_32_P141013 | ENST00000326261 | 440073 | IQSEC3 | IQ motif and Sec7 domain 3 | 12 | 0,0 | 5,7E-04 | 3,4E-02 |
| A_23_P79692 | NM_005759 | 10152 | ABI2 | abl-interactor 2 | 2 | 0,6 | 5,7E-04 | 3,4E-02 |
| A_23_P1043 | NM_018265 | 55765 | C1orf106 | chromosome 1 open reading frame 106 | 1 | -0,8 | 5,7E-04 | 3,4E-02 |
| A_23_P380998 | NM_015361 | 23518 | R3HDM1 | R3H domain containing 1 | 2 | 0,4 | 5,7E-04 | 3,4E-02 |
| A_23_P370635 | NM_138799 | 129642 | MBOAT2 | membrane bound O-acyltransferase domain containing 2 | 2 | 0,4 | 5,7E-04 | 3,4E-02 |
| A_23_P253561 | NM_024331 | 79183 | TTPAL | tocopherol (alpha) transfer protein-like | 20 | 0,4 | 5,7E-04 | 3,4E-02 |
| A_23_P6546 | NM_001008658 | 85378 | TUBGCP6 | tubulin, gamma complex associated protein 6 | 22 | -0,2 | 5,8E-04 | 3,4E-02 |
| A_23_P163506 | NM_000101 | 1535 | CYBA | cytochrome b-245, alpha polypeptide | 16 | -1,1 | 5,8E-04 | 3,4E-02 |
| A_32_P6408 | AL390175 | 54753 | ZNF853 | zinc finger protein 853 | 7 | -0,3 | 5,8E-04 | 3,4E-02 |
| A_23_P253350 | NM_020130 | 56892 | C8orf4 | chromosome 8 open reading frame 4 | 8 | -0,6 | 5,8E-04 | 3,4E-02 |
| A_23_P7727 | NM_001884 | 1404 | HAPLN1 | hyaluronan and proteoglycan link protein 1 | 5 | 1,6 | 5,8E-04 | 3,4E-02 |
| A_23_P39910 | NM_001008215 | 493753 | COA5 | cytochrome C oxidase assembly factor 5 | 2 | -0,2 | 5,8E-04 | 3,4E-02 |
| A_24_P320284 | NM_000791 | 1719 | DHFR | dihydrofolate reductase | 5 | 0,4 | 5,8E-04 | 3,4E-02 |
| A_23_P68155 | NM_022168 | 64135 | IFIH1 | interferon induced with helicase C domain 1 | 2 | -0,3 | 5,8E-04 | 3,4E-02 |
| A_23_P112251 | NM_001017998 | 2790 | GNG10 | guanine nucleotide binding protein (G protein), gamma 10 | 9 | 0,8 | 5,8E-04 | 3,4E-02 |
| A_32_P22501 | ENST00000313807 | 645212 | LOC645212 | hypothetical LOC645212 | 15 | -0,1 | 5,8E-04 | 3,4E-02 |
| A_23_P339095 | NM_178313 | 6711 | SPTBN1 | spectrin, beta, non-erythrocytic 1 | 2 | -0,3 | 5,8E-04 | 3,4E-02 |
| A_23_P314120 | NM_005198 | 1120 | CHKB | choline kinase beta | 22 | -0,5 | 5,9E-04 | 3,4E-02 |
| A_24_P249072 | NM_181531 | 10385 | BTN2A2 | butyrophilin, subfamily 2, member A2 | 6 | -0,3 | 5,9E-04 | 3,4E-02 |
| A_23_P40078 | NM_003400 | 7514 | XPO1 | exportin 1 (CRM1 homolog, yeast) | 2 | 0,5 | 5,9E-04 | 3,4E-02 |
| A_23_P300770 | NM_174916 | 197131 | UBR1 | ubiquitin protein ligase E3 component n-recognin 1 | 15 | 0,1 | 5,9E-04 | 3,4E-02 |
| A_23_P153441 | NM_013312 | 29911 | HOOK2 | hook homolog 2 (Drosophila) | 19 | -0,4 | 5,9E-04 | 3,4E-02 |
| A_23_P351667 | NM_003812 | 8745 | ADAM23 | ADAM metallopeptidase domain 23 | 2 | 0,3 | 5,9E-04 | 3,4E-02 |
| A_23_P93988 | NM_005435 | 7984 | ARHGEF5 | Rho guanine nucleotide exchange factor (GEF) 5 | 7 | -0,4 | 5,9E-04 | 3,4E-02 |
| A_23_P365874 | NM_018313 | 55193 | PBRM1 | polybromo 1 | 3 | 0,1 | 5,9E-04 | 3,4E-02 |
| A_24_P410399 | NM_032165 | 84125 | LRRIQ1 | leucine-rich repeats and IQ motif containing 1 | 12 | 0,0 | 6,0E-04 | 3,4E-02 |
| A_24_P91991 | NM_178557 | 339983 | NAT8L | N-acetyltransferase 8-like (GCN5-related, putative) | 4 | -0,1 | 6,0E-04 | 3,4E-02 |
| A_32_P149251 | NM_152686 | 202052 | DNAJC18 | DnaJ (Hsp40) homolog, subfamily C, member 18 | 5 | 0,2 | 6,0E-04 | 3,4E-02 |
| A_23_P418015 | NM_014268 | 10982 | MAPRE2 | microtubule-associated protein, RP/EB family, member 2 | 18 | 0,2 | 6,0E-04 | 3,4E-02 |
| A_23_P26024 | NM_032413 | 84419 | C15orf48 | chromosome 15 open reading frame 48 | 15 | -1,7 | 6,0E-04 | 3,5E-02 |
| A_23_P250948 | NM_001003694 | 7862 | BRPF1 | bromodomain and PHD finger containing, 1 | 3 | -0,1 | 6,0E-04 | 3,5E-02 |
| A_24_P158314 | NM_032293 | 84253 | GARNL3 | GTPase activating Rap/RanGAP domain-like 3 | 9 | -0,1 | 6,0E-04 | 3,5E-02 |
| A_23_P154345 | NM_014362 | 26275 | HIBCH | 3-hydroxyisobutyryl-CoA hydrolase | 2 | 0,5 | 6,1E-04 | 3,5E-02 |
| A_23_P109269 | NM_005560 | 3911 | LAMA5 | laminin, alpha 5 | 20 | -0,7 | 6,1E-04 | 3,5E-02 |
| A_23_P46141 | NM_004079 | 1520 | CTSS | cathepsin S | 1 | -0,1 | 6,1E-04 | 3,5E-02 |
| A_23_P61688 | NM_006598 | 10723 | SLC12A7 | solute carrier family 12 (potassium/chloride transporters), member 7 | 5 | -1,1 | 6,1E-04 | 3,5E-02 |
| A_23_P82509 | NM_021167 | 57798 | GATAD1 | GATA zinc finger domain containing 1 | 7 | -0,3 | 6,1E-04 | 3,5E-02 |
| A_23_P142724 | NM_000998 | 6168 | RPL37A | ribosomal protein L37a | 2 | -1,8 | 6,2E-04 | 3,5E-02 |
| A_24_P49190 | NM_181655 | 284018 | C17orf58 | chromosome 17 open reading frame 58 | 17 | 0,1 | 6,2E-04 | 3,5E-02 |
| A_24_P11436 | NM_017904 | 55001 | TTC22 | tetratricopeptide repeat domain 22 | 1 | 0,0 | 6,2E-04 | 3,5E-02 |
| A_23_P132936 | NM_021928 | 60559 | SPCS3 | signal peptidase complex subunit 3 homolog (S. cerevisiae) | 4 | 1,0 | 6,2E-04 | 3,5E-02 |
| A_23_P72651 | NM_001077693 | 641700 | ECSCR | endothelial cell-specific chemotaxis regulator | 5 | 0,1 | 6,3E-04 | 3,5E-02 |
| A_23_P213620 | NM_004576 | 5521 | PPP2R2B | protein phosphatase 2, regulatory subunit B, beta | 5 | 1,1 | 6,3E-04 | 3,6E-02 |
| A_24_P70002 | ENST00000382592 | 26524 | LATS2 | LATS, large tumor suppressor, homolog 2 (Drosophila) | 13 | -0,3 | 6,4E-04 | 3,6E-02 |
| A_23_P68327 | NM_080667 | 112942 | CCDC104 | coiled-coil domain containing 104 | 2 | 0,5 | 6,4E-04 | 3,6E-02 |
| A_23_P22378 | NM_003108 | 6664 | SOX11 | SRY (sex determining region Y)-box 11 | 2 | 0,2 | 6,4E-04 | 3,6E-02 |
| A_23_P109643 | NM_172027 | 80325 | ABTB1 | ankyrin repeat and BTB (POZ) domain containing 1 | 3 | -0,2 | 6,4E-04 | 3,6E-02 |
| A_23_P302709 | NM_005126 | 9975 | NR1D2 | nuclear receptor subfamily 1, group D, member 2 | 3 | -0,1 | 6,4E-04 | 3,6E-02 |
| A_23_P98605 | NM_015853 | 51035 | UBXN1 | UBX domain protein 1 | 11 | -0,5 | 6,4E-04 | 3,6E-02 |
| A_23_P91919 | NM_014445 | 27230 | SERP1 | stress-associated endoplasmic reticulum protein 1 | 3 | 0,4 | 6,4E-04 | 3,6E-02 |
| A_23_P214907 | NM_015440 | 25902 | MTHFD1L | methylenetetrahydrofolate dehydrogenase (NADP+ dependent) 1-like | 6 | 0,2 | 6,5E-04 | 3,6E-02 |
| A_24_P251764 | NM_002090 | 2921 | CXCL3 | chemokine (C-X-C motif) ligand 3 | 4 | -0,6 | 6,5E-04 | 3,6E-02 |
| A_23_P129614 | NM_007006 | 11051 | NUDT21 | nudix (nucleoside diphosphate linked moiety X)-type motif 21 | 16 | 0,8 | 6,5E-04 | 3,6E-02 |
| A_23_P128554 | NM_032565 | 84650 | EBPL | emopamil binding protein-like | 13 | 0,9 | 6,6E-04 | 3,6E-02 |
| A_23_P3450 | NM_014444 | 27229 | TUBGCP4 | tubulin, gamma complex associated protein 4 | 15 | 0,1 | 6,6E-04 | 3,6E-02 |
| A_23_P60130 | NM_052886 | 114569 | MAL2 | mal, T-cell differentiation protein 2 (gene/pseudogene) | 8 | -1,2 | 6,6E-04 | 3,6E-02 |
| A_24_P354488 | NM_014435 | 27163 | NAAA | N-acylethanolamine acid amidase | 4 | 0,3 | 6,6E-04 | 3,6E-02 |
| A_23_P83493 | NM_016033 | 51115 | FAM82B | family with sequence similarity 82, member B | 8 | 0,3 | 6,6E-04 | 3,6E-02 |
| A_23_P502710 | NM_152237 | 10634 | GAS2L1 | growth arrest-specific 2 like 1 | 22 | 0,5 | 6,6E-04 | 3,6E-02 |
| A_23_P52986 | NM_152718 | 220001 | VWCE | von Willebrand factor C and EGF domains | 11 | -0,8 | 6,6E-04 | 3,6E-02 |
| A_23_P20732 | NM_012204 | 9329 | GTF3C4 | general transcription factor IIIC, polypeptide 4, 90kDa | 9 | 0,5 | 6,7E-04 | 3,6E-02 |
| A_23_P67299 | NM_020812 | 57572 | DOCK6 | dedicator of cytokinesis 6 | 19 | -0,2 | 6,7E-04 | 3,6E-02 |
| A_23_P553 | NM_025150 | 80222 | TARS2 | threonyl-tRNA synthetase 2, mitochondrial (putative) | 1 | -0,3 | 6,7E-04 | 3,7E-02 |
| A_24_P342127 | NM_014997 | 23008 | KLHDC10 | kelch domain containing 10 | 7 | 0,1 | 6,8E-04 | 3,7E-02 |
| A_23_P383688 | NM_020745 | 57505 | AARS2 | alanyl-tRNA synthetase 2, mitochondrial (putative) | 6 | 0,2 | 6,8E-04 | 3,7E-02 |
| A_24_P261083 | NM_004337 | 734 | OSGIN2 | oxidative stress induced growth inhibitor family member 2 | 8 | 0,4 | 6,8E-04 | 3,7E-02 |
| A_24_P339201 | NM_024042 | 79006 | METRN | meteorin, glial cell differentiation regulator | 16 | -0,9 | 6,8E-04 | 3,7E-02 |
| A_23_P436336 | NM_001004321 | 349114 | NCRNA00265 | non-protein coding RNA 265 | 7 | -0,1 | 6,8E-04 | 3,7E-02 |
| A_32_P122529 | NM_001032389 | 441272 | SPDYE3 | speedy homolog E3 (Xenopus laevis) | 7 | 0,0 | 6,9E-04 | 3,7E-02 |
| A_23_P210658 | NM_016045 | 51012 | SLMO2 | slowmo homolog 2 (Drosophila) | 20 | 0,6 | 6,9E-04 | 3,7E-02 |
| A_24_P231057 | NM_148894 | 259282 | BOD1L | biorientation of chromosomes in cell division 1-like | 4 | -0,1 | 6,9E-04 | 3,7E-02 |
| A_32_P173744 | CR603215 | 728776 | HMGN1P37 | high mobility group nucleosome binding domain 1 pseudogene 37 | X | 0,5 | 7,0E-04 | 3,7E-02 |
| A_24_P248240 | NM_152280 | 23208 | SYT11 | synaptotagmin XI | 1 | -0,2 | 7,0E-04 | 3,7E-02 |
| A_24_P347378 | NM_001629 | 241 | ALOX5AP | arachidonate 5-lipoxygenase-activating protein | 13 | -0,3 | 7,0E-04 | 3,7E-02 |
| A_23_P127533 | NM_032299 | 84259 | DCUN1D5 | DCN1, defective in cullin neddylation 1, domain containing 5 (S. cerevisiae) | 11 | 0,9 | 7,0E-04 | 3,7E-02 |
| A_23_P207517 | NM_002611 | 5164 | PDK2 | pyruvate dehydrogenase kinase, isozyme 2 | 17 | -0,1 | 7,0E-04 | 3,7E-02 |
| A_23_P350107 | NM_030961 | 81844 | TRIM56 | tripartite motif containing 56 | 7 | -0,3 | 7,0E-04 | 3,7E-02 |
| A_32_P196263 | NM_182920 | 56999 | ADAMTS9 | ADAM metallopeptidase with thrombospondin type 1 motif, 9 | 3 | -0,7 | 7,0E-04 | 3,7E-02 |
| A_32_P193378 | AK055370 | 26108 | PYGO1 | pygopus homolog 1 (Drosophila) | 15 | -0,1 | 7,1E-04 | 3,8E-02 |
| A_23_P106532 | NM_130468 | 113189 | CHST14 | carbohydrate (N-acetylgalactosamine 4-0) sulfotransferase 14 | 15 | -0,4 | 7,1E-04 | 3,8E-02 |
| A_24_P58054 | NM_015266 | 23315 | SLC9A8 | solute carrier family 9 (sodium/hydrogen exchanger), member 8 | 20 | -0,1 | 7,1E-04 | 3,8E-02 |
| A_32_P49116 | CR617865 | 100510649 | LOC100510649 | hypothetical LOC100510649 | 13 | -0,1 | 7,1E-04 | 3,8E-02 |
| A_23_P213908 | NM_032177 | 51808 | PHAX | phosphorylated adaptor for RNA export | 5 | 0,6 | 7,1E-04 | 3,8E-02 |
| A_23_P168771 | NM_020879 | 57639 | CCDC146 | coiled-coil domain containing 146 | 7 | -0,1 | 7,2E-04 | 3,8E-02 |
| A_24_P187954 | NM_213654 | 25852 | ARMC8 | armadillo repeat containing 8 | 3 | 0,2 | 7,2E-04 | 3,8E-02 |
| A_24_P917123 | NM_013262 | 29116 | MYLIP | myosin regulatory light chain interacting protein | 6 | -0,1 | 7,2E-04 | 3,8E-02 |
| A_23_P90612 | NM_005915 | 4175 | MCM6 | minichromosome maintenance complex component 6 | 2 | 1,0 | 7,2E-04 | 3,8E-02 |
| A_23_P134517 | NM_033224 | 5814 | PURB | purine-rich element binding protein B | 7 | 0,5 | 7,2E-04 | 3,8E-02 |
| A_23_P89931 | NM_022752 | 64763 | ZNF574 | zinc finger protein 574 | 19 | -0,1 | 7,2E-04 | 3,8E-02 |
| A_24_P342312 | AB037723 | 26011 | ODZ4 | odz, odd Oz/ten-m homolog 4 (Drosophila) | 11 | -0,1 | 7,2E-04 | 3,8E-02 |
| A_23_P74446 | NM_014663 | 9682 | KDM4A | lysine (K)-specific demethylase 4A | 1 | -0,4 | 7,2E-04 | 3,8E-02 |
| A_23_P259521 | NM_018268 | 55255 | WDR41 | WD repeat domain 41 | 5 | 0,3 | 7,2E-04 | 3,8E-02 |
| A_23_P333998 | AF090919 | 10721 | POLQ | polymerase (DNA directed), theta | 3 | 0,0 | 7,3E-04 | 3,8E-02 |
| A_32_P75284 | NM_032116 | 84056 | KATNAL1 | katanin p60 subunit A-like 1 | 13 | 0,9 | 7,3E-04 | 3,8E-02 |
| A_23_P24515 | NM_000019 | 38 | ACAT1 | acetyl-CoA acetyltransferase 1 | 11 | 0,6 | 7,3E-04 | 3,8E-02 |
| A_32_P147622 | BC004179 | 100289410 | MCF2L-AS1 | MCF2L antisense RNA 1 (non-protein coding) | 13 | -0,2 | 7,3E-04 | 3,8E-02 |
| A_23_P395609 | NM_147189 | 90362 | FAM110B | family with sequence similarity 110, member B | 8 | -0,3 | 7,3E-04 | 3,8E-02 |
| A_23_P306203 | NM_030754 | 6289 | SAA2 | serum amyloid A2 | 11 | -1,2 | 7,3E-04 | 3,8E-02 |
| A_24_P102043 | THC2575933 | 4666 | NACA | nascent polypeptide-associated complex alpha subunit | 12 | 0,1 | 7,4E-04 | 3,8E-02 |
| A_24_P150803 | NM_002773 | 5652 | PRSS8 | protease, serine, 8 | 16 | -0,2 | 7,4E-04 | 3,8E-02 |
| A_32_P218355 | ENST00000341865 | 647024 | C6orf132 | chromosome 6 open reading frame 132 | 6 | -0,4 | 7,4E-04 | 3,8E-02 |
| A_23_P115022 | NM_144626 | 128218 | TMEM125 | transmembrane protein 125 | 1 | -0,5 | 7,4E-04 | 3,8E-02 |
| A_32_P192033 | AK055918 | 403150 | FLJ31356 | hypothetical protein FLJ31356 | 2 | 0,1 | 7,4E-04 | 3,8E-02 |
| A_23_P167051 | AF216184 | 2121 | EVC | Ellis van Creveld syndrome | 4 | -0,3 | 7,4E-04 | 3,8E-02 |
| A_23_P114670 | NM_014448 | 27237 | ARHGEF16 | Rho guanine nucleotide exchange factor (GEF) 16 | 1 | -0,9 | 7,5E-04 | 3,8E-02 |
| A_23_P101407 | NM_000064 | 718 | C3 | complement component 3 | 19 | -1,5 | 7,5E-04 | 3,8E-02 |
| A_23_P384635 | NM_173543 | 199221 | DZIP1L | DAZ interacting protein 1-like | 3 | 0,1 | 7,5E-04 | 3,8E-02 |
| A_23_P141208 | NM_015510 | 25979 | DHRS7B | dehydrogenase/reductase (SDR family) member 7B | 17 | -0,3 | 7,5E-04 | 3,8E-02 |
| A_23_P122852 | NM_003078 | 6604 | SMARCD3 | SWI/SNF related, matrix associated, actin dependent regulator of chromatin, subfamily d, member 3 | 7 | 0,7 | 7,5E-04 | 3,8E-02 |
| A_32_P42075 | NM_198097 | 221960 | C7orf28B | chromosome 7 open reading frame 28B | 7 | 0,6 | 7,6E-04 | 3,8E-02 |
| A_24_P301146 | NM_005358 | 4008 | LMO7 | LIM domain 7 | 13 | 1,4 | 7,6E-04 | 3,8E-02 |
| A_23_P27677 | NM_001571 | 3661 | IRF3 | interferon regulatory factor 3 | 19 | -0,4 | 7,6E-04 | 3,8E-02 |
| A_23_P410746 | NM_014916 | 22853 | LMTK2 | lemur tyrosine kinase 2 | 7 | 0,0 | 7,6E-04 | 3,8E-02 |
| A_23_P60816 | NM_013374 | 10015 | PDCD6IP | programmed cell death 6 interacting protein | 3 | 0,6 | 7,6E-04 | 3,8E-02 |
| A_32_P192692 | NM_001014797 | 3778 | KCNMA1 | potassium large conductance calcium-activated channel, subfamily M, alpha member 1 | 10 | 0,3 | 7,7E-04 | 3,9E-02 |
| A_24_P6517 | NM_001029884 | 57480 | PLEKHG1 | pleckstrin homology domain containing, family G (with RhoGef domain) member 1 | 6 | -0,1 | 7,8E-04 | 3,9E-02 |
| A_24_P180654 | BC063666 | 64764 | CREB3L2 | cAMP responsive element binding protein 3-like 2 | 7 | 0,3 | 7,8E-04 | 3,9E-02 |
| A_23_P214111 | NM_022113 | 63971 | KIF13A | kinesin family member 13A | 6 | 0,1 | 7,8E-04 | 3,9E-02 |
| A_23_P29630 | NM_014041 | 28972 | SPCS1 | signal peptidase complex subunit 1 homolog (S. cerevisiae) | 3 | -0,7 | 7,8E-04 | 3,9E-02 |
| A_23_P413761 | NM_003017 | 6428 | SRSF3 | serine/arginine-rich splicing factor 3 | 6 | 0,8 | 7,8E-04 | 3,9E-02 |
| A_23_P66664 | NM_133439 | 6871 | TADA2A | transcriptional adaptor 2A | 17 | 0,0 | 7,8E-04 | 3,9E-02 |
| A_23_P323751 | NM_030919 | 81610 | FAM83D | family with sequence similarity 83, member D | 20 | 0,3 | 7,8E-04 | 3,9E-02 |
| A_23_P144796 | NM_003687 | 8572 | PDLIM4 | PDZ and LIM domain 4 | 5 | -1,2 | 7,9E-04 | 3,9E-02 |
| A_23_P3574 | NM_030819 | 81577 | GFOD2 | glucose-fructose oxidoreductase domain containing 2 | 16 | -0,3 | 7,9E-04 | 3,9E-02 |
| A_24_P386323 | NM_005833 | 10244 | RABEPK | Rab9 effector protein with kelch motifs | 9 | 0,4 | 7,9E-04 | 3,9E-02 |
| A_32_P440095 | NR_002936 | 222699 | TOB2P1 | transducer of ERBB2, 2 pseudogene 1 | 6 | 0,0 | 7,9E-04 | 3,9E-02 |
| A_24_P922808 | BC020640 | 51029 | PPPDE1 | PPPDE peptidase domain containing 1 | 1 | 0,1 | 7,9E-04 | 3,9E-02 |
| A_24_P586390 | AK123446 | 100133331 | LOC100133331 | hypothetical LOC100133331 | 1 | 0,0 | 7,9E-04 | 3,9E-02 |
| A_24_P401842 | AK074711 | 64399 | HHIP | hedgehog interacting protein | 4 | 0,3 | 7,9E-04 | 3,9E-02 |
| A_23_P435904 | ENST00000334827 | 84859 | LRCH3 | leucine-rich repeats and calponin homology (CH) domain containing 3 | 3 | 0,0 | 8,0E-04 | 3,9E-02 |
| A_24_P227069 | NM_020918 | 57678 | GPAM | glycerol-3-phosphate acyltransferase, mitochondrial | 10 | 0,2 | 8,0E-04 | 3,9E-02 |
| A_32_P206541 | AK128714 | 133418 | EMB | embigin | 5 | 0,4 | 8,1E-04 | 3,9E-02 |
| A_23_P404685 | NM_178348 | 353131 | LCE1A | late cornified envelope 1A | 1 | 0,3 | 8,1E-04 | 3,9E-02 |
| A_24_P116535 | NM_002428 | 4324 | MMP15 | matrix metallopeptidase 15 (membrane-inserted) | 16 | -0,8 | 8,1E-04 | 3,9E-02 |
| A_23_P254254 | NM_000199 | 6448 | SGSH | N-sulfoglucosamine sulfohydrolase | 17 | -0,8 | 8,1E-04 | 4,0E-02 |
| A_24_P110983 | ENST00000366539 | 10000 | AKT3 | v-akt murine thymoma viral oncogene homolog 3 (protein kinase B, gamma) | 1 | 0,4 | 8,1E-04 | 4,0E-02 |
| A_32_P226078 | NM_016178 | 51686 | OAZ3 | ornithine decarboxylase antizyme 3 | 1 | 0,0 | 8,1E-04 | 4,0E-02 |
| A_23_P206359 | NM_004360 | 999 | CDH1 | cadherin 1, type 1, E-cadherin (epithelial) | 16 | -0,8 | 8,1E-04 | 4,0E-02 |
| A_23_P107421 | NM_003258 | 7083 | TK1 | thymidine kinase 1, soluble | 17 | 2,3 | 8,2E-04 | 4,0E-02 |
| A_32_P68746 | NM_018225 | 55234 | SMU1 | smu-1 suppressor of mec-8 and unc-52 homolog (C. elegans) | 9 | 0,4 | 8,2E-04 | 4,0E-02 |
| A_23_P127175 | NM_020150 | 56681 | SAR1A | SAR1 homolog A (S. cerevisiae) | 10 | 0,9 | 8,3E-04 | 4,0E-02 |
| A_23_P395524 | NM_007062 | 11137 | PWP1 | PWP1 homolog (S. cerevisiae) | 12 | 0,5 | 8,3E-04 | 4,0E-02 |
| A_23_P207967 | NM_014772 | 9811 | CTIF | CBP80/20-dependent translation initiation factor | 18 | -0,3 | 8,4E-04 | 4,0E-02 |
| A_23_P143127 | NM_019063 | 27436 | EML4 | echinoderm microtubule associated protein like 4 | 2 | 0,7 | 8,4E-04 | 4,0E-02 |
| A_32_P118847 | BC044619 | 441094 | FLJ42709 | hypothetical LOC441094 | 5 | 0,1 | 8,4E-04 | 4,0E-02 |
| A_23_P100074 | NM_020371 | 57099 | AVEN | apoptosis, caspase activation inhibitor | 15 | 0,6 | 8,5E-04 | 4,1E-02 |
| A_23_P141447 | NM_001034836 | 201299 | RDM1 | RAD52 motif 1 | 17 | 0,1 | 8,5E-04 | 4,1E-02 |
| A_24_P410678 | NM_002227 | 3716 | JAK1 | Janus kinase 1 | 1 | -0,9 | 8,5E-04 | 4,1E-02 |
| A_23_P983 | NM_004905 | 9588 | PRDX6 | peroxiredoxin 6 | 1 | 0,7 | 8,5E-04 | 4,1E-02 |
| A_32_P313405 | NM_005559 | 284217 | LAMA1 | laminin, alpha 1 | 18 | 0,5 | 8,6E-04 | 4,1E-02 |
| A_23_P103775 | NM_032270 | 84230 | LRRC8C | leucine rich repeat containing 8 family, member C | 1 | 0,0 | 8,6E-04 | 4,1E-02 |
| A_24_P19410 | NM_175709 | 23492 | CBX7 | chromobox homolog 7 | 22 | -0,2 | 8,6E-04 | 4,1E-02 |
| A_23_P386411 | NM_005038 | 5481 | PPID | peptidylprolyl isomerase D | 4 | 0,3 | 8,6E-04 | 4,1E-02 |
| A_23_P386942 | NM_145173 | 148252 | DIRAS1 | DIRAS family, GTP-binding RAS-like 1 | 19 | 0,2 | 8,6E-04 | 4,1E-02 |
| A_32_P208403 | NM_053064 | 54331 | GNG2 | guanine nucleotide binding protein (G protein), gamma 2 | 14 | 0,1 | 8,7E-04 | 4,1E-02 |
| A_23_P100056 | NM_194272 | 348093 | RBPMS2 | RNA binding protein with multiple splicing 2 | 15 | 0,0 | 8,7E-04 | 4,1E-02 |
| A_23_P71037 | NM_000600 | 3569 | IL6 | interleukin 6 (interferon, beta 2) | 7 | -2,1 | 8,7E-04 | 4,1E-02 |
| A_23_P42386 | NM_000735 | 1081 | CGA | glycoprotein hormones, alpha polypeptide | 6 | 0,0 | 8,7E-04 | 4,1E-02 |
| A_23_P315286 | NM_138774 | 91300 | C19orf22 | chromosome 19 open reading frame 22 | 19 | -0,2 | 8,7E-04 | 4,1E-02 |
| A_23_P122197 | NM_031966 | 891 | CCNB1 | cyclin B1 | 5 | 1,4 | 8,7E-04 | 4,1E-02 |
| A_23_P1014 | ENST00000367003 | 84791 | C1orf97 | chromosome 1 open reading frame 97 | 1 | 0,5 | 8,7E-04 | 4,1E-02 |
| A_23_P125748 | NM_032441 | 84460 | ZMAT1 | zinc finger, matrin-type 1 | X | -0,1 | 8,8E-04 | 4,1E-02 |
| A_23_P71598 | NM_003829 | 8777 | MPDZ | multiple PDZ domain protein | 9 | -0,1 | 8,8E-04 | 4,1E-02 |
| A_23_P150092 | NM_012247 | 22929 | SEPHS1 | selenophosphate synthetase 1 | 10 | 0,8 | 8,8E-04 | 4,1E-02 |
| A_23_P52531 | NM_152644 | 196792 | FAM24B | family with sequence similarity 24, member B | 10 | -0,2 | 8,8E-04 | 4,1E-02 |
| A_24_P84822 | NM_001033523 | 728411 | GUSBP1 | glucuronidase, beta pseudogene 1 | 5 | -0,2 | 8,8E-04 | 4,1E-02 |
| A_23_P200489 | NM_014698 | 9725 | TMEM63A | transmembrane protein 63A | 1 | -0,1 | 8,9E-04 | 4,1E-02 |
| A_24_P100605 | NM_020343 | 57186 | RALGAPA2 | Ral GTPase activating protein, alpha subunit 2 (catalytic) | 20 | -0,1 | 8,9E-04 | 4,1E-02 |
| A_23_P97442 | NM_032125 | 84065 | TMEM222 | transmembrane protein 222 | 1 | -0,3 | 8,9E-04 | 4,1E-02 |
| A_23_P106241 | NM_004239 | 9321 | TRIP11 | thyroid hormone receptor interactor 11 | 14 | 0,6 | 8,9E-04 | 4,1E-02 |
| A_23_P43034 | NM_018091 | 55140 | ELP3 | elongation protein 3 homolog (S. cerevisiae) | 8 | 0,3 | 8,9E-04 | 4,1E-02 |
| A_23_P39481 | NM_019112 | 10347 | ABCA7 | ATP-binding cassette, sub-family A (ABC1), member 7 | 19 | -0,2 | 8,9E-04 | 4,1E-02 |
| A_32_P119248 | NM_207305 | 2298 | FOXD4 | forkhead box D4 | 9 | 0,0 | 8,9E-04 | 4,1E-02 |
| A_23_P148015 | NM_004655 | 8313 | AXIN2 | axin 2 | 17 | -0,1 | 9,0E-04 | 4,1E-02 |
| A_23_P118815 | NM_001012271 | 332 | BIRC5 | baculoviral IAP repeat containing 5 | 17 | 2,0 | 9,0E-04 | 4,1E-02 |
| A_24_P406060 | NM_182757 | 255488 | RNF144B | ring finger protein 144B | 6 | -0,6 | 9,0E-04 | 4,1E-02 |
| A_24_P123011 | NM_006761 | 7531 | YWHAE | tyrosine 3-monooxygenase/tryptophan 5-monooxygenase activation protein, epsilon polypeptide | 17 | -0,1 | 9,0E-04 | 4,1E-02 |
| A_24_P138713 | NM_182922 | 55027 | HEATR3 | HEAT repeat containing 3 | 16 | 0,1 | 9,0E-04 | 4,1E-02 |
| A_23_P124035 | NM_032111 | 64928 | MRPL14 | mitochondrial ribosomal protein L14 | 6 | -0,4 | 9,1E-04 | 4,2E-02 |
| A_23_P44724 | NM_001321 | 1466 | CSRP2 | cysteine and glycine-rich protein 2 | 12 | 1,1 | 9,1E-04 | 4,2E-02 |
| A_23_P203900 | NM_005505 | 949 | SCARB1 | scavenger receptor class B, member 1 | 12 | 0,9 | 9,1E-04 | 4,2E-02 |
| A_23_P58443 | NM_020690 | 404734 | ANKHD1-EIF4EBP3 | ANKHD1-EIF4EBP3 readthrough | 5 | -0,2 | 9,1E-04 | 4,2E-02 |
| A_23_P150286 | NM_148976 | 5682 | PSMA1 | proteasome (prosome, macropain) subunit, alpha type, 1 | 11 | 0,8 | 9,2E-04 | 4,2E-02 |
| A_23_P50167 | NM_012319 | 25800 | SLC39A6 | solute carrier family 39 (zinc transporter), member 6 | 18 | -0,4 | 9,2E-04 | 4,2E-02 |
| A_24_P366082 | ENST00000252134 | 57553 | MICAL3 | microtubule associated monoxygenase, calponin and LIM domain containing 3 | 22 | -0,7 | 9,2E-04 | 4,2E-02 |
| A_24_P403244 | NM_013440 | 29990 | PILRB | paired immunoglobin-like type 2 receptor beta | 7 | -0,5 | 9,3E-04 | 4,2E-02 |
| A_23_P121356 | NM_020235 | 56987 | BBX | bobby sox homolog (Drosophila) | 3 | -0,4 | 9,3E-04 | 4,2E-02 |
| A_23_P50096 | NM_001071 | 7298 | TYMS | thymidylate synthetase | 18 | 1,6 | 9,3E-04 | 4,2E-02 |
| A_23_P410859 | ENST00000252744 | 57688 | ZSWIM6 | zinc finger, SWIM-type containing 6 | 5 | -0,2 | 9,3E-04 | 4,2E-02 |
| A_23_P69188 | NM_206831 | 285381 | DPH3 | DPH3, KTI11 homolog (S. cerevisiae) | 3 | 0,7 | 9,4E-04 | 4,2E-02 |
| A_23_P46539 | NM_032636 | 84722 | PSRC1 | proline/serine-rich coiled-coil 1 | 1 | 0,5 | 9,4E-04 | 4,2E-02 |
| A_24_P218056 | NM_015332 | 23386 | NUDCD3 | NudC domain containing 3 | 7 | -0,1 | 9,5E-04 | 4,2E-02 |
| A_24_P926972 | AK000923 | 26168 | SENP3 | SUMO1/sentrin/SMT3 specific peptidase 3 | 17 | -0,1 | 9,5E-04 | 4,2E-02 |
| A_23_P141606 | NM_000386 | 642 | BLMH | bleomycin hydrolase | 17 | 0,5 | 9,5E-04 | 4,2E-02 |
| A_23_P109171 | NM_001195 | 631 | BFSP1 | beaded filament structural protein 1, filensin | 20 | 0,5 | 9,5E-04 | 4,2E-02 |
| A_23_P117346 | NM_003861 | 8816 | DCAF5 | DDB1 and CUL4 associated factor 5 | 14 | -0,4 | 9,6E-04 | 4,3E-02 |
| A_24_P941625 | NM_021916 | 7621 | ZNF70 | zinc finger protein 70 | 22 | 0,1 | 9,6E-04 | 4,3E-02 |
| A_23_P310921 | NM_002589 | 5099 | PCDH7 | protocadherin 7 | 4 | 0,6 | 9,6E-04 | 4,3E-02 |
| A_24_P46689 | NM_015278 | 23328 | SASH1 | SAM and SH3 domain containing 1 | 6 | -0,4 | 9,6E-04 | 4,3E-02 |
| A_23_P57521 | NM_016091 | 51386 | EIF3L | eukaryotic translation initiation factor 3, subunit L | 22 | -0,5 | 9,6E-04 | 4,3E-02 |
| A_23_P43164 | NM_015170 | 23213 | SULF1 | sulfatase 1 | 8 | 2,1 | 9,6E-04 | 4,3E-02 |
| A_23_P155335 | NM_002662 | 5337 | PLD1 | phospholipase D1, phosphatidylcholine-specific | 3 | 0,3 | 9,7E-04 | 4,3E-02 |
| A_23_P214211 | NM_020320 | 57038 | RARS2 | arginyl-tRNA synthetase 2, mitochondrial | 6 | 0,4 | 9,7E-04 | 4,3E-02 |
| A_23_P8380 | NM_024033 | 78996 | C7orf49 | chromosome 7 open reading frame 49 | 7 | -0,4 | 9,7E-04 | 4,3E-02 |
| A_23_P154688 | NM_032034 | 83959 | SLC4A11 | solute carrier family 4, sodium borate transporter, member 11 | 20 | -0,4 | 9,7E-04 | 4,3E-02 |
| A_23_P117727 | NM_139242 | 123263 | MTFMT | mitochondrial methionyl-tRNA formyltransferase | 15 | 0,1 | 9,7E-04 | 4,3E-02 |
| A_24_P5856 | NM_003811 | 8744 | TNFSF9 | tumor necrosis factor (ligand) superfamily, member 9 | 19 | -0,1 | 9,7E-04 | 4,3E-02 |
| A_23_P404606 | NM_153607 | 153222 | C5orf41 | chromosome 5 open reading frame 41 | 5 | -0,3 | 9,7E-04 | 4,3E-02 |
| A_23_P49972 | NM_001254 | 990 | CDC6 | cell division cycle 6 homolog (S. cerevisiae) | 17 | 0,6 | 9,7E-04 | 4,3E-02 |
| A_23_P388433 | CR597270 | 401152 | C4orf3 | chromosome 4 open reading frame 3 | 4 | 0,8 | 9,8E-04 | 4,3E-02 |
| A_23_P169249 | NM_017585 | 11182 | SLC2A6 | solute carrier family 2 (facilitated glucose transporter), member 6 | 9 | -0,3 | 9,8E-04 | 4,3E-02 |
| A_23_P207476 | BC007524 | 9043 | SPAG9 | sperm associated antigen 9 | 17 | 0,0 | 9,8E-04 | 4,3E-02 |
| A_23_P396867 | NM_178582 | 81502 | HM13 | histocompatibility (minor) 13 | 20 | -0,1 | 9,9E-04 | 4,3E-02 |
| A_23_P71117 | NM_018843 | 55972 | SLC25A40 | solute carrier family 25, member 40 | 7 | 0,1 | 9,9E-04 | 4,3E-02 |
| A_23_P107903 | ENST00000380456 | 57139 | RGL3 | ral guanine nucleotide dissociation stimulator-like 3 | 19 | -0,2 | 9,9E-04 | 4,3E-02 |
| A_23_P42664 | NM_006304 | 7979 | SHFM1 | split hand/foot malformation (ectrodactyly) type 1 | 7 | 0,6 | 9,9E-04 | 4,3E-02 |
| A_24_P37441 | NM_002610 | 5163 | PDK1 | pyruvate dehydrogenase kinase, isozyme 1 | 2 | 0,6 | 9,9E-04 | 4,3E-02 |
| A_23_P164210 | NM_032258 | 84218 | TBC1D3F | TBC1 domain family, member 3F | 17 | -0,7 | 1,0E-03 | 4,3E-02 |
| A_32_P47754 | BC060766 | 144195 | SLC2A14 | solute carrier family 2 (facilitated glucose transporter), member 14 | 12 | 0,5 | 1,0E-03 | 4,3E-02 |
| A_23_P162540 | NM_031954 | 83892 | KCTD10 | potassium channel tetramerisation domain containing 10 | 12 | 0,4 | 1,0E-03 | 4,4E-02 |
| A_23_P7528 | NM_001387 | 1809 | DPYSL3 | dihydropyrimidinase-like 3 | 5 | 0,6 | 1,0E-03 | 4,4E-02 |
| A_23_P151710 | NM_000956 | 5732 | PTGER2 | prostaglandin E receptor 2 (subtype EP2), 53kDa | 14 | 0,2 | 1,0E-03 | 4,4E-02 |
| A_32_P114866 | BC040156 | 284570 | LOC284570 | hypothetical protein LOC284570 | 1 | -0,1 | 1,0E-03 | 4,4E-02 |
| A_23_P41796 | ENST00000264777 | 4214 | MAP3K1 | mitogen-activated protein kinase kinase kinase 1 | 5 | -0,1 | 1,0E-03 | 4,4E-02 |
| A_23_P368934 | AB046765 | 57666 | FBRSL1 | fibrosin-like 1 | 12 | -0,3 | 1,0E-03 | 4,4E-02 |
| A_23_P74349 | NM_145697 | 83540 | NUF2 | NUF2, NDC80 kinetochore complex component, homolog (S. cerevisiae) | 1 | 0,7 | 1,0E-03 | 4,4E-02 |
| A_23_P20384 | NM_014462 | 27257 | LSM1 | LSM1 homolog, U6 small nuclear RNA associated (S. cerevisiae) | 8 | 0,5 | 1,0E-03 | 4,4E-02 |
| A_23_P358995 | NM_015525 | 25998 | IBTK | inhibitor of Bruton agammaglobulinemia tyrosine kinase | 6 | 0,6 | 1,0E-03 | 4,4E-02 |
| A_23_P153236 | NM_014453 | 27243 | CHMP2A | chromatin modifying protein 2A | 19 | -0,5 | 1,0E-03 | 4,4E-02 |
| A_23_P132973 | NM_001042616 | 84992 | PIGY | phosphatidylinositol glycan anchor biosynthesis, class Y | 4 | 0,7 | 1,0E-03 | 4,4E-02 |
| A_23_P23630 | NM_030649 | 116983 | ACAP3 | ArfGAP with coiled-coil, ankyrin repeat and PH domains 3 | 1 | -0,1 | 1,0E-03 | 4,4E-02 |
| A_24_P601831 | NM_001037231 | 285735 | NCRNA00326 | non-protein coding RNA 326 | 6 | 0,0 | 1,0E-03 | 4,4E-02 |
| A_23_P253412 | NM_019051 | 54534 | MRPL50 | mitochondrial ribosomal protein L50 | 9 | 0,8 | 1,0E-03 | 4,4E-02 |
| A_23_P207106 | NM_000747 | 1140 | CHRNB1 | cholinergic receptor, nicotinic, beta 1 (muscle) | 17 | -0,1 | 1,0E-03 | 4,4E-02 |
| A_23_P314101 | NM_019601 | 56241 | SUSD2 | sushi domain containing 2 | 22 | 0,1 | 1,0E-03 | 4,4E-02 |
| A_23_P32615 | NM_002431 | 4331 | MNAT1 | menage a trois homolog 1, cyclin H assembly factor (Xenopus laevis) | 14 | 0,6 | 1,0E-03 | 4,4E-02 |
| A_23_P120488 | NM_024663 | 79716 | NPEPL1 | aminopeptidase-like 1 | 20 | -0,4 | 1,0E-03 | 4,4E-02 |
| A_24_P51061 | NM_080927 | 131566 | DCBLD2 | discoidin, CUB and LCCL domain containing 2 | 3 | -0,7 | 1,0E-03 | 4,4E-02 |
| A_32_P88817 | THC2554498 | 441251 | SPDYE7P | speedy homolog E7 (Xenopus laevis), pseudogene | 7 | 0,0 | 1,0E-03 | 4,4E-02 |
| A_23_P154025 | NM_003142 | 6741 | SSB | Sjogren syndrome antigen B (autoantigen La) | 2 | 0,7 | 1,0E-03 | 4,4E-02 |
| A_32_P53524 | BC092429 | 9423 | NTN1 | netrin 1 | 17 | -0,5 | 1,0E-03 | 4,4E-02 |
| A_24_P602507 | NM_001013619 | 123688 | AGPHD1 | aminoglycoside phosphotransferase domain containing 1 | 15 | 0,1 | 1,1E-03 | 4,4E-02 |
| A_23_P157416 | NM_032164 | 84124 | ZNF394 | zinc finger protein 394 | 7 | -0,2 | 1,1E-03 | 4,4E-02 |
| A_23_P79426 | NM_016289 | 51719 | CAB39 | calcium binding protein 39 | 2 | -0,3 | 1,1E-03 | 4,4E-02 |
| A_23_P33256 | NM_000174 | 2815 | GP9 | glycoprotein IX (platelet) | 3 | -0,3 | 1,1E-03 | 4,4E-02 |
| A_23_P216448 | NM_005596 | 4781 | NFIB | nuclear factor I/B | 9 | -0,2 | 1,1E-03 | 4,4E-02 |
| A_23_P502196 | NM_174855 | 3420 | IDH3B | isocitrate dehydrogenase 3 (NAD+) beta | 20 | -0,3 | 1,1E-03 | 4,4E-02 |
| A_23_P112726 | NM_002977 | 6335 | SCN9A | sodium channel, voltage-gated, type IX, alpha subunit | 2 | 0,4 | 1,1E-03 | 4,4E-02 |
| A_23_P152583 | NM_001042573 | 64772 | ENGASE | endo-beta-N-acetylglucosaminidase | 17 | -0,2 | 1,1E-03 | 4,5E-02 |
| A_24_P199905 | NM_006826 | 10971 | YWHAQ | tyrosine 3-monooxygenase/tryptophan 5-monooxygenase activation protein, theta polypeptide | 2 | 0,6 | 1,1E-03 | 4,5E-02 |
| A_23_P309701 | NM_002828 | 5771 | PTPN2 | protein tyrosine phosphatase, non-receptor type 2 | 18 | 0,1 | 1,1E-03 | 4,5E-02 |
| A_23_P153889 | NM_012088 | 25796 | PGLS | 6-phosphogluconolactonase | 19 | -0,7 | 1,1E-03 | 4,5E-02 |
| A_23_P58529 | NM_014473 | 27292 | DIMT1L | DIM1 dimethyladenosine transferase 1-like (S. cerevisiae) | 5 | 0,6 | 1,1E-03 | 4,5E-02 |
| A_32_P220938 | NM_006916 | 6120 | RPE | ribulose-5-phosphate-3-epimerase | 2 | 0,2 | 1,1E-03 | 4,5E-02 |
| A_24_P322354 | NM_001039535 | 220134 | SKA1 | spindle and kinetochore associated complex subunit 1 | 18 | 0,2 | 1,1E-03 | 4,5E-02 |
| A_32_P62342 | BC030023 | 283464 | GXYLT1 | glucoside xylosyltransferase 1 | 12 | 0,6 | 1,1E-03 | 4,5E-02 |
| A_23_P3627 | NM_003119 | 6687 | SPG7 | spastic paraplegia 7 (pure and complicated autosomal recessive) | 16 | -0,5 | 1,1E-03 | 4,5E-02 |
| A_23_P284 | NM_017847 | 54953 | C1orf27 | chromosome 1 open reading frame 27 | 1 | 0,2 | 1,1E-03 | 4,5E-02 |
| A_23_P168747 | NM_017760 | 54892 | NCAPG2 | non-SMC condensin II complex, subunit G2 | 7 | 0,5 | 1,1E-03 | 4,5E-02 |
| A_23_P165657 | NM_005415 | 6574 | SLC20A1 | solute carrier family 20 (phosphate transporter), member 1 | 2 | 1,0 | 1,1E-03 | 4,5E-02 |
| A_23_P413456 | AK128423 | 1153 | CIRBP | cold inducible RNA binding protein | 19 | -0,2 | 1,1E-03 | 4,6E-02 |
| A_32_P165611 | NM_015254 | 23303 | KIF13B | kinesin family member 13B | 8 | -0,2 | 1,1E-03 | 4,6E-02 |
| A_24_P205019 | NM_021061 | 58500 | ZNF250 | zinc finger protein 250 | 8 | -0,3 | 1,1E-03 | 4,6E-02 |
| A_24_P389491 | NM_016035 | 51117 | COQ4 | coenzyme Q4 homolog (S. cerevisiae) | 9 | -0,2 | 1,1E-03 | 4,6E-02 |
| A_23_P52161 | NM_030952 | 81788 | NUAK2 | NUAK family, SNF1-like kinase, 2 | 1 | -0,7 | 1,1E-03 | 4,6E-02 |
| A_24_P399230 | NM_175575 | 124857 | WFIKKN2 | WAP, follistatin/kazal, immunoglobulin, kunitz and netrin domain containing 2 | 17 | 0,0 | 1,1E-03 | 4,6E-02 |
| A_24_P551842 | ENST00000361789 | 4519 | CYTB | cytochrome b | chrM:000015588-000015646 | -1,0 | 1,1E-03 | 4,6E-02 |
| A_24_P275828 | NM_033419 | 93210 | PGAP3 | post-GPI attachment to proteins 3 | 17 | -0,3 | 1,1E-03 | 4,6E-02 |
| A_23_P416314 | BC034222 | 117245 | HRASLS5 | HRAS-like suppressor family, member 5 | 11 | 0,2 | 1,1E-03 | 4,6E-02 |
| A_23_P137705 | NM_005149 | 9095 | TBX19 | T-box 19 | 1 | -0,1 | 1,1E-03 | 4,6E-02 |
| A_32_P24950 | ENST00000330861 | 158158 | RASEF | RAS and EF-hand domain containing | 9 | -0,1 | 1,1E-03 | 4,6E-02 |
| A_23_P55319 | NM_004475 | 2319 | FLOT2 | flotillin 2 | 17 | -0,5 | 1,1E-03 | 4,6E-02 |
| A_23_P217659 | NM_001018055 | 79184 | BRCC3 | BRCA1/BRCA2-containing complex, subunit 3 | X | 0,5 | 1,1E-03 | 4,6E-02 |
| A_23_P23191 | NM_005529 | 3339 | HSPG2 | heparan sulfate proteoglycan 2 | 1 | -0,2 | 1,1E-03 | 4,6E-02 |
| A_24_P230173 | BC041772 | 124976 | SPNS2 | spinster homolog 2 (Drosophila) | 17 | -0,1 | 1,1E-03 | 4,6E-02 |
| A_23_P10885 | THC2632814 | 7767 | ZNF224 | zinc finger protein 224 | 19 | 0,0 | 1,2E-03 | 4,7E-02 |
| A_23_P386420 | NM_001516 | 2967 | GTF2H3 | general transcription factor IIH, polypeptide 3, 34kDa | 12 | 0,1 | 1,2E-03 | 4,7E-02 |
| A_24_P108451 | NM_000175 | 2821 | GPI | glucose-6-phosphate isomerase | 19 | 0,7 | 1,2E-03 | 4,7E-02 |
| A_23_P73982 | NM_018087 | 55706 | TMEM48 | transmembrane protein 48 | 1 | 0,6 | 1,2E-03 | 4,7E-02 |
| A_23_P38181 | NM_138619 | 23163 | GGA3 | golgi-associated, gamma adaptin ear containing, ARF binding protein 3 | 17 | -0,1 | 1,2E-03 | 4,7E-02 |
| A_23_P28906 | ENST00000251847 | 55614 | KIF16B | kinesin family member 16B | 20 | 0,0 | 1,2E-03 | 4,7E-02 |
| A_24_P214754 | NM_001018077 | 2908 | NR3C1 | nuclear receptor subfamily 3, group C, member 1 (glucocorticoid receptor) | 5 | 0,3 | 1,2E-03 | 4,7E-02 |
| A_24_P88870 | NM_001038628 | 8706 | B3GALNT1 | beta-1,3-N-acetylgalactosaminyltransferase 1 (globoside blood group) | 3 | 0,1 | 1,2E-03 | 4,7E-02 |
| A_23_P152919 | NM_002532 | 4927 | NUP88 | nucleoporin 88kDa | 17 | 0,5 | 1,2E-03 | 4,7E-02 |
| A_23_P160214 | BC028374 | 22996 | TTC39A | tetratricopeptide repeat domain 39A | 1 | -0,1 | 1,2E-03 | 4,7E-02 |
| A_23_P420373 | NM_175630 | 1788 | DNMT3A | DNA (cytosine-5-)-methyltransferase 3 alpha | 2 | 0,1 | 1,2E-03 | 4,7E-02 |
| A_24_P285378 | BC001256 | 56063 | TMEM234 | transmembrane protein 234 | 1 | 0,0 | 1,2E-03 | 4,7E-02 |
| A_24_P200854 | NM_006735 | 3199 | HOXA2 | homeobox A2 | 7 | 0,1 | 1,2E-03 | 4,7E-02 |
| A_23_P167521 | NM_033549 | 90933 | TRIM41 | tripartite motif containing 41 | 5 | -0,1 | 1,2E-03 | 4,7E-02 |
| A_23_P59528 | NM_020186 | 57001 | ACN9 | ACN9 homolog (S. cerevisiae) | 7 | 0,6 | 1,2E-03 | 4,7E-02 |
| A_23_P20777 | NM_033117 | 92400 | RBM18 | RNA binding motif protein 18 | 9 | 0,6 | 1,2E-03 | 4,8E-02 |
| A_23_P120243 | NM_024501 | 3231 | HOXD1 | homeobox D1 | 2 | -0,3 | 1,2E-03 | 4,8E-02 |
| A_23_P153098 | NM_032124 | 84064 | HDHD2 | haloacid dehalogenase-like hydrolase domain containing 2 | 18 | 0,5 | 1,2E-03 | 4,8E-02 |
| A_23_P256342 | NM_015132 | 23161 | SNX13 | sorting nexin 13 | 7 | 0,3 | 1,2E-03 | 4,8E-02 |
| A_23_P94370 | NM_003909 | 8895 | CPNE3 | copine III | 8 | 0,3 | 1,2E-03 | 4,8E-02 |
| A_32_P148672 | NM_006938 | 6632 | SNRPD1 | small nuclear ribonucleoprotein D1 polypeptide 16kDa | 18 | 0,5 | 1,2E-03 | 4,8E-02 |
| A_23_P251095 | NM_000284 | 5160 | PDHA1 | pyruvate dehydrogenase (lipoamide) alpha 1 | X | 0,5 | 1,2E-03 | 4,8E-02 |
| A_23_P30338 | NM_001239 | 902 | CCNH | cyclin H | 5 | 0,5 | 1,2E-03 | 4,8E-02 |
| A_23_P205046 | NM_017664 | 55608 | ANKRD10 | ankyrin repeat domain 10 | 13 | -0,2 | 1,2E-03 | 4,8E-02 |
| A_23_P22957 | NM_016009 | 51100 | SH3GLB1 | SH3-domain GRB2-like endophilin B1 | 1 | 0,5 | 1,2E-03 | 4,8E-02 |
| A_23_P90790 | NM_199227 | 254042 | METAP1D | methionyl aminopeptidase type 1D (mitochondrial) | 2 | 0,1 | 1,2E-03 | 4,8E-02 |
| A_23_P168229 | NM_022085 | 81567 | TXNDC5 | thioredoxin domain containing 5 (endoplasmic reticulum) | 6 | 0,6 | 1,2E-03 | 4,8E-02 |
| A_24_P11791 | NM_002268 | 3840 | KPNA4 | karyopherin alpha 4 (importin alpha 3) | 3 | 0,9 | 1,2E-03 | 4,8E-02 |
| A_23_P156327 | NM_000358 | 7045 | TGFBI | transforming growth factor, beta-induced, 68kDa | 5 | 1,7 | 1,2E-03 | 4,8E-02 |
| A_23_P60028 | NM_005648 | 6921 | TCEB1 | transcription elongation factor B (SIII), polypeptide 1 (15kDa, elongin C) | 8 | 0,6 | 1,2E-03 | 4,8E-02 |
| A_23_P214222 | NM_002356 | 4082 | MARCKS | myristoylated alanine-rich protein kinase C substrate | 6 | 0,9 | 1,2E-03 | 4,8E-02 |
| A_23_P47282 | NM_021978 | 6768 | ST14 | suppression of tumorigenicity 14 (colon carcinoma) | 11 | -0,7 | 1,2E-03 | 4,8E-02 |
| A_32_P3385 | CR611098 | 401264 | FLJ37798 | hypothetical LOC401264 | 6 | -0,1 | 1,2E-03 | 4,8E-02 |
| A_23_P149509 | NM_005341 | 3104 | ZBTB48 | zinc finger and BTB domain containing 48 | 1 | -0,1 | 1,2E-03 | 4,8E-02 |
| A_23_P149259 | NM_032323 | 84283 | TMEM79 | transmembrane protein 79 | 1 | -0,1 | 1,2E-03 | 4,8E-02 |
| A_24_P7887 | NM_001429 | 2033 | EP300 | E1A binding protein p300 | 22 | -0,1 | 1,2E-03 | 4,8E-02 |
| A_23_P407684 | NM_178167 | 90850 | ZNF598 | zinc finger protein 598 | 16 | -0,3 | 1,2E-03 | 4,8E-02 |
| A_23_P371284 | NM_178430 | 353141 | LCE2D | late cornified envelope 2D | 1 | -0,2 | 1,2E-03 | 4,8E-02 |
| A_32_P235872 | NM_005898 | 4076 | CAPRIN1 | cell cycle associated protein 1 | 11 | 0,7 | 1,2E-03 | 4,8E-02 |
| A_23_P87732 | NM_018082 | 55703 | POLR3B | polymerase (RNA) III (DNA directed) polypeptide B | 12 | 0,2 | 1,3E-03 | 4,8E-02 |
| A_32_P182662 | NM_022831 | 64853 | AIDA | axin interactor, dorsalization associated | 1 | 0,1 | 1,3E-03 | 4,8E-02 |
| A_32_P168464 | ENST00000378179 | 8573 | CASK | calcium/calmodulin-dependent serine protein kinase (MAGUK family) | X | -0,5 | 1,3E-03 | 4,8E-02 |
| A_24_P154037 | NM_003749 | 8660 | IRS2 | insulin receptor substrate 2 | 13 | -0,5 | 1,3E-03 | 4,8E-02 |
| A_23_P123622 | NM_003995 | 4882 | NPR2 | natriuretic peptide receptor B/guanylate cyclase B (atrionatriuretic peptide receptor B) | 9 | -0,1 | 1,3E-03 | 4,8E-02 |
| A_23_P20882 | NM_004888 | 9550 | ATP6V1G1 | ATPase, H+ transporting, lysosomal 13kDa, V1 subunit G1 | 9 | -0,7 | 1,3E-03 | 4,8E-02 |
| A_32_P196896 | THC2507805 | 23231 | SEL1L3 | sel-1 suppressor of lin-12-like 3 (C. elegans) | 4 | 0,1 | 1,3E-03 | 4,8E-02 |
| A_23_P25515 | NM_004685 | 9107 | MTMR6 | myotubularin related protein 6 | 13 | 0,0 | 1,3E-03 | 4,8E-02 |
| A_23_P83192 | NM_014172 | 29085 | PHPT1 | phosphohistidine phosphatase 1 | 9 | -0,5 | 1,3E-03 | 4,8E-02 |
| A_23_P259506 | NM_032412 | 84418 | C5orf32 | chromosome 5 open reading frame 32 | 5 | -0,6 | 1,3E-03 | 4,8E-02 |
| A_23_P428849 | NM_144636 | 131474 | CHCHD4 | coiled-coil-helix-coiled-coil-helix domain containing 4 | 3 | 0,4 | 1,3E-03 | 4,8E-02 |
| A_23_P416711 | NM_152996 | 256435 | ST6GALNAC3 | ST6 (alpha-N-acetyl-neuraminyl-2,3-beta-galactosyl-1,3)-N-acetylgalactosaminide alpha-2,6-sialyltransferase 3 | 1 | 0,4 | 1,3E-03 | 4,9E-02 |
| A_23_P500998 | NM_152739 | 3205 | HOXA9 | homeobox A9 | 7 | 0,7 | 1,3E-03 | 4,9E-02 |
| A_23_P28733 | NM_002895 | 5933 | RBL1 | retinoblastoma-like 1 (p107) | 20 | 0,1 | 1,3E-03 | 4,9E-02 |
| A_24_P117620 | NM_018584 | 55450 | CAMK2N1 | calcium/calmodulin-dependent protein kinase II inhibitor 1 | 1 | -0,8 | 1,3E-03 | 4,9E-02 |
| A_32_P87769 | NM_203301 | 254170 | FBXO33 | F-box protein 33 | 14 | 0,5 | 1,3E-03 | 4,9E-02 |
| A_23_P161522 | NM_025124 | 80194 | TMEM134 | transmembrane protein 134 | 11 | -0,6 | 1,3E-03 | 4,9E-02 |
| A_24_P397150 | NM_005255 | 2580 | GAK | cyclin G associated kinase | 4 | -0,6 | 1,3E-03 | 4,9E-02 |
| A_23_P86021 | NM_003944 | 8991 | SELENBP1 | selenium binding protein 1 | 1 | 0,5 | 1,3E-03 | 4,9E-02 |
| A_24_P170660 | NM_015001 | 23013 | SPEN | spen homolog, transcriptional regulator (Drosophila) | 1 | -0,1 | 1,3E-03 | 4,9E-02 |
| A_23_P112596 | NM_001123 | 132 | ADK | adenosine kinase | 10 | 0,4 | 1,3E-03 | 4,9E-02 |
| A_23_P257104 | NM_002486 | 4686 | NCBP1 | nuclear cap binding protein subunit 1, 80kDa | 9 | 0,5 | 1,3E-03 | 4,9E-02 |
| A_23_P372255 | NM_002221 | 3707 | ITPKB | inositol-trisphosphate 3-kinase B | 1 | -0,1 | 1,3E-03 | 4,9E-02 |
| A_24_P5743 | NM_153329 | 126133 | ALDH16A1 | aldehyde dehydrogenase 16 family, member A1 | 19 | -0,2 | 1,3E-03 | 4,9E-02 |
| A_32_P512061 | NR_002188 | 2630 | GBAP1 | glucosidase, beta, acid pseudogene 1 | 1 | -0,2 | 1,3E-03 | 4,9E-02 |
| A_23_P120467 | NM_199427 | 55734 | ZFP64 | zinc finger protein 64 homolog (mouse) | 20 | 0,6 | 1,3E-03 | 4,9E-02 |
| A_23_P201342 | NM_181870 | 1855 | DVL1 | dishevelled, dsh homolog 1 (Drosophila) | 1 | -0,5 | 1,3E-03 | 4,9E-02 |
| A_24_P246196 | NM_214675 | 10332 | CLEC4M | C-type lectin domain family 4, member M | 19 | 0,0 | 1,3E-03 | 4,9E-02 |
| A_23_P52552 | NM_004281 | 9531 | BAG3 | BCL2-associated athanogene 3 | 10 | 0,2 | 1,3E-03 | 4,9E-02 |
| A_23_P46131 | NM_024869 | 79927 | GRRP1 | glycine/arginine rich protein 1 | 1 | 0,0 | 1,3E-03 | 4,9E-02 |
| A_23_P429461 | NM_145059 | 197258 | FUK | fucokinase | 16 | -0,3 | 1,3E-03 | 4,9E-02 |
| A_32_P232523 | THC2659519 | 23230 | VPS13A | vacuolar protein sorting 13 homolog A (S. cerevisiae) | 9 | -0,1 | 1,3E-03 | 4,9E-02 |
| A_24_P212457 | NM_032292 | 54856 | GON4L | gon-4-like (C. elegans) | 1 | -0,3 | 1,3E-03 | 4,9E-02 |
| A_23_P320261 | NM_001035516 | 93099 | DMKN | dermokine | 19 | -0,8 | 1,3E-03 | 4,9E-02 |
| A_24_P6083 | NM_213720 | 400916 | CHCHD10 | coiled-coil-helix-coiled-coil-helix domain containing 10 | 22 | -0,5 | 1,3E-03 | 4,9E-02 |
| A_23_P63681 | NM_004969 | 3416 | IDE | insulin-degrading enzyme | 10 | 0,2 | 1,3E-03 | 4,9E-02 |
| A_32_P192823 | NM_175886 | 221823 | PRPS1L1 | phosphoribosyl pyrophosphate synthetase 1-like 1 | 7 | 0,2 | 1,3E-03 | 4,9E-02 |
| A_24_P246573 | NM_015209 | 23254 | KAZN | kazrin, periplakin interacting protein | 1 | -0,2 | 1,3E-03 | 4,9E-02 |
| A_23_P107401 | NM_003255 | 7077 | TIMP2 | TIMP metallopeptidase inhibitor 2 | 17 | 0,6 | 1,3E-03 | 4,9E-02 |
| A_24_P333525 | NM_014857 | 9910 | RABGAP1L | RAB GTPase activating protein 1-like | 1 | 0,6 | 1,3E-03 | 4,9E-02 |
| A_23_P86403 | NM_004521 | 3799 | KIF5B | kinesin family member 5B | 10 | 0,3 | 1,3E-03 | 4,9E-02 |
| A_23_P152727 | NM_014798 | 9842 | PLEKHM1 | pleckstrin homology domain containing, family M (with RUN domain) member 1 | 17 | -0,5 | 1,3E-03 | 4,9E-02 |
| A_23_P306105 | NM_020474 | 2589 | GALNT1 | UDP-N-acetyl-alpha-D-galactosamine:polypeptide N-acetylgalactosaminyltransferase 1 (GalNAc-T1) | 18 | 0,5 | 1,3E-03 | 4,9E-02 |
| A_24_P71700 | NM_145166 | 92999 | ZBTB47 | zinc finger and BTB domain containing 47 | 3 | -0,4 | 1,3E-03 | 4,9E-02 |
| A_23_P138693 | NM_004808 | 9397 | NMT2 | N-myristoyltransferase 2 | 10 | 0,2 | 1,3E-03 | 4,9E-02 |
| A_24_P378506 | NM_023079 | 65264 | UBE2Z | ubiquitin-conjugating enzyme E2Z | 17 | -0,4 | 1,3E-03 | 4,9E-02 |
| A_23_P34115 | NM_004135 | 3421 | IDH3G | isocitrate dehydrogenase 3 (NAD+) gamma | X | -0,3 | 1,3E-03 | 4,9E-02 |
| A_23_P23526 | NM_033020 | 51592 | TRIM33 | tripartite motif containing 33 | 1 | -0,3 | 1,3E-03 | 4,9E-02 |
| A_23_P56140 | NM_001319 | 1455 | CSNK1G2 | casein kinase 1, gamma 2 | 19 | -0,3 | 1,3E-03 | 4,9E-02 |
| A_24_P225970 | NM_001012409 | 151648 | SGOL1 | shugoshin-like 1 (S. pombe) | 3 | 0,2 | 1,3E-03 | 4,9E-02 |
| A_24_P406693 | NM_000917 | 5033 | P4HA1 | prolyl 4-hydroxylase, alpha polypeptide I | 10 | 0,4 | 1,3E-03 | 4,9E-02 |
| A_23_P7144 | NM_001511 | 2919 | CXCL1 | chemokine (C-X-C motif) ligand 1 (melanoma growth stimulating activity, alpha) | 4 | -2,5 | 1,4E-03 | 4,9E-02 |
| A_24_P115183 | NM_001305 | 1364 | CLDN4 | claudin 4 | 7 | -0,6 | 1,4E-03 | 4,9E-02 |
| A_23_P218423 | NM_148887 | 124995 | MRPL10 | mitochondrial ribosomal protein L10 | 17 | -0,4 | 1,4E-03 | 4,9E-02 |
| A_23_P130304 | NM_006701 | 10907 | TXNL4A | thioredoxin-like 4A | 18 | 0,6 | 1,4E-03 | 4,9E-02 |
| A_24_P152527 | NM_006373 | 10493 | VAT1 | vesicle amine transport protein 1 homolog (T. californica) | 17 | -0,3 | 1,4E-03 | 4,9E-02 |
| A_24_P230907 | BC015394 | 317662 | FAM149B1 | family with sequence similarity 149, member B1 | 10 | 0,3 | 1,4E-03 | 4,9E-02 |
| A_24_P595460 | AK097398 | 4925 | NUCB2 | nucleobindin 2 | 11 | 0,2 | 1,4E-03 | 4,9E-02 |
| A_23_P78438 | NM_018255 | 55250 | ELP2 | elongation protein 2 homolog (S. cerevisiae) | 18 | 0,5 | 1,4E-03 | 4,9E-02 |
| A_23_P330209 | NM_057178 | 117584 | RFFL | ring finger and FYVE-like domain containing 1 | 17 | 0,1 | 1,4E-03 | 4,9E-02 |
| A_23_P217009 | NM_032596 | 84688 | C9orf24 | chromosome 9 open reading frame 24 | 9 | 0,1 | 1,4E-03 | 4,9E-02 |
| A_23_P200493 | NM_002296 | 3930 | LBR | lamin B receptor | 1 | 1,0 | 1,4E-03 | 4,9E-02 |
| A_23_P427075 | NM_004937 | 1497 | CTNS | cystinosin, lysosomal cystine transporter | 17 | -0,2 | 1,4E-03 | 4,9E-02 |
| A_23_P65370 | NM_016417 | 51218 | GLRX5 | glutaredoxin 5 | 14 | 0,5 | 1,4E-03 | 4,9E-02 |
| A_32_P14187 | NM_001032280 | 7020 | TFAP2A | transcription factor AP-2 alpha (activating enhancer binding protein 2 alpha) | 6 | 0,0 | 1,4E-03 | 4,9E-02 |
| A_23_P137035 | NM_003662 | 8544 | PIR | pirin (iron-binding nuclear protein) | X | 0,9 | 1,4E-03 | 4,9E-02 |
| A_23_P162787 | NM_006704 | 10910 | SUGT1 | SGT1, suppressor of G2 allele of SKP1 (S. cerevisiae) | 13 | 0,8 | 1,4E-03 | 4,9E-02 |
| A_23_P35617 | NM_016341 | 51196 | PLCE1 | phospholipase C, epsilon 1 | 10 | 0,3 | 1,4E-03 | 4,9E-02 |
| A_24_P181108 | NM_018093 | 54663 | WDR74 | WD repeat domain 74 | 11 | -0,5 | 1,4E-03 | 4,9E-02 |
| A_24_P851353 | BC047230 | 100506388 | LOC100506388 | hypothetical LOC100506388 | 17 | 0,0 | 1,4E-03 | 4,9E-02 |
| A_32_P143000 | ENST00000261275 | 23359 | FAM189A1 | family with sequence similarity 189, member A1 | 15 | -0,5 | 1,4E-03 | 5,0E-02 |
| A_32_P10960 | NM_015262 | 253725 | FAM21C | family with sequence similarity 21, member C | 10 | -0,3 | 1,4E-03 | 5,0E-02 |
| A_24_P325046 | ENST00000322831 | 84186 | ZCCHC7 | zinc finger, CCHC domain containing 7 | 9 | 0,1 | 1,4E-03 | 5,0E-02 |
| A_23_P406131 | NM_020422 | 57146 | TMEM159 | transmembrane protein 159 | 16 | -1,0 | 1,4E-03 | 5,0E-02 |
| A_23_P83453 | NM_003074 | 6599 | SMARCC1 | SWI/SNF related, matrix associated, actin dependent regulator of chromatin, subfamily c, member 1 | 3 | -0,5 | 1,4E-03 | 5,0E-02 |
| A_23_P139260 | NM_183233 | 5002 | SLC22A18 | solute carrier family 22, member 18 | 11 | -0,7 | 1,4E-03 | 5,0E-02 |
