# Supplementary material for: Expression Signature as a Biomarker for Prenatal Diagnosis of Trisomy 21
Source: PLoS One. 2013 Sep 16;8(9):e74184. doi: 10.1371/journal.pone.0074184 (PMC3774664; doi:10.1371/journal.pone.0074184)
Supplement: Table S4 — Clinical characteristics of the amniotic fluid samples (controls, T21) used for global expression profiling. (DOCX) [file pone.0074184.s006.docx]

Supplementary Table 4. Clinical characteristics of the amniotic fluid samples (controls, T21) used for global expression profiling.

| Sample ID | karyotype | Ultrasound scan/ indication for karyotyping |
| --- | --- | --- |
| 2 | 46,XY (N) | normal |
| 9 | 46,XX (N) | normal |
| 11 | 46,XY (N) | normal |
| 12 | 46,XY (N) | normal |
| 14 | 46,XX (N) | normal |
| 15 | 46,XX (N) | normal |
| 16 | 46,XX (N) | normal |
| 19 | 46,XY (N) | normal |
| 20 | 46,XY (N) | normal |
| 4 | 47,XY,+21 | NMCA^#^ |
| 5 | 47,XY,+21 | Increased nuchal translucency |
| 6 | 47,XY,+21 | Cystic hygroma colli |
| 7 | 47,XX,+21 | NMCA^#^ |
| 12 | 47,XX,+21 | NMCA^#^ |
| 17 | 47,XY,+21 | Increased nuchal translucency |
| 21 | 47,XX,+21 | Increased nuchal translucency+ double test |
| 22 | 47,XX,+21 | Congenital heart defect, intrauterine growth retardation |
| 24 | 47,XY,+21 | Cystic hygroma colli |
| 27 | 47,XX,+21 | NMCA^#^ |

Note. NMCA^#^ - no major congenital anomalies were detected on routine morphological ultrasound scan at 16-18 gestation weeks (samples were collected from women attending chromosomal investigation because of the advanced maternal age). 46,XY (normal male karyotype), 46,XX (normal female karyotype), 47,XY,+21 (male karyotype with trisomy 21), 47,XX,+21 (female karyotype with trisomy 21).
