# Supplementary material for: Expression Signature as a Biomarker for Prenatal Diagnosis of Trisomy 21
Source: PLoS One. 2013 Sep 16;8(9):e74184. doi: 10.1371/journal.pone.0074184 (PMC3774664; doi:10.1371/journal.pone.0074184)
Supplement: Table S5 — Clinical characteristics of the amniotic fluid samples with T21 samples used for validation in real-time quantification step. (DOCX) [file pone.0074184.s007.docx]

Supplementary Table 5. Clinical characteristics of the amniotic fluid samples with T21 samples used for validation in real-time quantification step.

|  | karyotype | Ultrasound scan/ indication for karyotyping |
| --- | --- | --- |
| 365A | 47,XY,+21 | NMCA^#^ |
| 364A | 47,XY,+21 | NMCA^#^ |
| 311A | 47,XY,+21 | NMCA^#^ |
| 39A | 47,XY,+21 | increased nuchal translucency |
| 291A | 46,XY,der(21;21)(q10;q10)dn | increased nuchal translucency + double test |
| 47A | 47,XY,+21 | Cystic hygroma colli |
| 278A | 47,XX,+21 | Cystic hygroma colli, hydrops fetalis |
| 296A | 47,XY,+21 | increased nuchal translucency |
| 124A | 47,XX,+21 | increased nuchal translucency + double test |
| 81A | 47,XY,+21 | increased nuchal translucency |
| 101A | 47,XX,+21 | increased nuchal translucency + double test |
| 33A | 47,XY,+21 | NMCA^#^ |
| 164A | 47,XX,+21 | NMCA^#^ |
| 189A | 46,XY,der(21;21)(q10;q10)dn | increased nuchal translucency |
| 138A | 47,XX,+21 | NMCA^#^ |
| 349A | 47,XY,+21 | increased nuchal translucency |

Note. NMCA^#^ - no major congenital anomalies were detected on routine morphological ultrasound scan at 16-18 gestation weeks (samples were collected from women attending chromosomal investigation because of the advanced maternal age). 46,XY (normal male karyotype), 46,XX (normal female karyotype), 47,XY,+21 (male karyotype with trisomy 21), 47,XX,+21 (female karyotype with trisomy 21).
